# Migration of Hydride, Methyl, and Chloride Ligands between Al and M in (PAlP)M Pincer Complexes (M = Rh or Ir)

**DOI:** 10.1021/acs.organomet.3c00359

**Published:** 2023-10-24

**Authors:** Vinh T. Nguyen, Qingheng Lai, Naphol Witayapaisitsan, Nattamai Bhuvanesh, Panida Surawatanawong, Oleg V. Ozerov

**Affiliations:** † Department of Chemistry, Texas A&M University, 3255 TAMU, College Station, Texas 77842, United States; ‡ Department of Chemistry and Center of Excellence for Innovation in Chemistry, Faculty of Science, Mahidol University, Bangkok 10400, Thailand

## Abstract

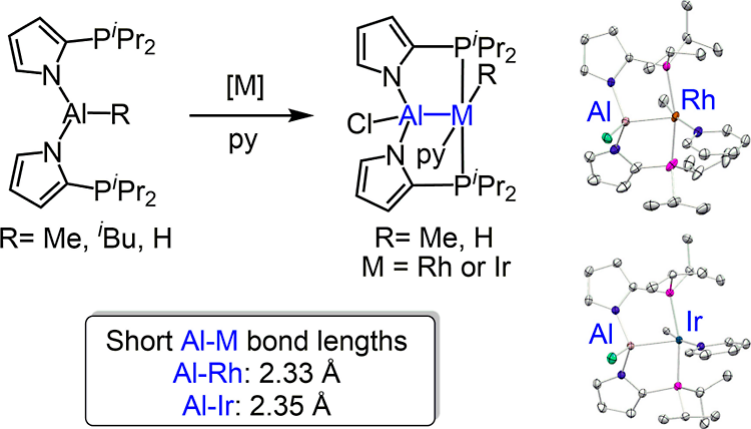

Protolysis of AlMe_3_ or AlBu^i^_3_ with 2-diisopropylphosphinopyrrole (1) yields molecules containing two flanking phosphines and a central Al–Me (2-Me), Al-^i^Bu (2-^i^Bu), or Al–H (2-H) unit. The reactions of 2-Me with [L_2_MCl]_2_ (L = cyclooctene or 1/2 1,5-cyclooctadiene and M = Rh or Ir) in the presence of pyridine produces PAl^Cl^P pincer complexes (3-Rh and 3-Ir) with Al–Cl and M–Me bonds. The analogous reaction of a mixture of 2-^i^Bu and 2-H with [L_2_MCl]_2_ and pyridine resulted in the formation of analogous Rh–H (4-Rh) and Ir–H (4-Ir) complexes. Treatment of 3-Rh with NaBEt_3_H produced compound 5-Rh with an Al–Me and a Rh–H bond; the analogous reaction of 3-Ir did not result in a clean product. 4-Ir accepted an equivalent of H_2_ to produce 6-Ir with two terminal Ir–H bonds and one bridging Al–H–Ir moiety, whereas 4-Rh did not react with H_2_. The density functional theoretical treatment is in accord with this finding, highlights the likely mechanism for the H_2_ addition, and supports the bonding picture in 6-Ir arising from NMR and X-ray diffraction (XRD) observations. Spectroscopic data and XRD studies are consistent with distorted square-pyramidal structures (about Rh or Ir) for compounds 3–5, with an alane occupying the apical position. Complexes 3 and 4 possess some of the shortest known Rh–Al or Ir–Al distances.

## Introduction

**Figure 1:**
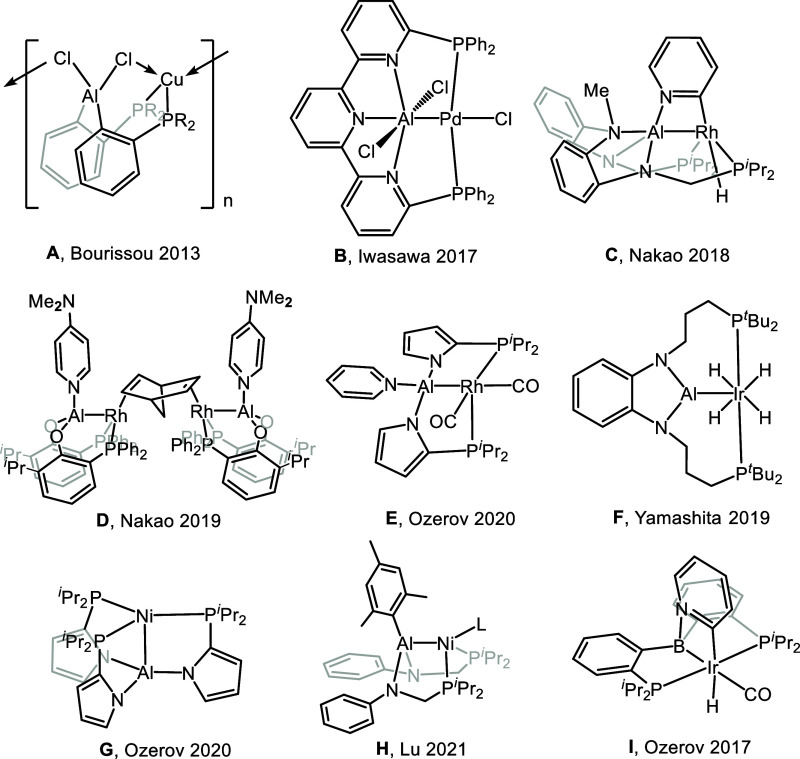
Selected previously reported complexes of multidentate ligands with a central aluminum or boron anchor.

Transition-metal (TM) complexes supported by aluminum-centered polydentate ligands have been attracting increased attention over the past decade. The interest can arguably be attributed to three general features. First, there exists a diversity of modes of interaction between Al and TM, with different modes often accessible within the same ligand framework. Thus, compound **A** (Figure 1) contains a central aluminate, without a direct Al–Cu bond and a bridging chloride instead.^1^ Compounds **B**–**E** can be viewed as containing an aluminyl X-type^2^ ligand bound to TM, with additional donors bound to Al.^3−6^ Compound **F** was billed as an aluminyl–Ir complex with a donor-free Al, although the presence of close Al–H contacts complicates this analysis.^7^ Compounds **G** and **H** contain an alane Z-type ligand bound to a TM. Compounds of the **H** type were observed to undergo exchange of L and X-type substituents between Al and Ni, thus formally converting the Al ligand between a Z-type and an X-type.^8^

Second, since Al is less electronegative than late TMs, the TM-Al bonding may possess unusual characteristics.^9−11^ In particular, the TM^δ−^-Al^δ+^ polarization in metal aluminyl contrasts with that of traditional TM-X interactions.^12,13^ Third, although aluminyl (X) and alane (Z) ligands qualitatively share the above features with boryl/borane ligands,^14,15^ Al engenders higher Lewis acidity. Furthermore, in firm contrast to boron, Al can attain coordination numbers above four (i.e., **B**, **C**),^3,4^ highlighting an alternative avenue for tuning the metal center’s reactivity.

On top of providing fundamental insights, the complexes of Al-centered ligands have already shown potential for unique reactivity. The Nakao group has used systems based on **C** to effect highly regioselective catalytic olefin insertion into a pyridine *ortho* C–H bond,^4,16^ as well as catalysis of the magnesiation of aryl fluorides.^17^ The Peters group explored the use of alane-supported Fe complexes in N_2_ reduction.^18^

Our group previously reported on the complexes of boryl/bis(phosphine) PBP pincer ligands,^14,19−22^ with a particular focus on the selective C–H activation of azines.^23−25^ Similarly to Nakao’s system **C**,^4^ the system **I** possessed nearly perfect selectivity for the *ortho* C–H bond in pyridines and quinoline.^23^ As an expansion of our studies, we became interested in analogous but Al-centered ligands, which connect the Al and the outer phosphine donors via the 1,2-pyrrolidinyl linker. Compound **E** is an example^6^ of a bipodal pincer-type ligand with an aluminyl central moiety and **G** is an example of a tripodal ligand built around an alane Z-type unit.^26^ However, in the pincer manifold, the synthetic approach to **E** was amenable only to Rh and could result in only a saturated dicarbonyl complex. We were interested in accessing Rh and Ir PAlP complexes without carbonyl ligands, and our work on their synthesis and characterization is presented in this report.

## Results and Discussion

### Synthesis of PAlP Ir and Rh Complexes

**Scheme 1:**
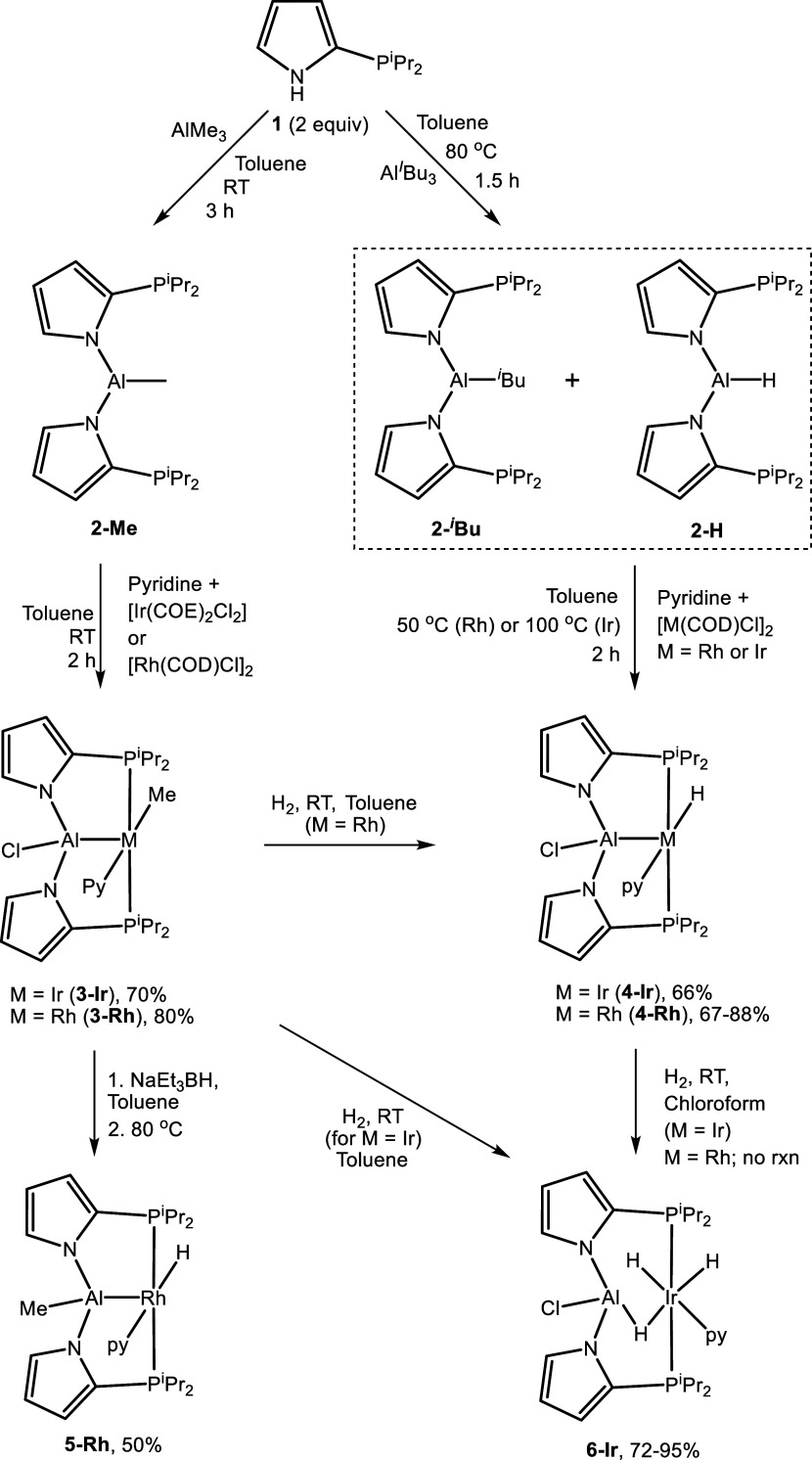
Synthesis of PAlP Rh and Ir Complexes

We previously disclosed that thermolysis of 2-diisopropylphosphinopyrrole (**1**) with AlMe_3_ in a 3:1 P/Al ratio results in the formation of the tripodal AlP_3_ ligand seen in **G**. Here, we report that reactions of **1** with AlMe_3_ or AlBu^i^_3_ in a 2:1 P/Al ratio proceed by analogous protolysis and yield proto-pincer products (**2**, Scheme 1) with high selectivity. The reaction with AlMe_3_ produced >95% **2-Me**, with concomitant loss of methane. The reaction with AlBu^i^_3_ produced a mixture of ca. 95% **2-**^***i***^**Bu** and **2-H** in a 79:16 ratio, with the observation of isobutane and isobutene. Commercial AlBu^i^_3_ may contain (or develop upon storage or heating) some isobutene from β-hydrogen elimination to give HAlBu^i^_2_.^27^**2-H** could originate from the protolysis of the Al–C bond in the latter. Observation of a broadened ^27^Al{^1^H} NMR signal at 146.6 ppm suggested a four-coordinate Al in **2-Me**,^28^ likely either by coordination of phosphines to Al or via bridging *N*-pyrrolides.

Solutions of in situ prepared **2-Me** and the **2-**^***i***^**Bu**/**2-H** mixture served well as precursors for the formation of PAlP complexes. Addition of 1 equiv of pyridine and either [Rh(COD)Cl]_2_ or [Ir(COE)_2_Cl]_2_ to **2-Me** resulted in the formation of **3-Rh** and **3-Ir**, respectively. The analogous reaction utilizing **2-**^***i***^**Bu**/**2-H** and [M(COD)Cl]_2_ (M = Rh or Ir) led to the formation of **4-Rh** or **4-Ir**. Ostensibly, transfer of the *i*-butyl group of **2-**^***i***^**Bu** to Rh or Ir results in rapid β-H elimination; thus, **2-**^***i***^**Bu** and **2-H** are operationally equivalent as PAlP sources here. It is notable that in the reactions with **2**, the original substituent on Al is transferred to the TM, while the originally Rh/Ir-bound chloride ends up on Al. The facile transfer of X-type ligands between Al and the TM here is related to the observations by Lu et al. with compound **H**.^8^ We also reported on the exchange of X-type ligands between boron and Rh/Ir in the system derived from compound **I**.^23,24^

Thermolysis of **3-Rh** with NaEt_3_BH permitted the isolation of **5-Rh** in a 50% yield. The analogous reaction of **3-Ir** produced a complicated mixture with no clear major product. The modest isolated yield of **5-Rh** is partly due to its thermal instability. Thermolysis of a pure sample of **5-Rh** at 100 °C in C_6_D_6_ resulted in decomposition to multiple products with an apparent half-life of ca. 10 h alongside methane gas (see Figures S40 and S41).

Interestingly, in **5-Rh**, the Me group originally attached to Rh in **3-Rh** has migrated to Al. The distribution of the non-PAlP ligands and substituents between Al, Rh, and Ir is likely governed by thermodynamics, with Al favoring the more electronegative X-type substituent.

Hydrogenolysis of the M-Me bond in **3** under a H_2_ atmosphere led to different outcomes for **3-Rh** vs **3-Ir**: the Rh reaction produced the monohydride compound **4-Rh**, but with Ir, the product was the trihydride compound **6-Ir**. In concordance with these results, treatment of **4-Ir** with H_2_ produced **6-Ir**, but exposure of **4-Rh** to a H_2_ atmosphere did not result in a significant change in the observed NMR spectra.

### NMR Spectroscopic Characterization

Key NMR data for compounds **3**–**6** are summarized in Table 1. We were able to observe the ^27^Al NMR signals for the Ir and Rh complexes. For compounds **3** and **4**, the Ir analogues resonate about 30 ppm upfield, but for the same TM, the ^27^Al NMR chemical shifts are quite similar, consistent with the same immediate coordination environment about Al in **3** and **4**. The ^27^Al NMR chemical shifts for **5-Rh** and **6-Ir** are 30–35 ppm different from those of **3/4** with the corresponding metal, also consistent with the different immediate environment about Al in **5** or **6**.

**Table 1:** Chemical Shifts (in ppm) and *J* Values (Hz) from the ^31^P{^1^H} NMR, ^27^Al{^1^H} NMR, ^13^C{^1^H} NMR of M–*Me*, and ^1^H NMR of M–C*H*_3_ and M–*H* for Rh and Ir Complexes

compound	^31^P (*J*)	^27^Al	M–*C*H_3_ (*J*)	M–C*H*_3_ (*J*)	M–*H* (*J*)
**3-Rh**	33.7	127.6	–3.7	0.29	
(C_6_D_6_)	(^1^*J*_P,Rh_ = 120)		(d, ^1^*J*_C,Rh_ = 22)	(td, ^2^*J*_H,Rh_ = 2, ^3^*J*_HP_ = 7)	
**3-Ir**	31.1	96.8	–18.4	0.86	
(C_6_D_6_)			(t, ^2^*J*_C,P_ = 6)^a^	(m)	
**4-Rh**	34.2	125.1			–17.96 (q, ^2^*J*_H,P_ ≈ ^1^*J*_H,Rh_ = 20)
(C_6_D_6_)	(^1^*J*_P,Rh_ = 121)				
**4-Ir**	36.3	95.9^a^			–20.30 (t, ^2^*J*_H,P_ = 17.2)
(C_6_D_6_)					
**5-Rh**	35.1	155.9	–4.9	–0.39	–18.17 (dt, ^1^*J*_H,Rh_ = 23.8, ^2^*J*_H,P_ = 18.7)
(CDCl_3_)	(^1^*J*_P,Rh_ = 125)		(br s)	(s)	
**6-Ir**	19.9	120.9			–6.12 (br s), −9.97 (qd, ^2^*J*_H,P_ ≈ ^2^*J*_H,H_ = 14, ^2^*J*_H,H_ = 4), −22.18 (td, ^2^*J*_H,H_ = 4, ^2^*J*_H,P_ = 15)
(CDCl_3_)					

a Solvent is CDCl_3_.

The presence of the Rh- or Ir-bound CH_3_ group in **3-Rh** and **3-Ir** is supported by the observation of the coupling of the ^1^H and of the ^13^C{^1^H} NMR resonance of the CH_3_ group to the pair of equivalent ^31^P nuclei, and in the case of **3-Rh**– to the ^103^Rh nucleus. In contrast, the Al-bound CH_3_ group in **5-Rh** gives rise to a broad, featureless ^13^C{^1^H} NMR resonance and to a singlet with no discernible fine structure in the ^1^H NMR spectrum.

The ^1^H NMR hydride resonances in **4-Rh** and **4-Ir** display the expected coupling to the two ^31^P nuclei and the ^103^Rh nucleus for **3-Rh**. The observed chemical shifts are consistent with a hydride *trans* to a nitrogenous donor.^29,30^**6-Ir** displays three distinct hydridic resonances at ambient temperature. Two of them are sharp, and their fine structure betrays a 4 Hz coupling between them, as well as a coupling to the two ^31^P nuclei. The third is a broad signal at −4.9 ppm, which we tentatively assign as a hydride bridging Al and Ir. This conception of the structure of **6-Ir** is corroborated by X-ray and DFT studies (vide infra).

Compounds **3-Rh** and **3-Ir** display two separate ^1^H NMR resonances for the 2- and 6-positions of the pyridine ligand, whereas all the other compounds show only a single resonance of intensity 2H. The hydrogens in the 3- and 5-positions give rise to a broad singlet of intensity 2H for **3-Rh** and **3-Ir** but a sharp singlet for the other compounds. We believe that the rotation of pyridine about the M–N bond is restricted and is slowest in compounds **3**. We previously analyzed this phenomenon for Ph (and other C-bound aryl) ligands bound to a TM center *cis* to two-PPr^i^_2_ arms.^19,31^ This arrangement “sandwiches” the aryl ring between a pair of Me groups of opposing -PPr^i^_2_ arms and increases the rotational barrier. The loss of 2:2:1 symmetry for a C_6_H_5_ group at ambient temperature is almost always observed in this environment. The lack of this effect in compounds **4**–**6** may be owing to the longer M–N_pyridine_ distance compared to the M–C_Ph_ distance. Compounds **3-Rh** and **3-Ir** may possess a higher rotational barrier because the Me group *trans* to the pyridine is slightly larger than the H ligand in compounds **4**–**5** and may cause the *i*-propyl groups to lean away from M-Me and toward pyridine to a greater degree. The contact between the *i*-propyl groups and the pyridine/aryl ring tends to shift the ^1^H NMR resonances of the *i*-propyl CH_3_ group upfield because of the aromatic ring current effect.

Notably, we observe a significantly more upfield ^1^H NMR resonance for the pair of *i*-propyl methyls in **3-Rh** or **3-Ir** (0.29 and 0.38 ppm) than that in compounds **4**–**5** (0.7–0.8 ppm). The structural studies (vide infra) appear to corroborate this analysis, showing slightly larger P–Rh/Ir–P angles in **3-Rh** and **3-Ir** (163–164°) than in **4-Rh** (ca. 161°).

### X-ray Structural Studies

**Figure 2:**
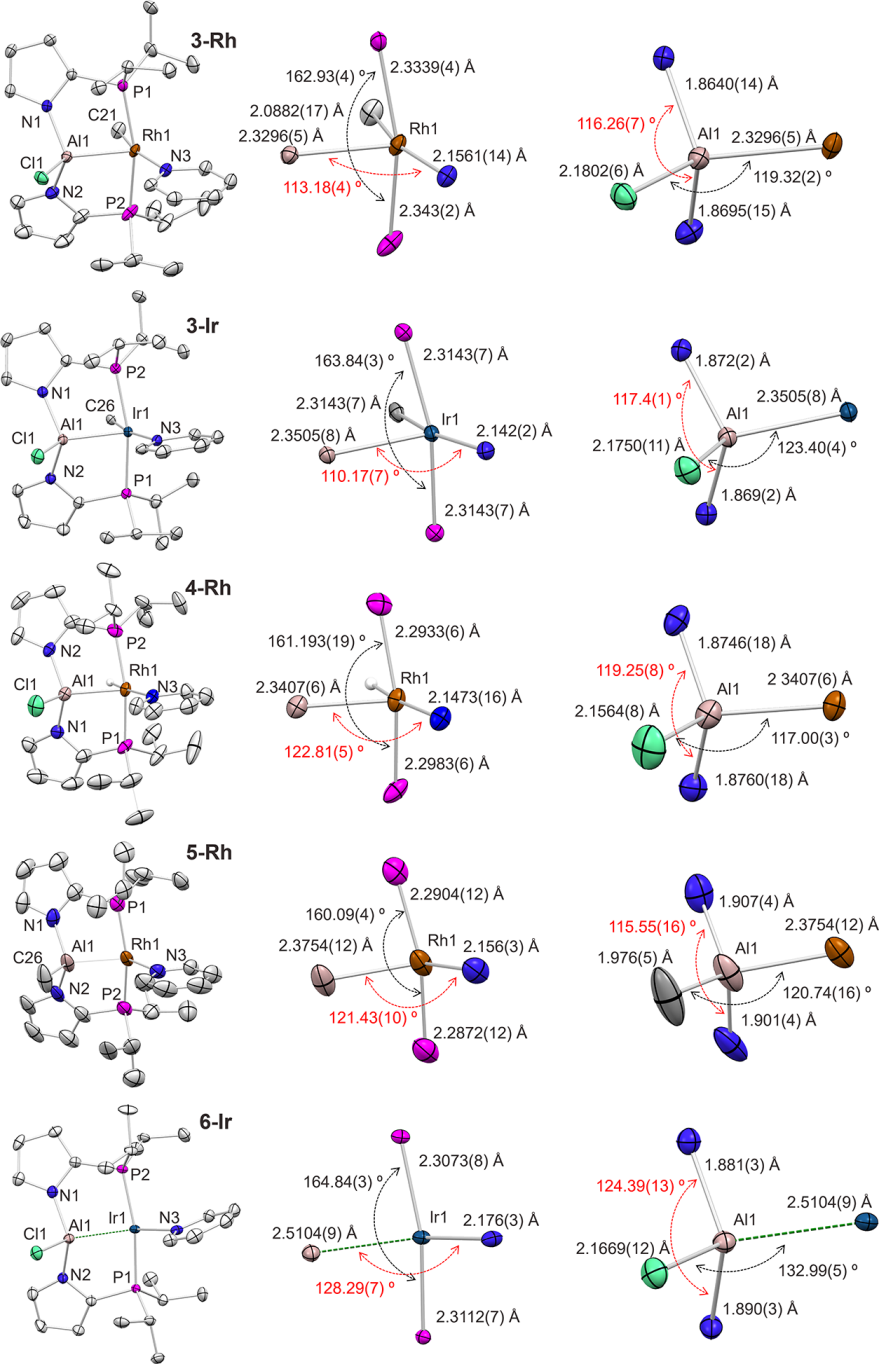
Mercury-rendered ORTEPs (50% thermal ellipsoids) of **3-Rh**, **3-Ir**, **4-Rh**, **5-Rh**, and **6-Ir** (top to bottom). Hydrogen atoms (excluding metal hydrides) have been omitted for clarity. The middle column displays the immediate Rh or Ir coordination environment. The right column displays the immediate Al coordination environment.

Solid-state structures of **3-Rh**, **3-Ir**, **4-Rh**, **5-Rh**, and **6-Ir** were determined by single-crystal X-ray crystallography (Figure 2). The environment of the TM in the structures of **3-Rh**, **3-Ir**, **4-Rh**, and **5-Rh** is five-coordinate and could be described as distorted square-pyramidal. Complexes **3**–**5** could be analyzed as containing a central Z-type alane ligand binding axially to a square-planar M^I^ fragment. Through such binding, the alane Lewis acid engages a filled d_z2_ orbital at the metal and can be viewed to result in a complex containing a trivalent^32,33^ d^6^ Rh/Ir center. A square pyramid, with various distortions, is one of the typical geometries for five-coordinate d^6^ complexes.^34^ The distortion in the structures obtained here is at least partially due to the constraint of the PAlP pincer framework.

The covalent radii of Rh (1.41 Å) and Ir (1.42 Å) are essentially the same,^35^ so it is possible to cross-compare the Rh/Ir-ligand bond distances. The Rh/Ir–P, Rh/Ir–N(pyridine), and Al–N(pyrrolyl) distances vary little among the studied compounds and are generally unremarkable. The Ir–Al distance in **6-Ir** (ca. 2.51 Å) is much longer than in **3**–**5**, consistent with a different structural type for **6-Ir**.

The M-Al distances in compounds **3-Rh**, **3-Ir**, and **4-Rh** fall within a narrow 2.33–2.35 Å range. The slightly longer Rh–Al distance (ca. 2.38 Å) in **5-Rh** is likely a consequence of a somewhat weaker Lewis acidity of the Me-substituted alane vs the Cl-substituted alane in **3**. The Al center in **4-Rh** and **5-Rh** is shifted away from the pyridine (Al1–Rh–N3 angles of ca. 121–123°) compared to the structures of **3-Rh** and **3-Ir** (Al1–Rh/Ir–N3 angles of ca. 110–113°) and thus closer to the hydride. It is possible that this is merely a reflection of different steric pressures by Me versus H; however, it may also be a reflection of a weak Al–H interaction supported by the natural bond orbital (NBO) analysis (vide infra).

The Rh–Al distance in Nakao’s compound **D** (2.3183(8)Å)^5^ may be the shortest known Rh–Al distance, while the Ir–Al distance in Yamashita’s **F** (2.3819(14) Å) was billed as the shortest Ir–Al distance to date, making the Rh/Ir–Al distance in compounds **3**–**5** among the shortest ever determined. Unlike **3**–**5**, **D** and **F** cannot be analyzed as formed via a lone pair donation from M(I) to an AlX_3_ unit. For a closer comparison, Braunschweig’s Cp(Me_3_P)_2_Rh–AlCl_3_^36^ possesses a longer 2.425(1) Rh–Al bond, albeit this molecule is rather pseudo-octahedral about Rh than square-pyramidal. The sum of covalent radii of Al and Rh (or Ir) is 2.62 Å (or 2.63 Å), thus the formal bond shortness ratio^37^ for **3**–**5** is 0.89–0.90. This is smaller than the 0.93 value in the (AlP_3_)Ni complex **G**, likely reflecting the more electron-rich nature of monovalent Rh/Ir vs zerovalent Ni toward an AlX_3_ σ-acceptor. The shortness of the Rh/Ir–Al bonds in **3**–**5** is likely a consequence of the tight chelate constraint and the electron-poor nature of the bis(*N*-pyrrolyl)Al-X moieties.

### DFT Studies

We performed density functional calculations to gain insight into the electronic structures and reactivity of the (PAlP)Rh and (PAlP)Ir complexes. Geometry optimizations in the gas phase were calculated using M06/SDD/6-311G(d,p) with the solvent-corrected free energies in toluene (see details in the Supporting Information).^38^ The optimized geometries of **3-Rh**, **4-Rh**, and **6-Ir** were in agreement with the structures determined by XRD (Tables S5 and S6). The net hydrogenolysis of **3-M** to **4-M** (and methane) was calculated to be decidedly exoergic for both Rh and Ir, with very similar free reaction energies (−30.5 kcal/mol for Rh and −31.0 kcal for Ir; Figure S48).

**Figure 3:**
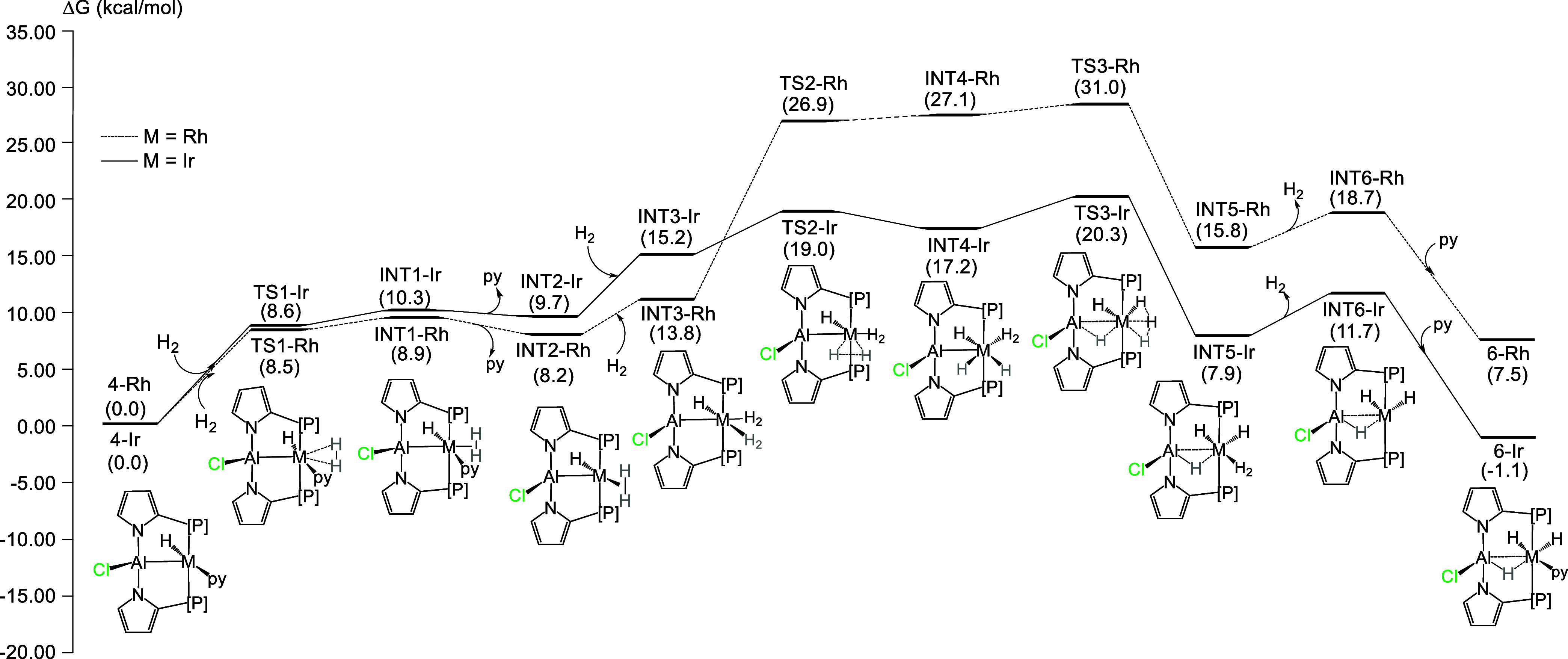
DFT-favored mechanism for the addition of H_2_ to **4-Rh** (dashed line) and **4-Ir** (solid line). Solvent-corrected relative free energies are given in parentheses (in kcal/mol, relative to **4-M** at zero).

The net conversion of **4-M** to **6-M** is a simple addition of H_2_. DFT results (Figure 3) suggest that this process is thermodynamically favorable for Ir (by 1.1 kcal/mol) but not for Rh (unfavorable by 7.5 kcal/mol), and this alone may account for the experimental observation of **6-Ir** but not **6-Rh**. However, the geometry of **4-M** is such that direct addition of H_2_ cannot lead to the geometry of **6-M**. Thus, we undertook a theoretical study of the mechanism of this reaction.

**Figure 4:**
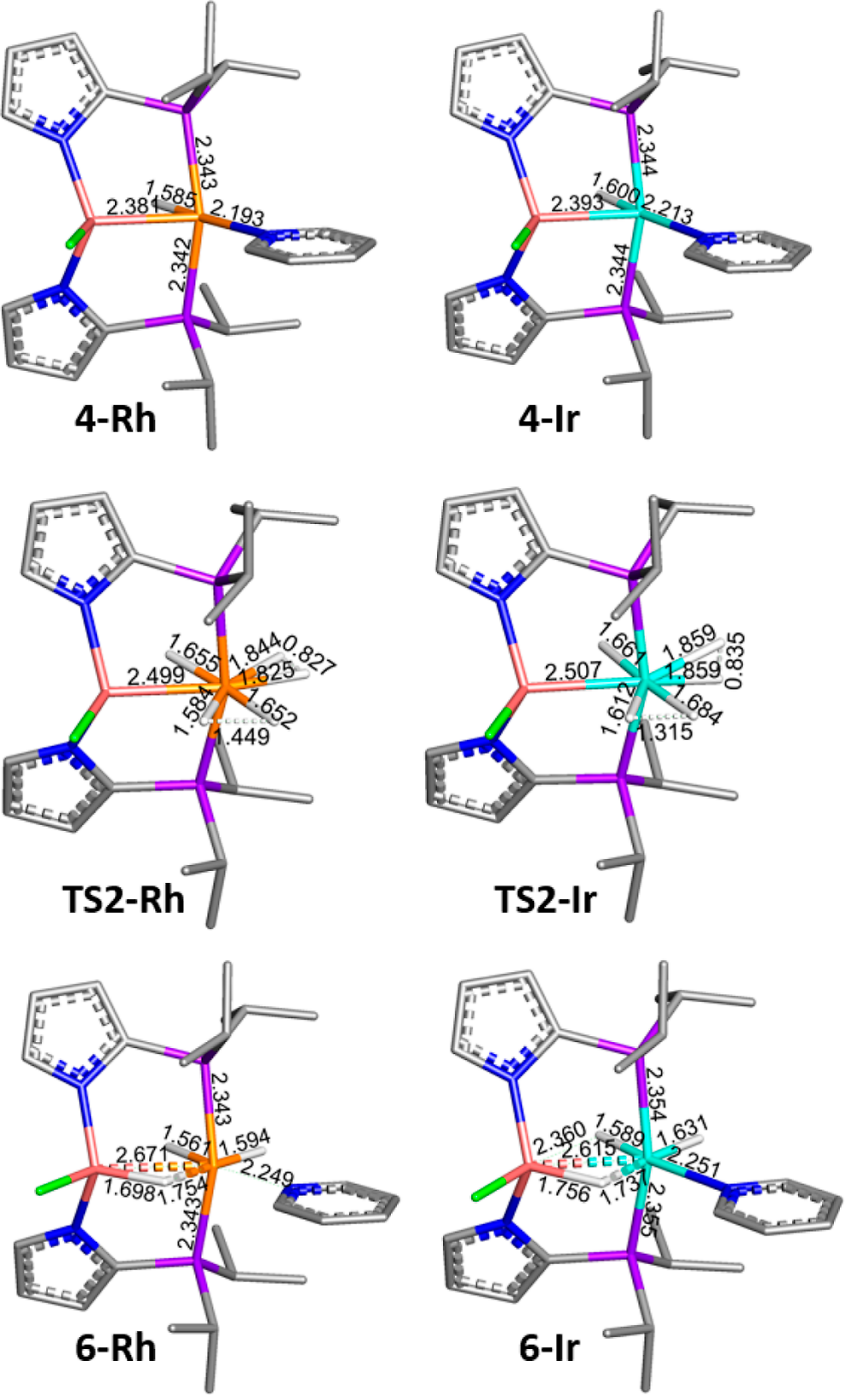
Optimized geometries for **4-Rh**, **4-Ir**, **TS2-Rh**, **TS2-Ir**, **6-Rh**, and **6-Ir**. Rh is shown in orange, Ir in cyan, P in purple, Al in brick red, N in blue, Cl in green, and H in white. Hydrogen atoms were omitted for clarity, except for those on the Rh/Ir.

We were able to identify a pathway with an overall barrier for Ir consistent with a reaction at ambient temperature (Figure 3). First, H_2_ coordination to the vacant site of **4-M** allows the formation of H_2_-complexes **INT1-M**. After the approximately ergo-neutral dissociation of pyridine (py) to give **INT2-M**, coordination of another 1 equiv of H_2_ may occur to give **INT3-M** before cleavage of the H–H bond. The energy for the transition state for the H_2_ cleavage (**TS2-M**) is considerably lower for M = Ir (19.0 kcal/mol) than for M = Rh (26.9 kcal/mol). The H_2_ activation on the Ir complex involves an earlier transition state than that on the Rh complex and the H–H distances in **TS2-Ir** and **TS2-Rh** are 1.315 and 1.449 Å, respectively (Figure 4).

Upon H_2_ cleavage, trihydride/dihydrogen complex **INT4-Ir** is initially formed (17.2 kcal/mol). It can isomerize to the more stable trihydride complex **INT5-Ir** (7.9 kcal/mol) via **TS3-Ir** (20.3 kcal/mol) through σ-complex assisted metathesis.^39^ Then, dissociation of H_2_ to give **INT6-Ir** is followed by association of pyridine, thereby forming the product **6-Ir**. **TS3-M** is the highest transition state on this path for both Rh and Ir, but its energy is considerably higher for Rh (31.0 kcal/mol). It is thus possible that the formation of **6-Rh** is prevented kinetically in addition to being thermodynamically disfavored.

It is notable that the relative energies of the Rh and Ir intermediates and transition states in Figure 3 are quite similar through **INT3-M**, following which the energies of the analogous structures for Rh are consistently 8–11 kcal/mol higher. This coincides with the presence of three hydrides in the structures, and the higher energies for Rh can be viewed as a consequence of the greater “reluctance” by Rh to undergo oxidative addition of a H–H bond. This is expected for a contrast between a 4d metal Rh and a 5d metal Ir.^40^

**Figure 5:**
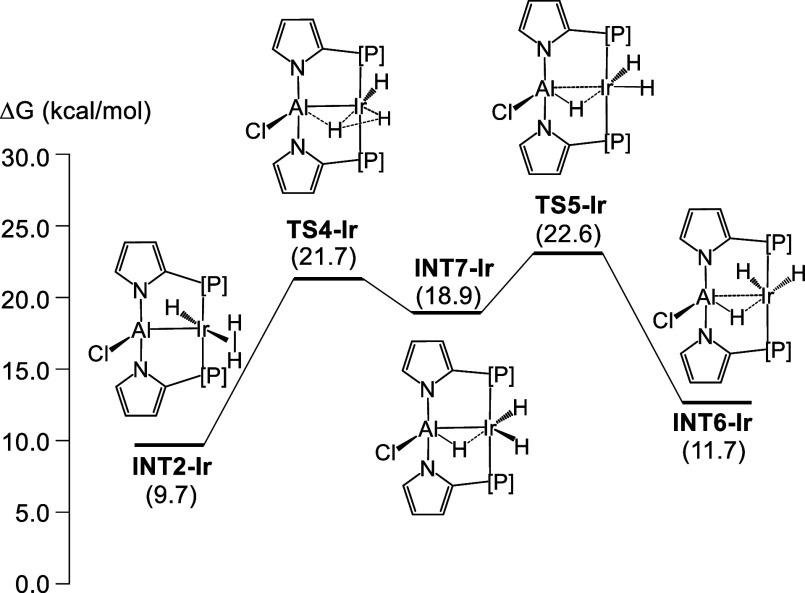
Alternative mechanism for the isomerization of **INT2-Ir** into **INT6-Ir**. Solvent-corrected relative free energies are given in parentheses (in kcal/mol, relative to **4-Ir** at zero).

The overall process is essentially a way to “flip” the Al–Cl moiety to the side of a single hydride, as is needed for **6-M**. In this vein, we considered alternative pathways (and only for the Ir system): either **INT1-Ir** needs to isomerize directly into **6-Ir** or **INT2-Ir** needs to isomerize into **INT6-Ir** without the assistance of an extra molecule of H_2_. Consideration of the direct isomerization of **INT1-Ir** into **6-Ir** produced a much higher barrier (**TS7-Ir** at 30.9 kcal/mol, see Figure S50). Direct isomerization of **INT2-Ir** into **INT6-Ir** yielded a pathway (Figure 5) that involved a higher barrier than in the H_2_-assisted pathway in Figure 3, but the difference is rather modest (**TS5-Ir** at 22.6 kcal/mol vs **TS3-Ir** at 20.3 kcal/mol). It is possible that this is a competitive pathway, especially depending on the concentration of H_2_ in solution.

The NBO^41^ analysis was performed to gain insights into the orbital interaction between the TM M (M = Rh and Ir) and Al. According to the second-order perturbation energy (Δ*E*^(2)^), substantial interaction from the occupied d-orbital, LP(M), donating to the empty p-orbital, LV(Al), was found for both **4-Rh** and **4-Ir** (Tables S7 and S8). In addition, a weak σ(M–H) donation to the empty p-orbital, LV(Al), was also found.

Upon addition of H_2_ to Ir, the calculated Ir–Al distance in **6-Ir** (2.615 Å) becomes more extended than that in **4-Ir** (2.388 Å) (Figure 4). The calculated elongation of the Ir–Al distance is greater than that found by XRD, but it is likely that the potential energy surface is quite shallow with respect to the Ir–Al distance in **6-Ir**. We found that in **6-Ir**, one of the hydrogen atoms is partly transferred from Ir to Al with a strong σ(Al–H) interaction to Ir (Δ*E*^(2)^ = 272.4 kcal mol^–1^) (Tables S7 and S8). This appears as a bridging hydrogen between Al and Ir in trihydride product **6-Ir**. The calculated structure of **6-Rh** is of the same structural type as **6-Ir**, but it possesses a shorter Al–H distance and longer Al–Rh and (Al-)H–Rh distances.

## Conclusions

In summary, we have described a series of Rh and Ir complexes supported by a PAlP ligand that combines a central alane Z-type moiety with two outer phosphine arms. The resultant complexes can be viewed as arising from an interaction of the central Z-type alane with a square-planar Rh(I) or Ir(I) fragment. The electronegative nature of the substituents on Al (*N*-pyrrolyl, chloride) leads to a strong interaction, with some of the shortest Al–Rh/Ir bonds ever recorded. DFT calculations reproduce the observed structural features well and help explain the difference between Rh and Ir in the reactions with H_2_.

## Experimental Section

### General Considerations

All manipulations were performed under an Ar atmosphere by using standard Schlenk line or glovebox techniques. Pentane, tetrahydrofuran, and toluene were dried and deoxygenated (by purging) using a solvent purification system (Innovative Technology Pure Solv MD-5 solvent purification system) and stored over molecular sieves in an Ar-filled glovebox. C_6_D_6_, CDCl_3_, and pyridine were dried over calcium hydride, vacuum transferred, and stored over molecular sieves in an Ar-filled glovebox. Celite and silica were dried at 180 °C under vacuum overnight and stored in an Ar-filled glovebox. **1**,^26^ [Ir(COE)_2_Cl]_2_,^42^ [Ir(COD)Cl]_2_,^43^ and [Rh(COD)Cl]_2_^44^ were prepared according to the literature procedures. All other chemicals were used as received from commercial vendors. Elemental analyses were performed by EuTech Scientific Services (Mount Olive, NJ) and Robertson Microlit Laboratories (Ledgewood, NJ).

### Physical Methods

NMR spectra were recorded on Varian VnmrS 500 (^1^H NMR, 500 MHz; ^13^C{^1^H} NMR, 126 MHz; ^31^P{^1^H} NMR, 202 MHz; ^27^Al{^1^H} NMR, 130 MHz), Varian Inova 500 (^1^H NMR, 500 MHz; ^13^C{^1^H} NMR, 126 MHz; ^31^P{^1^H} NMR, 202 MHz), and AVANCE NEO 400 (^1^H NMR, 400 MHz; ^13^C{^1^H} NMR, 101 MHz; ^31^P{^1^H} NMR, 162 MHz) spectrometers. Chemical shifts are reported in δ (ppm). For ^1^H and ^13^C NMR spectra, the residual solvent peak was used as an internal reference (^1^H NMR: δ 7.16 for C_6_D_6_ and 7.26 ppm for CDCl_3_; ^13^C NMR: δ 128.06 for C_6_D_6_ and 77.16 ppm for CDCl_3_). ^31^P NMR spectra were referenced externally with 85% phosphoric acid at δ 0 ppm. ^27^Al NMR spectra were referenced externally with an aqueous solution of aluminum nitrate at δ 0. Elemental analyses were performed by CALI Laboratories, Inc. (Highland Park, NJ).

### Synthesis of **2-Me**

In a J. Young NMR tube, 29 mg (0.16 mmol) of **1** was dissolved in 600 μL C_6_D_6_. A 39 μL solution of 2.0 M (0.08 mmol) AlMe_3_ in toluene was added via a microsyringe at room temperature. After 3 h at room temperature, the in situ NMR analysis determined that the clear, yellow reaction mixture was 95%+ **2-Me**. ^1^H NMR (500 MHz, C_6_D_6_, Figure S1): δ 7.33 (s, Pyrrole*H*, 2H), 6.77 (t, *J* = 3 Hz, Pyrrole*H*, 2H), 6.65–6.61 (m, Pyrrole*H*, 2H), 1.94 (octet, *J*_H,P_ ≈ *J*_H,H_ = 7 Hz, C*H*(Me)_2_, 4H), 0.94 (dd, *J*_H,P_ = 13, *J*_H,H_ = 7 Hz, CH(*Me*)_2_, 12H), 0.93 (dd, *J*_H,P_ = 17, *J*_H,H_ = 7 Hz, CH(*Me*)_2_, 12H), 0.05 (t, *J* = 3 Hz, Al–*Me*, 3H). ^13^C{^1^H} NMR (126 MHz, C_6_D_6_, Figure S2): δ 125.2 (s, *C*_Pyrrole_), 124.9 (s, *C*_Pyrrole_), 116.0 (s, *C*_Pyrrole_), 114.3 (s, *C*_Pyrrole_), 23.7 (m, C*H*(Me)_2_), 19.4 (m, C*H*(Me)_2_), 18.9 (s, CH(*Me*)_2_), −3.8 (br s, Al–*Me*). ^31^P{^1^H} NMR (202 MHz, C_6_D_6_, Figure S3): δ −3.1 (s). ^27^Al{^1^H} NMR (130 MHz, C_6_D_6_, Figure S4): δ 146.6.

### Synthesis of **2-**^***i***^**Bu** and **2-H**

Into a J. Young NMR tube, 400 μL of a 250 mM solution of **1** in C_6_D_6_ was introduced. Al(^*i*^Bu)_3_ was then added as 50 μL of a 1 M solution in hexanes. After the mixture was heated for 1.5 h at 80 °C, an in situ NMR analysis determined the reaction mixture as 79% **2-**^***i***^**Bu**, 16% **2-H**, and 5% unknown impurities. Further thermolysis did not significantly change this ratio. **2**-^***i***^**Bu**. ^1^H NMR (400 MHz, C_6_D_6_, Figure S5): δ 7.30 (s, Pyrrole*H*, 2H), 6.74 (t, *J* = 3 Hz, Pyrrole*H*, 2H), 6.59 (m, Pyrrole*H*, 2H), 1.98 (m, C*H*(Me)_2_, 4H), 0.64 (dt, *J* = 3, 7 Hz, Al–C*H*_2_CH_3_, 2H). ^31^P{^1^H} NMR (162 MHz, C_6_D_6_, Figure S6): δ −3.8 (s). **2**-**H**. ^1^H NMR (400 MHz, C_6_D_6_, Figure S5): δ 7.48 (s, Pyrrole*H*, 2H), 6.70 (t, *J*_H,H_ = 3 Hz, Pyrrole*H*, 2H), 6.56 (d, *J*_H,H_ = 3 Hz, Pyrrole*H*, 2H). ^31^P{^1^H} NMR (162 MHz, C_6_D_6_, Figure S6): δ −4.1 (s).

### Synthesis of **3-Rh**

In a 50 mL Schlenk flask, 366 mg (2.00 mmol) of **1** was dissolved in 10 mL of toluene. A 500 μL of 2.0 M (1.00 mmol) AlMe_3_ solution in toluene was added via a microsyringe at room temperature. After stirring at room temperature for 3 h, 89 μL (1.10 mmol) of pyridine was added. Then, a solution of 247 mg (0.50 mmol) of [Rh(COD)Cl]_2_ in 10 mL of toluene was added. The reaction mixture was stirred for 2 h, during which a yellow precipitate formed. The flask was cooled in a −35 °C freezer before the precipitate was collected via filtration. The precipitate was rinsed with 6 mL (2 mL × 3) of cold toluene and dried under vacuum to yield 497 mg of **3-Rh** (80%). Crystals for X-ray analysis were obtained via recrystallization from THF. ^1^H NMR (500 MHz, C_6_D_6_, Figure S7): δ 9.57 (br s, Py*H*, 1H), 8.52 (br s, Py*H*, 1H), 7.61 (m, Pyrrole*H*, 2H), 6.78 (m, Pyrrole*H*, 4H), 6.63 (m, Py*H*, 1H), 6.37 (br s, Py*H*, 2H), 2.41 (m, C*H*(Me)_2_, 2H), 2.09 (m, C*H*(*Me*)_2_, 2H), 1.31 (dvt, CH(*Me*)_2_, *J*_H,P_ ≈ *J*_H,H_ = 8 Hz, 6H), 1.23 (dvt, CH(*Me*)_2_, *J*_H,P_ ≈ *J*_H,H_ = 7 Hz, 6H), 0.89 (dvt, CH(*Me*)_2_, *J*_H,P_ ≈ *J*_H,H_ = 7 Hz, 6H), 0.34 (dvt, CH(*Me*)_2_, *J*_H,P_ ≈ *J*_H,H_ = 7 Hz, 6H), 0.29 (td, *J*_H,Rh_ = 2 Hz, *J*_H,P_ = 7 Hz, Rh–*Me*, 3H). ^13^C{^1^H} NMR (126 MHz, C_6_D_6_, Figure S8): δ 156.0 (br s, *C*_Py_), 148.5 (br s, *C*_Py_), 136.3 (s, *C*_Py_), 130.4 (vt, *J*_C,P_ = 32.9 Hz, *C*_Pyrrole_), 128.4 (m, overlaps with C_6_D_6_, *C*_Pyrrole_), 125.6 (br s, *C*_Py_), 123.6 (br s, *C*_Py_), 115.7 (vt, *J*_C,P_ = 4 Hz, *C*_Pyrrole_), 112.6 (vt, *J*_C,P_ = 3 Hz, *C*_Pyrrole_), 28.5 (vt, *J*_C,P_ = 13 Hz, *C*H(Me)_2_), 26.4 (vt, *J*_C,P_ = 12 Hz, *C*H(Me)_2_), 22.8 (vt, *J*_C,P_ = 5 Hz, CH(*Me*)_2_), 20.0 (s, CH(*Me*)_2_), 18.8 (s, CH(*Me*)_2_), 18.7 (m, CH(*Me*)_2_), −3.7 (d, *J*_C,Rh_ = 22 Hz, Rh–*Me*). ^31^P{^1^H} NMR (202 MHz, C_6_D_6_, Figure S9): δ 33.7 (d, *J*_Rh,P_ = 120 Hz). ^27^Al{^1^H} NMR (130 MHz, C_6_D_6_, Figure S10): δ 127.6. Anal. Calcd for C_26_H_42_N_3_AlRhP_2_Cl: C, 50.05; H, 6.79; N, 6.73. Found: C, 50.08; H, 6.81; N, 5.79.

### Synthesis of **3-Ir**

In a 50 mL Schlenk flask, 670 mg (3.66 mmol) of **1** was loaded with 10 mL of toluene. 915 μL of 2.0 M (1.83 mmol) AlMe_3_ solution in toluene was added to the above solution via microsyringe at room temperature. The resulting clear solution was stirred at room temperature for 3 h before 158 mg (2.01 mmol) of pyridine was added. 819 mg (0.92 mmol) of [Ir(COE)_2_Cl]_2_ in 10 mL of toluene was then added to the resulting mixture. The yellow precipitate forms immediately upon mixing. The suspension was stirred at room temperature for 2 h before filtration. The precipitate was washed with 4.5 mL (1.5 mL × 3) of cold toluene to afford a total of 910 mg of **3-Ir** (70%) as a yellow powder. Single crystals for X-ray analysis were obtained via recrystallization from a concentrated solution in THF. ^1^H NMR (400 MHz, CDCl_3_, Figure S11): δ 9.65 (d, *J*_H,H_ = 4, Py*H*, 1H), 8.94 (d, *J*_H,H_ = 4, Py*H*, 1H), 7.73 (t, *J*_H,H_ = 8, Py*H*, 1H), 7.38 (m, Py*H*, 2H), 7.26 (m, overlaps with solvent, Pyrrole*H*, 2H), 6.59 (d, *J*_H,H_ = 3, Pyrrole*H*, 2H), 6.44 (m, Pyrrole*H*, 2H), 2.74 (m, C*H*(CH_3_)_2_, 2H), 2.50 (m, C*H*(CH_3_)_2_, 2H), 1.49 (m, CH(C*H*_3_)_2_, 12H), 0.79 (dvt, *J*_H,P_ ≈ *J*_H,H_ = 7 Hz, CH(C*H*_3_)_2_, 6H), 0.54 (m, CH(C*H*_3_)_2_ and Ir–C*H*_3_, 9H). ^1^H NMR (500 MHz, C_6_D_6_, Figure S12): δ 9.81 (s, Py*H*, 1H), 8.48 (s, Py*H*, 1H), 7.63 (m, Pyrrole*H*, 2H), 6.78 (m, Py*H*, 4H), 6.58 (m, Py*H*, 1H), 6.32 (m, Py*H*, 1H), 6.29 (m, Py*H*, 1H), 2.61 (m, C*H*(CH_3_)_2_, 2H), 2.35 (m, C*H*(CH_3_)_2_, 2H), 1.29 (m, CH(C*H*_3_)_2_, 6H), 1.24 (m, CH(C*H*_3_)_2_, 6H), 0.86 (m, CH(C*H*_3_)_2_ and Ir–C*H*_3_, 9H), 0.37 (m, CH(C*H*_3_)_2_, 6H). ^13^C{^1^H} NMR (126 MHz, C_6_D_6_, Figure S13): δ 156.5 (s, *C*_Py_), 147.7 (s, *C*_Py_), 135.4 (s, *C*_Py_), 130.0 (vt, *J*_C,P_ = 40 Hz), 126.2 (s, *C*_Py_), 123.7 (s, *C*_Py_), 115.5 (vt, *J*_C,P_ = 5 Hz, *C*_Pyrrole_), 112.3 (vt, *J*_C,P_ = 3 Hz, *C*_Pyrrole_), 28.8 (vt, *J*_C,P_ = 16 Hz, *C*H(Me)_2_), 25.9 (vt, *J*_C,P_ = 15 Hz, *C*H(Me)_2_), 22.6 (vt, *J*_C,P_ = 4 Hz, CH(*Me*)_2_), 19.6 (s, CH(*Me*)_2_), 19.1 (s, CH(*Me*)_2_), 18.7 (m, CH(*Me*)_2_), −18.4 (t, *J*_C,P_ = 6 Hz, Ir–*Me*). ^31^P{^1^H} NMR (202 MHz, C_6_D_6_, Figure S14): δ 31.1 (s). ^27^Al{^1^H} NMR (130 MHz, C_6_D_6_, Figure S15): δ 96.8. Elem. Anal. Calcd for C_26_H_42_N_3_AlIrP_2_Cl: C, 43.78; H, 5.94; N, 5.89. Found: C, 43.40; H, 5.58; N, 5.64.

### Synthesis of **4-Rh**

Method A: to a 10 mL Teflon screw-cap Schlenk flask, 123 mg (0.20 mmol) of **3-Rh** was loaded with 10 mL of toluene. The flask was degassed via freeze–pump–thaw cycles and then backfilled with 1 atm H_2_. The resulting solution was stirred at room temperature for 3 h before the volatiles were removed. The residual yellow powder was rinsed with pentane and dried to yield 106 mg **4-Rh** (88%). Yellow crystals were obtained by heating a suspension of **4** (0.10 mmol) in 5 mL of toluene to 90 °C and then cooling to room temperature.

Method B: to a J. Young NMR tube, **3-Rh** (0.01 mmol) was suspended in C_6_D_6_. The sample was degassed via freeze–pump–thaw cycles and then backfilled with 1 atm H_2_. The in situ NMR analysis indicated the formation of 90% **4-Rh**. See Figures S16 and S17.

Method C: a 400 μL solution (0.1 mmol) of 250 mM **1** in C_6_D_6_ and a 50 μL solution of 1.0 M Al^*i*^Bu_3_ in hexanes were added to a J. Young NMR tube. The solution was heated at 80 °C for 1.5 h before 12.3 mg (25 μmol) of [Rh(COD)Cl]_2_ was introduced. The reaction mixture was heated at 50 °C for 1 h. Storage overnight at −35 °C precipitated light-yellow solids. Solids were rinsed with pentane and vacuum-dried to yield **4-Rh** (20 mg, 67%). ^1^H NMR (500 MHz, C_6_D_6_, Figure S18): δ 8.81 (d, *J*_HH_ = 5.4 Hz, Py*H*, 2H), 7.57 (dd, *J*_HH_ = 2, 1 Hz, Pyrrole*H*, 2H), 6.81 (t, *J*_HH_ = 3 Hz, Pyrrole*H*, 2H), 6.70 (m, Py*H*, 1H), 6.65 (dd, *J*_HH_ = 3, 1 Hz, Pyrrole*H*, 2H), 6.46 (m, Py*H*, 2H), 2.24–2.13 (m, C*H*(CH_3_)_2_, 2H), 1.60 (m, C*H*(CH_3_)_2_, 2H), 1.16–1.08 (m, CH(C*H*_3_)_2_, 6H), 1.11–1.03 (m, CH(C*H*_3_)_2_, 6H), 0.94 (dvt, *J*_HP_ ≈ J_HH_ = 7 Hz, CH(C*H*_3_)_2_, 6H), 0.78 (dvt, *J*_HP_ ≈ J_HH_ = 8 Hz, CH(C*H*_3_)_2_, 6H), −17.96 (q, *J*_HP_ ≈ *J*_H,Rh_ = 20 Hz, Rh–*H*, 1H). ^13^C{^1^H} NMR (126 MHz, C_6_D_6_, Figure S19): δ 151.5 (s, *C*_Py_), 136.1 (s, *C*_Py_), 128.7 (vt, *J*_C,P_ = 7 Hz, *C*_Pyrrole_), 124.5 (s), 114.9 (vt, *J*_C,P_ = 4 Hz, *C*_Pyrrole_), 113.4 (vt, *J*_C,P_ = 3 Hz, *C*_Pyrrole_), 28.2 (vt, *J*_C,P_ = 15 Hz, *C*H(Me)_2_), 27.0 (vt, *J*_C,P_ = 12 Hz, *C*H(Me)_2_), 21.4 (s, CH(*Me*)_2_), 20.5 (vt, *J*_C,P_ = 4 Hz, CH(*Me*)_2_), 19.4 (vt, *J*_C,P_ = 3 Hz, CH(*Me*)_2_), 18.1 (s, CH(*Me*)_2_). ^31^P{^1^H} NMR (162 MHz, C_6_D_6_, Figure S20): δ 34.2 (d, *J*_PRh_ = 121 Hz). ^27^Al{^1^H} NMR (130 MHz, C_6_D_6_, Figure S21): δ 125.1.

### Synthesis of **4-Ir**

In a 20 mL vial, 92 mg (0.50 mmol) of **1** was dissolved in 2 mL of toluene and 250 μL of 1.0 M Al^*i*^Bu_3_ in hexanes was added. The solution was heated at 80 °C for 1.5 h before 21 μL pyridine was introduced. Addition of 84 mg (0.13 mmol) of [Ir(COD)Cl]_2_ turned the solution from yellow to dark red. The solution was heated at 100 °C for 2 h and allowed to cool to room temperature, resulting in the formation of bright yellow precipitates. The precipitates were rinsed with cold pentane and dried under vacuum to yield 114 mg of **4-Ir** (66%). ^1^H NMR (500 MHz, CDCl_3_, Figure S22): δ 8.98 (d, *J*_H,H_ = 6 Hz, Py*H*, 2H), 7.68 (t, *J*_H,H_ = 8 Hz, Py*H*, 1H), 7.40 (m, Pyrrole*H*, 2H), 7.25 (m, overlaps with solvent peaks, Py*H*), 6.47 (m, Pyrrole*H*, 4H), 2.77 (m, C*H*(CH_3_)_2_, 2H), 1.92 (m, C*H*(CH_3_)_2_, 2H), 1.25 (dvt, *J*_H,P_ ≈ *J*_H,H_ = 8 Hz, CH(C*H*_3_)_2_, 6H), 1.16 (dvt, *J*_H,P_ ≈ *J*_H,H_ = 7 Hz, CH(C*H*_3_)_2_, 6H), 1.00 (dvt, *J*_H,P_ ≈ *J*_H,H_ = 7 Hz, CH(C*H*_3_)_2_, 6H), 0.73 (dvt, *J*_H,P_ ≈ *J*_H,H_ = 8 Hz, CH(C*H*_3_)_2_, 6H), −20.60 (t, *J*_H,P_ = 17.1 Hz, Ir–*H*). ^1^H NMR (400 MHz, C_6_D_6_, Figure S23): δ 8.83 (s, Py*H*, 2H), 7.61 (s, Pyrrole*H*, 2H), 6.85 (s, Pyrrole*H*, 2H), 6.64 (d, *J*_H,H_ = 3 Hz, Pyrrole*H*, 2H), 6.58 (t, *J*_H,H_ = 8 Hz, Py*H*, 1H), 6.37 (t, *J*_H,H_ = 7 Hz, Py*H*, 2H), 2.49 (m, C*H*(CH_3_)_2_, 2H), 1.73 (m, C*H*(CH_3_)_2_, 2H), 1.14 (dvt, *J*_H,P_ ≈ *J*_H,H_ = 8 Hz, CH(C*H*_3_)_2_, 6H), 1.01 (dvt, *J*_H,P_ ≈ *J*_H,H_ = 8 Hz, CH(C*H*_3_)_2_, 6H), 0.92 (dvt, *J*_H,P_ ≈ *J*_H,H_ = 7 Hz, CH(C*H*_3_)_2_, 6H), 0.78 (dvt, *J*_H,P_ ≈ *J*_H,H_ = 8 Hz, CH(C*H*_3_)_2_, 6H), −20.30 (t, *J*_H,P_ = 17.2 Hz, Ir–*H*). ^13^C{^1^H} NMR (101 MHz, CDCl_3_, Figure S24): δ 150.9 (s, *C*_Py_), 136.5 (s, *C*_Py_), 130.0 (vt, *J*_C,P_ = 40 Hz, *C*_Pyrrole_), 127.2 (vt, *J*_C,P_ = 8 Hz, *C*_Pyrrole_), 125.6 (s, *C*_Py_), 113.9 (vt, *J*_C,P_ = 6 Hz, *C*_Pyrrole_), 112.0 (vt, *J*_C,P_ = 4 Hz, *C*_Pyrrole_), 28.2 (vt, *J*_C,P_ = 22 Hz, *C*H(Me)_2_), 27.0 (vt, *J*_C,P_ = 18 Hz, *C*H(Me)_2_), 21.3 (s, CH(*Me*)_2_), 20.3 (vt, *J*_C,P_ = 4 Hz, CH(*Me*)_2_), 19.0 (vt, *J*_C,P_ = 1 Hz, CH(*Me*)_2_), 18.1 (s, CH(*Me*)_2_). ^31^P{^1^H} NMR (202 MHz, CDCl_3_, Figure S25): δ 37.1 (s). ^31^P{^1^H} NMR (202 MHz, C_6_D_6_, Figure S26): δ 36.3 (s). ^27^Al{^1^H} NMR (130 MHz, CDCl_3_, Figure S27): δ 95.9.

### Synthesis of **5-Rh**

In a 50 mL culture tube, 312 mg (0.50 mmol) of **3-Rh** was dissolved in 15 mL toluene. A 500 μL solution of 1.0 M (0.50 mmol) NaHBEt_3_ in toluene was added to the mixture via a microsyringe. The resulting mixture was then heated in an 80 °C oil bath for 4 h before being filtered through a short pad of Celite. All volatiles were removed under vacuum. The remaining residue was recrystallized from THF layered with pentane at −35 °C to obtain **5-Rh** as orange–yellow crystals, 156 mg (50%) of **5-Rh**. ^1^H NMR (400 MHz, C_6_D_6_, Figure S28): δ 8.61 (d, *J*_H,H_ = 5 Hz, Py*H*, 2H), 7.55 (s, Pyrrole*H*, 2H), 6.88 (s, 2H), 6.68 (d, *J*_H,H_ = 3 Hz, Pyrrole*H*, 2H), 6.66 (t, *J*_H,H_ = 8 Hz, Py*H*, 1H), 6.39 (t, *J*_H,H_ = 7 Hz, Py*H*, 2H), 2.20 (m, C*H*(Me)_2_, 2H), 1.63 (m, C*H*(Me)_2_, 2H), 1.18 (dvt, *J*_H,P_ ≈ *J*_H,H_ = 7 Hz, CH(*Me*)_2_, 6H), 0.94 (dvt, *J*_H,P_ ≈ *J*_H,H_ = 7 Hz, CH(*Me*)_2_, 6H), 0.70 (dvt, *J*_H,P_ ≈ *J*_H,H_ = 7 Hz, CH(*Me*)_2_, 6H), 0.19 (s, Al–*Me*, 3H), −17.85 (dt, *J*_H,Rh_ = 22.4 Hz, *J*_H,P_ = 19.3 Hz, Rh–*H*, 1H). ^1^H NMR (500 MHz, CDCl_3_, Figure S29): δ 8.83 (d, *J*_H,H_ = 5 Hz, Py*H*, 2H), 7.70 (t, *J*_H,H_ = 8 Hz, Py*H*, 1H), 7.37 (t, *J*_H,H_ = 7 Hz, Py*H*, 2H), 7.20 (s, Pyrrole*H*, 2H), 6.45 (m, Pyrrole*H*, 2H), 6.41 (m, Pyrrole*H*, 2H), 2.39 (m, C*H*(Me)_2_, 2H), 1.78 (m, C*H*(Me)_2_, 2H), 1.29 (dvt, *J*_H,P_ ≈ *J*_H,H_ = 8 Hz, CH(*Me*)_2_, 6H), 1.13 (dvt, *J*_H,P_ ≈ *J*_H,H_ = 8 Hz, CH(*Me*)_2_, 6H), 0.97 (dvt, *J*_H,P_ ≈ *J*_H,H_ = 7 Hz, CH(*Me*)_2_, 6H), 0.70 (dvt, *J*_H,P_ ≈ *J*_H,H_ = 8 Hz, CH(*Me*)_2_, 6H), −0.39 (s, Al–*Me*, 3H), −18.17 (dt, *J*_H,Rh_ = 23.8 Hz, *J*_H,P_ = 18.7 Hz, Rh–*H*, 1H). ^13^C{^1^H} NMR (126 MHz, CDCl_3_, Figure S30): δ 151.6 (s, *C*_Py_), 136.5 (s, *C*_Py_), 129.8 (vt, *J*_C,P_ = 35 Hz, *C*_Pyrrole_), 127.1 (vt, *J*_C,P_ = 7 Hz, *C*_Pyrrole_), 125.0 (s, *C*_Py_), 113.3 (vt, *J*_C,P_ = 4 Hz, *C*_Pyrrole_), 111.5 (vt, *J*_C,P_ = 3 Hz, *C*_Pyrrole_), 28.3 (vt, *J*_C,P_ = 15 Hz, *C*H(Me)_2_), 26.7 (vt, *J*_C,P_ = 12 Hz, *C*H(Me)_2_), 21.3 (s, CH(*Me*)_2_), 20.5 (vt, *J*_C,P_ = 4 Hz, CH(*Me*)_2_), 19.4 (vt, *J*_C,P_ = 2 Hz, CH(*Me*)_2_), 18.1 (s, CH(*Me*)_2_), −4.9 (br s, Al–*Me*). ^31^P{^1^H} NMR (162 MHz, CDCl_3_, Figure S31): δ 35.1 (d, *J*_P,Rh_, = 125 Hz). ^27^Al{^1^H} NMR (130 MHz, CDCl_3_, Figure S32): δ 155.9.

### Thermolysis of **5-Rh**

In a J. Young NMR tube, **5-Rh** (0.01 mmol) was suspended in 600 μL C_6_D_6_. The sample was heated at 100 °C and analyzed via ^1^H and ^31^P{^1^H} NMR spectroscopy at the 1, 7, 17, and 37 h time points. See Figures S40, S41, and S42.

### Synthesis of **6-Ir**

Method A: to a 50 mL pressure flask, 142 mg (0.20 mmol) of **3-Ir** was suspended in 10 mL of toluene. The flask was degassed via freeze–pump–thaw cycles and then backfilled with 1 atm H_2_. The suspension was heated to 90 °C for 30 min. The resulting pale, yellow solution was slowly cooled to −35 °C to obtain **6-Ir** as white crystals. These crystals were collected and rinsed with 4.5 mL (1.5 mL × 3) of cold pentane to yield 101 mg of **7** (72%).

### Method B

In a J Young NMR tube, 35 mg (0.05 mmol) of **4-Ir** was dissolved in 600 μL of CDCl_3_. The sample was degassed via freeze–pump–thaw cycles and then backfilled with 1 atm H_2_. The solution was inverted to mix for 3 h. The in situ NMR analysis indicated the >99% formation of **6-Ir** (Figures S33 and S34).

The solution was dried under vacuum, and the resulting precipitates were rinsed with pentane and further dried under vacuum to yield 32 mg of **6-Ir** (95%). ^1^H NMR (500 MHz, CDCl_3_, Figure S35): δ 9.15 (d, *J*_H,H_ = 5 Hz, Py*H*, 2H), 7.75 (t, *J*_H,H_ = 8 Hz, Py*H*, 1H), 7.29 (s, Pyrrole*H*, 2H), 7.18 (t, *J*_H,H_ = 7 Hz, Py*H*, 2H), 6.57 (s, Pyrrole*H*, 2H), 6.47 (d, *J*_H,H_ = 3 Hz, Pyrrole*H*, 2H), 2.47 (m, C*H*(CH_3_)_2_, 2H), 1.54 (m, C*H*(CH_3_)_2_, 2H), 1.14 (m, CH(C*H*_3_)_2_, 12H), 0.91 (m, CH(C*H*_3_)_2_, 12H), −6.12 (br s, Ir–*H*–Al), −9.97 (qd, *J*_H,P_ ≈ *J*_H,H_ = 14 Hz, *J*_H,H_ = 4 Hz, Ir–*H*, 1H), −22.18 (td, *J*_H,H_ = 4 Hz, *J*_H,P_ = 15 Hz, Ir–*H*, 1H). ^1^H NMR (400 MHz, C_6_D_6_, Figure S36): δ 8.75 (d, *J*_H,H_ = 5 Hz, Py*H*, 2H), 7.72 (s, Py*H*, 1H), 6.91 (s, Pyrrole*H*, 2H), 6.62 (d, *J*_H,H_ = 3 Hz, Pyrrole*H*, 2H), 6.56 (t, *J*_H,H_ = 8 Hz, Py*H*, 1H), 6.08 (t, *J*_H,H_ = 7 Hz, 2*H*, 2H), 2.23 (m, C*H*(CH_3_)_2_, 2H), 1.29 (m, C*H*(CH_3_)_2_, 2H), 0.94(m, C*H*(CH_3_)_2_, 24H), −5.96 (br s, Ir–*H*–Al), −9.97 (q, *J*_H,P_ ≈ *J*_H,H_ = 14 Hz, Ir–*H*, 1H), −21.95 (t, *J*_H,P_ = 14 Hz, Ir–*H*, 1H). ^13^C{^1^H} NMR (126 MHz, CDCl_3_, Figure S37): δ 159.6 (s, *C*_Py_), 136.2 (s, *C*_Py_), 129.0 (vt, *J*_C,P_ = 6 Hz, *C*_Pyrrole_), 128.1 (vt, *J*_C,P_ = 39 Hz, *C*_Pyrrole_), 126.4 (s, *C*_Py_), 114.5 (vt, *J*_C,P_ = 5 Hz, *C*_Pyrrole_), 112.8 (vt, *J*_C,P_ = 3 Hz, *C*_Pyrrole_), 25.13 (vt, *J*_C,P_ = 20 Hz, *C*H(Me)_2_), 24.7 (vt, *J*_C,P_ = 15 Hz, *C*H(Me)_2_), 19.4 (s, CH(*Me*)_2_), 19.3 (vt, *J*_C,P_ = 2 Hz, CH(*Me*)_2_), 18.0 (vt, *J*_C,P_ = 3 Hz, CH(*Me*)_2_), 16.0 (s, CH(*Me*)_2_). ^31^P{^1^H} NMR (202 MHz, CDCl_3_, Figure S38): δ 19.9 (s). ^27^Al{^1^H} NMR (130 MHz, CDCl_3_, Figure S39): δ 120.9.

## Supporting Information Available

Experimental details and pictorial NMR spectra (PDF)
DFT computational studies (ZIP)

## References

[ref1] Sircoglou M.; Saffon N.; Miqueu K.; Bouhadir G.; Bourissou D. Activation of M–Cl Bonds with Phosphine–Alanes: Preparation and Characterization of Zwitterionic Gold and Copper Complexes. Organometallics 2013, 32, 6780–6784. 10.1021/om4005884.

[ref2] Green M. L. H.; Parkin G. Application of the Covalent Bond Classification Method for the Teaching of Inorganic Chemistry. J. Chem. Educ. 2014, 91, 807–816. 10.1021/ed400504f.

[ref3] Takaya J.; Iwasawa N. Synthesis, Structure, and Catalysis of a Palladium Complexes Bearing a Group 13 Metalloligand: Remarkable Effect of an aluminum-Metalloligand in Hydrosilylation of CO_2_. J. Am. Chem. Soc. 2017, 139, 6074–6077. 10.1021/jacs.7b02553. 28423896

[ref4] Hara N.; Saito T.; Semba K.; Kuriakose N.; Zheng H.; Sakaki S.; Nakao Y. Rhodium Complexes Bearing PAlP Pincer Ligands. J. Am. Chem. Soc. 2018, 140, 7070–7073. 10.1021/jacs.8b04199. 29792688

[ref5] Semba K.; Fujii I.; Nakao Y. A PAlP Pincer Ligand Bearing a 2-Diphenylphosphinophenoxy Backbone. Inorganics 2019, 7, 140 10.3390/inorganics7120140.

[ref6] Lai Q.; Bhuvanesh N.; Ozerov O. V. Unexpected B/Al Transelementation Within a Rh Pincer Complex. J. Am. Chem. Soc. 2020, 142, 20920–20923. 10.1021/jacs.0c09344. 33263407

[ref7] Morisako S.; Watanabe S.; Ikemoto S.; Muratsugu S.; Tada M.; Yamashita M. Synthesis of a Pincer-Ir^V^ Complex with a Base-Free Alumanyl Ligand and Its Application toward the Dehydrogenation of Alkanes. Angew. Chem. 2019, 131, 15173–15177. 10.1002/ange.201909009. 31397531

[ref8] Graziano B. J.; Vollmer M. V.; Lu C. C. Cooperative Bond Activation and Facile Intramolecular Aryl Transfer of Nickel–Aluminum Pincer-type Complexes. Angew. Chem., Int. Ed. Engl. 2021, 60, 15087–15094. 10.1002/anie.202104050. 33871130

[ref9] Hara N.; Semba K.; Nakao Y. X-type Aluminyl Ligands for Transition-Metal Catalysis. ACS Catal. 2022, 12, 1626–1638. 10.1021/acscatal.1c04340.

[ref10] aRudd P. A.; Liu S.; Gagliardi L.; Young V. G.; Lu C. C. Metal-Alane Adducts with Zero-Valent Nickel, Cobalt, and Iron. J. Am. Chem. Soc. 2011, 133, 20724–20727. 10.1021/ja2099744. 22122804

[ref11] aMears K. L.; Stennett C. R.; Taskinen E. K.; Knapp C. E.; Carmalt C. J.; Tuononen H. M.; Power P. P. Molecular Complexes Featuring Unsupported Dispersion-Enhanced Aluminum-Copper and Gallium-Copper Bonds,. J. Am. Chem. Soc. 2020, 142, 19874–19878. 10.1021/jacs.0c10099. 33170691

[ref12] aKuriakose N.; Zheng J.-J.; Saito T.; Hara N.; Nakao Y.; Sakaki S. Characterization of Rh-Al Bond in Rh(PAlP) in Rh(PAlP) (PAlP= Pincer-type Diphosphino-Aluminyl Ligand) in Comparison with Rh(L)(PMe_3_)_2_ (L= AlMe_2_, Al(NMe_2_)_2_, BR_2_, SiR_3_, CH_3_, Cl, or OCH_3_): Theoretical Insight. Inorg. Chem. 2019, 58, 4894–4906. 10.1021/acs.inorgchem.8b03493. 30946577

[ref13] Escomel L.; Del Rosal I.; Maron L.; Jeanneau E.; Veyre L.; Thieuleux C.; Camp C. Strongly Polarized Iridium^δ−^-Aluminum^δ+^ Pairs: Unconventional Reactivity Patterns Including CO_2_ Cooperative Reductive Cleavage. J. Am. Chem. Soc. 2021, 143, 4844–4856. 10.1021/jacs.1c01725. 33735575

[ref14] Shih W.-C.; Gu W.; MacInnis M. C.; Timpa S.; Bhuvanesh N.; Zhou J.; Ozerov O. V. Facile Insertion of Rh and Ir into a Boron–Phenyl Bond, Leading to Boryl/Bis(phosphine) PBP Pincer Complexes. J. Am. Chem. Soc. 2016, 138, 2086–2089. 10.1021/jacs.5b11706. 26824249

[ref15] Amgoune A.; Bourissou D. σ-Acceptor, Z-type Ligands for TMs. Chem. Commun. 2011, 47, 859–871. 10.1039/C0CC04109B. 21103473

[ref16] aHara N.; Yamamoto K.; Tanaka Y.; Saito T.; Sakaki S.; Nakao Y. Synthesis, Electronic Properties, and Lewis Acidity of Rhodium Complexes Bearing X-Type PBP, PAlP, and PGaP Pincer Ligands. Bull. Chem. Soc. Jpn. 2021, 94, 1859–1868. 10.1246/bcsj.20210068.

[ref17] Fujii I.; Semba K.; Li Q.-Z.; Sakaki S.; Nakao Y. Magnesiation of Aryl Fluorides Catalyzed by a Rhodium-Aluminum Complex. J. Am. Chem. Soc. 2020, 142, 11647–11652. 10.1021/jacs.0c04905. 32515952

[ref18] Fajardo J.; Peters J. C. Tripodal P_3_^X^Fe-N_2_ Complexes (X= B, Al, Ga): Effect of the Apical Atom on Bonding, Electronic Structure, and Catalytic N_2_-to-NH_3_ Conversion. Inorg. Chem. 2021, 60, 1220–1227. 10.1021/acs.inorgchem.0c03354. 33410667 PMC8279418

[ref19] Shih W.-C.; Gu W.; MacInnis M. C.; Herbert D. E.; Ozerov O. V. Boryl/Borane Interconversion and Diversity of Binding Modes of Oxygenous Ligands in PBP Pincer Complexes of Rhodium,. Organometallics 2017, 36, 1718–1726. 10.1021/acs.organomet.7b00070.

[ref20] Shih W.-C.; Ozerov O. V. Synthesis and Characterization of PBP Pincer Iridium Complexes and Their Application in Alkane Transfer Dehydrogenation,. Organometallics 2017, 36, 228–233. 10.1021/acs.organomet.6b00762.

[ref21] Cao Y.; Shih W.-C.; Ozerov O. V. Addition of O-H, N-H and F-H Bonds across a Boryl-Iridium Unit. Organometallics 2019, 38, 4076–4081. 10.1021/acs.organomet.8b00785.

[ref22] Cao Y.; Shih W.-C.; Bhuvanesh N. S.; Ozerov O. V. Reversible Addition of Ethylene to a Pincer-Based Boryl-Iridium Unit with the Formation of a Bridging Ethylidene. Chem. Sci. 2020, 11, 10998–11002. 10.1039/D0SC04748A. 34094348 PMC8162418

[ref23] Shih W.-C.; Ozerov O. V. Selective ortho C-H Activation of Pyridines Directed by Lewis Acidic Boron of PBP Pincer Iridium Complexes,. J. Am. Chem. Soc. 2017, 139, 17297–17300. 10.1021/jacs.7b10570. 29112403

[ref24] Cao Y.; Shih W.-C.; Bhuvanesh N.; Zhou J.; Ozerov O. V. Cooperative C-H activation of pyridine by PBP complexes of Rh and Ir can lead to bridging 2-pyridyls with different connectivity to the B-M unit. Chem. Sci. 2021, 12, 14167–14173. 10.1039/D1SC01850G. 34760201 PMC8565379

[ref25] For general information on the approaches to C-H activation of azines, see Murakami K.; Yamada S.; Kaneda T.; Itami K. C–H Functionalization of Azines. Chem. Rev. 2017, 117, 9302–9332. 10.1021/acs.chemrev.7b00021. 28445033

[ref26] Lai Q.; Cosio M. N.; Ozerov O. V. Ni Complexes of an Alane/Tris(phosphine) Ligand Built around a Strongly Lewis Acidic Tris(N-pyrrolyl)aluminum. Chem. Commun. 2020, 56, 14845–14848. 10.1039/D0CC05452F. 33174873

[ref27] Malpass D. B. Industrial Metal Alkyls and Their Use in Polyolefin Catalysts. In Handbook of TM Polymerization Catalysts; Hoff R., Ed.; John Wiley and Sons, Inc.: NJ, 2018; pp 1–30.

[ref28] Martineau C.; Taulelle F.; Haouas M. The Use of ^27^Al NMR to Study Aluminum Compounds: A Survey of the Last 25 Years. In PATAI’s Chemistry of Functional Groups.; Rappoport Z., 2016; pp 1–51.

[ref29] In a few (PCP)Ir(H)(Cl)(pyridine) complexes, the hydride trans to pyridine was found to resonate at −20 to −22 ppm: Hung M.-U.; Press L.; Bhuvanesh N.; Ozerov O. V. Examination of a Series of Ir and Rh PXL Pincer Complexes as (Pre)Catalysts for Aromatic C-H Borylation. Organometallics 2021, 40, 1004–1013. 10.1021/acs.organomet.1c00081.

[ref30] In (POCOP)Rh(H)(Cl)(pyridine), the hydride trans to pyridine resonates at ca. −17 ppm: Timpa S. D.; Fafard C. M.; Herbert D. E.; Ozerov O. V. Catalysis of Kumada-Tamao-Corriu Coupling by a (P°C^O^P)Rh Pincer Complex. Dalton Trans. 2011, 40, 5426–5429. 10.1039/c1dt10161g. 21394360

[ref31] Puri M.; Gatard S.; Smith D. A.; Ozerov O. V. Competition Studies of Oxidative Addition of Aryl Halides to the (PNP)Rh Fragment. Organometallics 2011, 30, 2472–2482. 10.1021/om1008956.

[ref32] Parkin G. Valence, Oxidation Number, and Formal Charge: Three Related but Fundamentally Different Concepts. J. Chem. Educ. 2006, 83, 791–799. 10.1021/ed083p791.

[ref33] Smith D. W. Valence, Covalence, Hypervalence, Oxidation State, and Coordination Number. J. Chem. Educ. 2005, 82, 1202–1204. 10.1021/ed082p1202.

[ref34] aLam W. H.; Shimada S.; Batsanov A. S.; Lin Z.; Marder T. B.; Cowan J. A.; Howard J. A. K.; Mason S. A.; McIntyre G. J. Accurate Molecular Structures of 16-Electron Rhodium Hydrido Boryl Complexes: Low-Temperature Single-Crystal X-ray and Neutron Diffraction and Computational Studies of [(PR_3_)_2_RhHCl(Boryl)] (Boryl= Bpin, Bcat). Organometallics 2003, 22 (22), 4557–4568. 10.1021/om030434d.

[ref35] Cordero B.; Gomez V.; Platero-Prats A. E.; Reves M.; Echeverria J.; Cremades E.; Barragan F.; Alvarez S. Covalent Radii Revisited. Dalton Trans. 2008, 2832–2838. 10.1039/b801115j. 18478144

[ref36] Bauer J.; Braunschweig H.; Radacki K. Transmetallation between Metal-Only Lewis Pairs: A New Rhodium Alane Complex. Chem. Commun. 2012, 48, 10407–10409. 10.1039/c2cc35248f. 22990068

[ref37] Cotton F. A. Discovering and Understanding Multiple Metal-to-Metal Bonds. Acc. Chem. Res. 1978, 11, 225–232. 10.1021/ar50126a001.

[ref38] Zhao Y.; Truhlar D. G. The M06 suite of density functionals for main group thermochemistry, thermochemical kinetics, noncovalent interactions, excited states, and transition elements: two new functionals and systematic testing of four M06-class functionals and 12 other functionals. Theor. Chem. Acc. 2008, 120, 215–241. 10.1007/s00214-007-0310-x.

[ref39] aPerutz R. N.; Sabo-Etienne S.; Weller A. S. Metathesis by Partner Interchange in σ-Bond Ligands: Expanding Applications of the σ-CAM Mechanism. Angew. Chem., Int. Ed. 2022, 61, e202111462 10.1002/anie.202111462. PMC9299125 34694734

[ref40] Crabtree R. H. Dihydrogen Complexation. Chem. Rev. 2016, 116, 8750–8769. 10.1021/acs.chemrev.6b00037. 26974601

[ref41] Eric G.; Clark L.; Weinhold F. NBO 6.0; Theoretical Chemistry Institute: University of Wisconsin: Madison, 2013.

[ref42] Herde J. L.; Lambert J. C.; Senoff C. V. Cyclooctene and 1,5-Cyclooctadiene Complexes of Iridium (I). Inorg. Synth. 1974, 15, 18–20. 10.1002/9780470132463.ch5.

[ref43] Herde J. L.; Lambert J. C.; Senoff C. V. Cyclooctene and 1,5-cyclooctadiene complexes of iridium (I). Inorg. Synth. 1974, 15, 18–20. 10.1002/9780470132463.ch5.

[ref44] Giordano G.; Crabtree R. H. Di-μ-chloro-bis(η^4^-1,5-cyclooctadiene)dirhodium(I). Inorg. Synth. 1990, 28, 88–90. 10.1002/9780470132593.ch22.

