# Supplementary material for: Migration of Hydride, Methyl, and Chloride Ligands between Al and M in (PAlP)M Pincer Complexes (M = Rh or Ir)
Source: Organometallics. 2023 Oct 24;42(21):3120–9. doi: 10.1021/acs.organomet.3c00359 (PMC10863399; doi:10.1021/acs.organomet.3c00359)
Supplement: Supplementary file 1 — om3c00359_si_001.pdf [file om3c00359_si_001.pdf]

Supporting Information

Migration of Hydride, Methyl and Chloride Ligands between Al and M in (PAlP)M Pincer Complexes (M = Rh or Ir)

Vinh T. Nguyen[a†], Qingheng Lai[a†], Naphol Witayapaisitsan[b], Nattamai Bhuvanesh[a], Panida Surawatanawong[b*], and Oleg V. Ozerov[a]*

[a] *Department of Chemistry, Texas A&M University, 3255 TAMU, College Station, Texas 77842, United States*

[b] *Department of Chemistry and Center of Excellence for Innovation in Chemistry, Faculty of Science, Mahidol University, Bangkok 10400, Thailand*

[†]V.T.N. and Q.L. contributed equally to this paper.

ozerov@chem.tamu.edu

panida.sur@mahidol.ac.th

Table of Contents

I. General Considerations.. **S3**

II. Selected NMR Data Tables.. **S4**

III. NMR Spectra.. **S7**

IV. X-ray Structural Determination Details... **S49**

V. DFT Computational Studies.. **S59**

VI. Reference.. **S66**

I. General Considerations

All manipulations were performed under an Ar atmosphere using standard Schlenk line or glovebox techniques. Pentane, tetrahydrofuran, diethyl ether, toluene, and isooctane were dried and deoxygenated (by purging) using a solvent purification system (Innovative Technology Pure Solv MD-5 Solvent Purification System) and stored over molecular sieves in an Ar-filled glove box. C_6D_6, $CDCl_3$, and pyridine were dried over calcium hydride, vacuum transferred, and stored over molecular sieves in an Ar-filled glovebox. Celite and silica were dried at 180 °C under vacuum overnight and stored in an Ar-filled glovebox. Compound **1**,[1] $[Ir(COE)_2Cl]_2$[2], $[Ir(COD)Cl]_2$[3], and $[Rh(COD)Cl]_2$[4] and were prepared according to literature procedures. All other chemicals were used as received from commercial vendors. NMR spectra were recorded on a Varian VnmrS 500 (^{1}H NMR, 500 MHz; ^{13}C{^{1}H} NMR, 126 MHz; ^{31}P{^{1}H} NMR, 202 MHz; ^{27}Al{^{1}H} NMR, 130 MHz), Varian Inova 500 (^{1}H NMR, 500 MHz; ^{13}C{^{1}H} NMR, 126 MHz; ^{31}P{^{1}H} NMR, 202 MHz), and Avance NEO 400 (^{1}H NMR, 400 MHz; ^{13}C{^{1}H} NMR, 101 MHz; ^{31}P{^{1}H} NMR, 162 MHz) spectrometers. Chemical shifts are reported in δ (ppm). For ^{1}H and ^{13}C NMR spectra, the residual solvent peak was used as an internal reference (^{1}H NMR: δ 7.16 for C_6D_6 and 7.26 ppm for $CDCl_3$; ^{13}C NMR: δ 128.06 for C_6D_6 and 77.16 ppm for $CDCl_3$.^{31}P NMR spectra were referenced externally with 85% phosphoric acid at δ 0 ppm. ^{27}Al NMR spectra were referenced externally with an aqueous solution of aluminum nitrate at δ 0. Elemental analyses were performed by CALI Labs, Inc. (Highland Park, NJ).

II. Selected NMR Data Tables

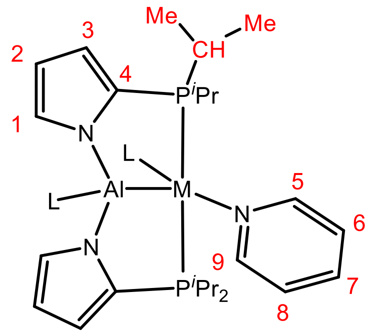

Table S1. ^{1}H NMR chemical shifts (ppm) of the aromatic peaks for Ir and Rh complexes in C_6D_6. Coupling constants (Hz) and multiplicity are shown in parenthesis for J_{HH}> 5 Hz.

Compound	C_1-*H*	C_2-*H*	C_3-*H*	C_5-*H*/ C_9-*H*	C_6-*H*/ C_8-*H*	C_7-*H*
**3-Rh**	7.61	6.78	6.78	9.57<br>8.52	6.37	6.63
**3-Ir**	7.63	6.78	6.78	9.81<br>8.48	6.32<br>6.29	6.58
**4-Rh**	7.57	6.81	6.65	8.81	6.46	6.70
**4-Ir**	7.61	6.85	6.64	8.83	6.37<br>(t,7)	6.58<br>(t, 8)
**5-Rh**	7.55	6.88	6.68	8.61	6.39<br>(t,7)	6.66<br>(t, 8)
**6-Ir**	7.72	6.91	6.62	8.76	6.08<br>(t,7)	6.56<br>(t,7)

Table S2. ^{1}H NMR chemical shifts (ppm) of the isopropyl peaks for Ir and Rh complexes in C_6D_6.

**Compound**	**CH***		**Me#**			
**3-Rh**	2.09	2.41	0.34 (7)	0.89 (7)	1.23 (7)	1.31 (8)
**3-Ir**	2.35	2.61	0.37	0.86 (m)	1.24 (m)	1.29 (m)
**4-Rh**	1.60	2.19	0.78 (8)	0.94 (7)	1.07 (m)	1.12 (m)
**4-Ir**	1.73	2.49	0.78 (8)	0.92 (7)	1.01 (8)	1.14 (8)
**5-Rh**	1.63	2.20	0.70 (8)	0.94 (7)	1.12 (8)	1.18 (8)
**6-Ir**	1.28	2.23	0.93 (m, 12H)	0.93 (m, 12H)		

#Peaks are (dvt, 6H), unless otherwise noted. Values listed in parenthesis are $J_{H,P} \approx J_{H,H}$.
*Peaks are (m, 2H).

Table S3. $^{13}C\{^1H\}$ NMR chemical shifts (ppm) and *J* values (Hz) of the aromatic peaks for the Ir and Rh complexes.

Compound	*C*1*	*C*2*	*C*3*	*C*4*	*C*5/ *C*9^	*C*6/ *C*8^	*C*7^
**3-Rh** (C_6D_6)	112.6 (3)	115.7 (4)	128.4 (m)	130.4 (33)	156.0 (br s) 148.5 (br s)	125.6 (br s) 123.6 (br s)	136.3
**3-Ir** (C_6D_6)	112.3 (3)	115.5 (5)	#	130.0 (40)	156.5 147.7	126.2 123.7	135.4
**4-Rh** (C_6D_6)	113.4 (3)	114.9 (4)	128.7 (7)	#	151.5	124.5	136.1
**4-Ir** ($CDCl_3$)	112.0 (4)	113.9 (6)	127.2 (8)	130.0 (40)	150.9	125.6	136.5
**5-Rh** ($CDCl_3$)	111.5 (3)	113.3 (4)	127.1 (7)	129.8 (35)	151.6	125.0	136.5
**6-Ir** ($CDCl_3$)	112.8 (3)	114.5 (5)	129.0 (6)	128.1 (39)	159.6	126.4	136.2

*Peaks are (vt) and values listed in parenthesis are $J_{C,P}$, unless otherwise noted.
^Peaks are (s), unless otherwise noted.
#$C_{Pyrrole}$ triplet overlaps with C_6D_6.

Table S4. $^{13}C\{^1H\}$ NMR chemical shifts (ppm) and *J* values (Hz) of the isopropyl peaks for Ir and Rh complexes. Peaks are (vt) and values listed in parenthesis are $J_{C,P}$, unless otherwise noted.

Compound	CH*		Me			
**3-Rh** (C_6D_6)	26.4 (12)	28.5 (13)	18.7 (m)	18.8 (s)	20.0 (s)	22.8 (5)
**3-Ir** (C_6D_6)	25.9 (15)	28.8 (16)	18.7 (m)	19.1 (s)	19.6 (s)	22.6 (4)
**4-Rh** (C_6D_6)	27.0 (12)	28.2 (15)	18.1 (s)	19.4 (3)	20.5 (4)	21.4 (s)
**4-Ir** ($CDCl_3$)	27.0 (18)	28.2 (22)	18.1 (s)	19.0 (1)	20.3 (4)	21.3 (s)
**5-Rh** ($CDCl_3$)	26.7 (12)	28.3 (15)	18.1 (s)	19.4 (2)	20.5 (4)	21.3 (s)
**6-Ir** ($CDCl_3$)	24.7 (15)	25.1 (20)	16.0 (s)	18.0 (3)	19.3 (2)	19.4 (s)

III. NMR Spectra

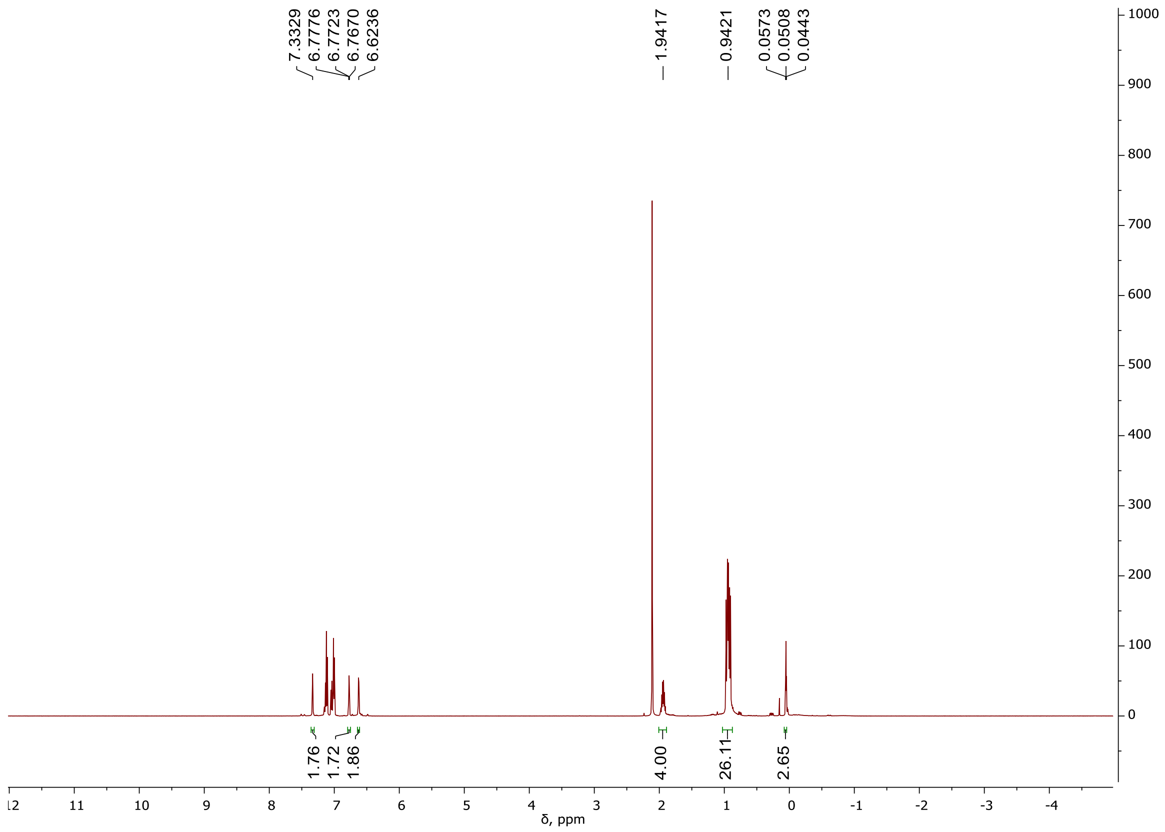

Figure S1. ^{1}H NMR (500 MHz, C_6D_6) spectrum of **2-Me**. Residual toluene and methane present.

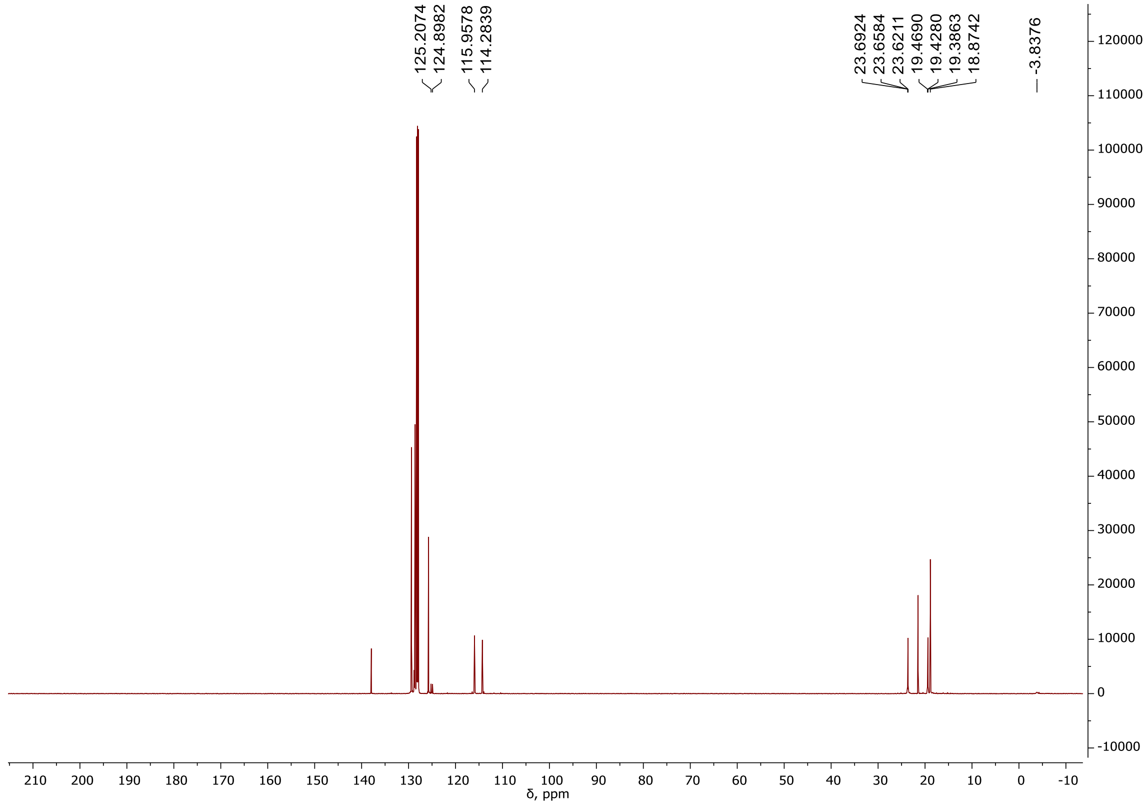

Figure S2. $^{13}C\{^{1}H\}$ NMR (126 MHz, C_6D_6) spectrum of **2-Me**.

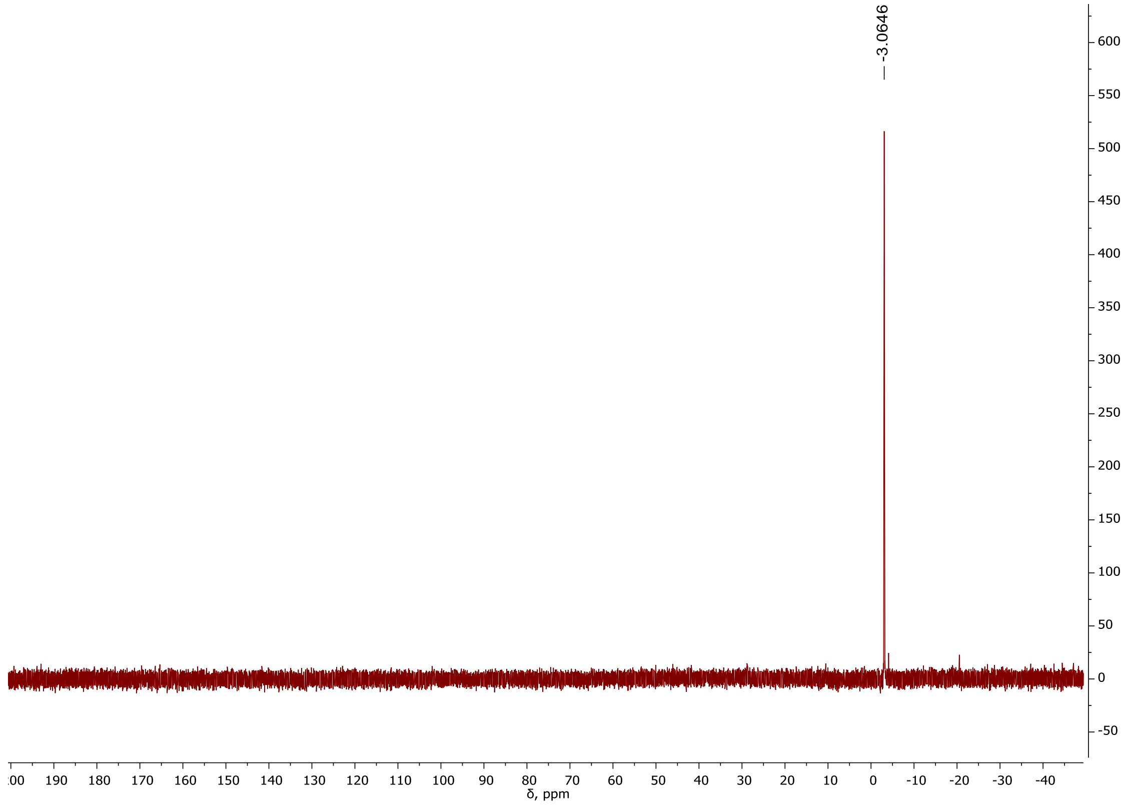

Figure S3. $^{31}P\{^{1}H\}$ NMR (202 MHz, C_6D_6) spectrum of **2-Me**.

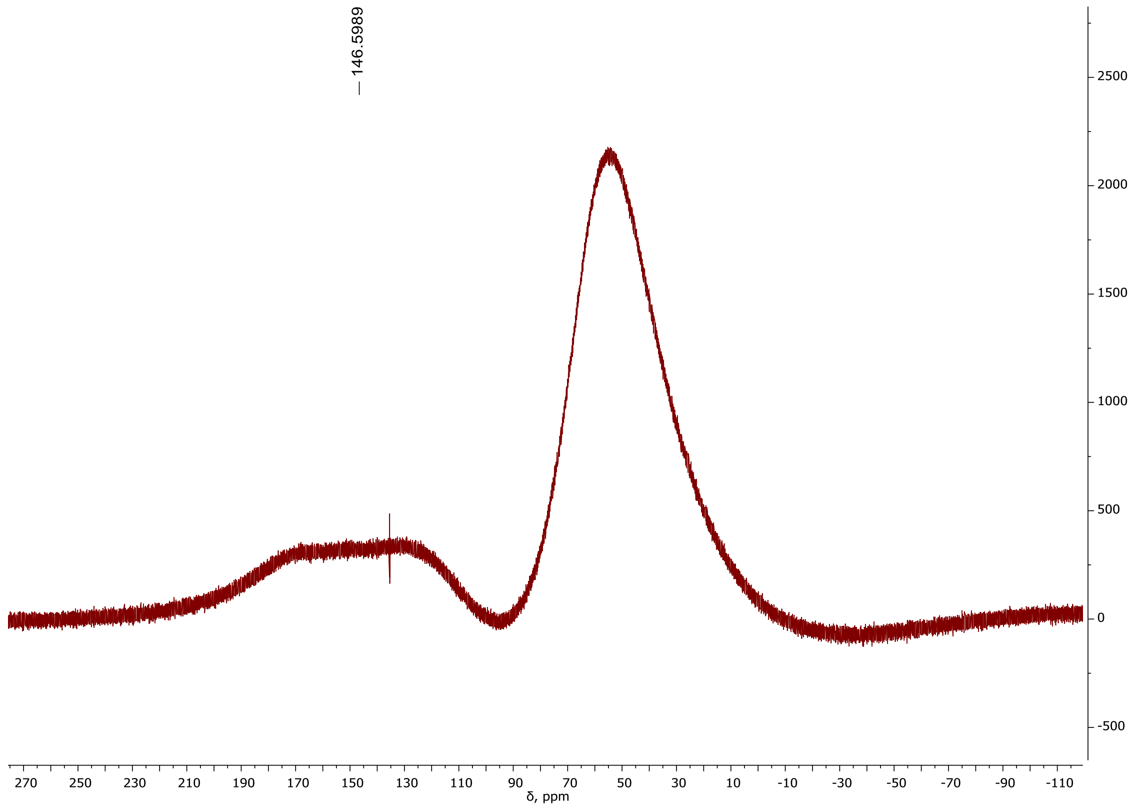

Figure S4. $^{27}Al\{^{1}H\}$ NMR (130 MHz, C_6D_6) spectrum of **2-Me**.

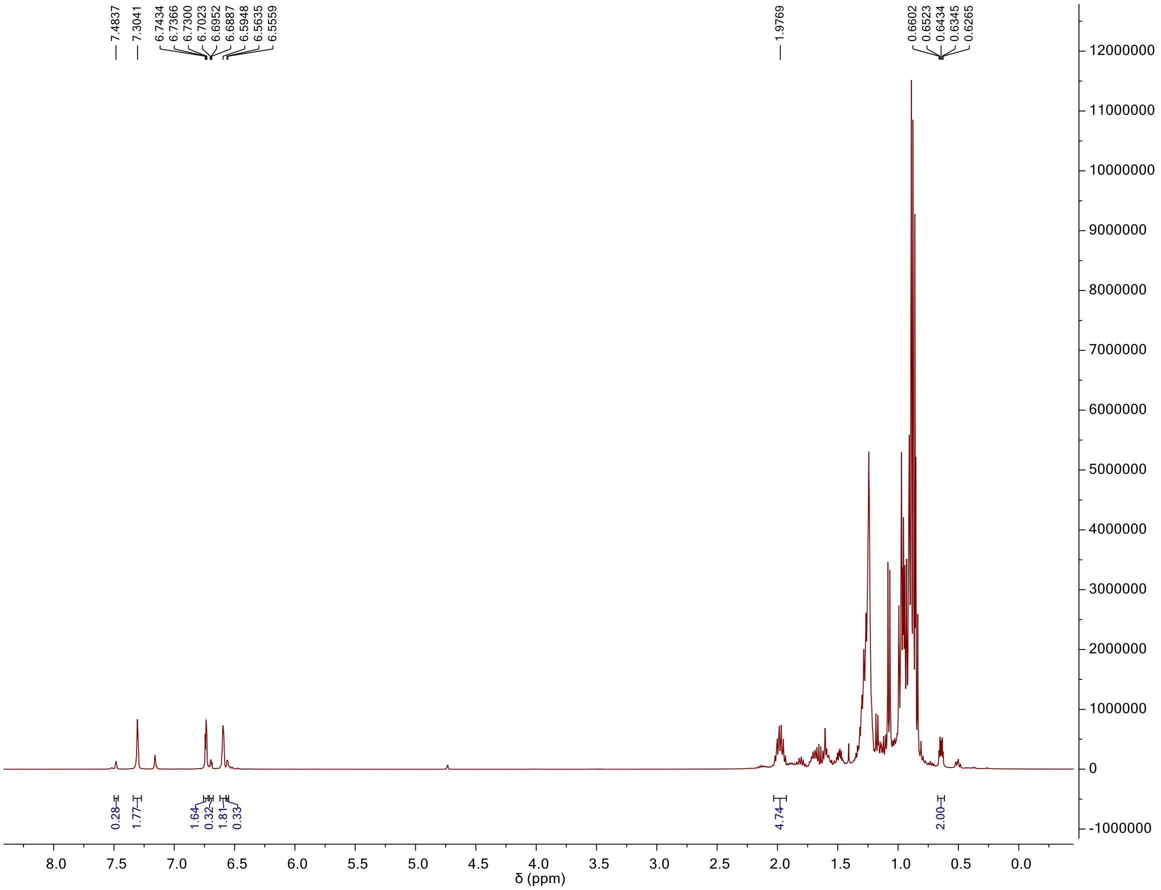

Figure S5. ^{1}H NMR (400 MHz, C_6D_6) spectrum of an *in situ* reaction mixture of two equiv 1 and an equiv of $Al^{i}Bu_3$. A mixture of **2-^{i}Bu** and **2-H** are formed.

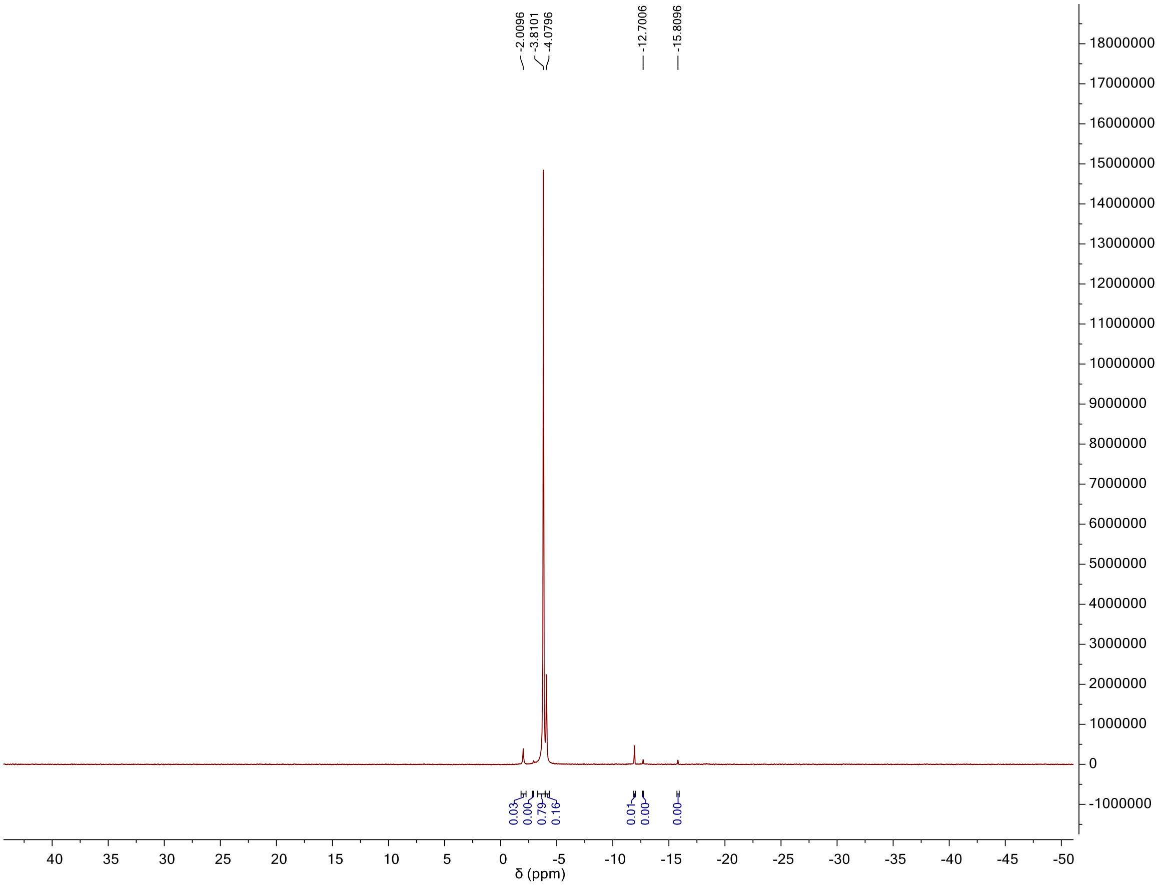

Figure S6. $^{31}P\{^{1}H\}$ NMR (162 MHz, C_6D_6) spectrum of an *in situ* reaction mixture of two equiv 1 and an equiv of $Al^{i}Bu_3$. A mixture of **2-^{i}Bu** and **2-H** are formed.

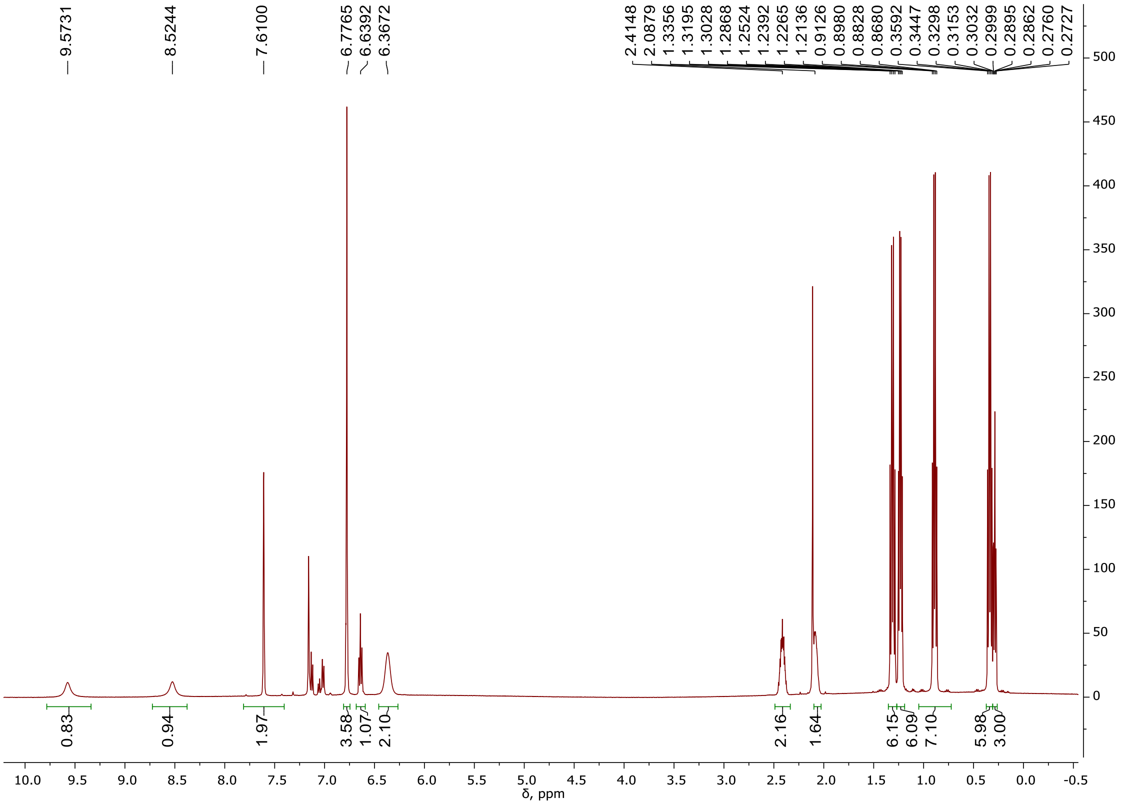

Figure S7. ^{1}H NMR (500 MHz, C_6D_6) spectrum of **3-Rh**. Residual toluene present.

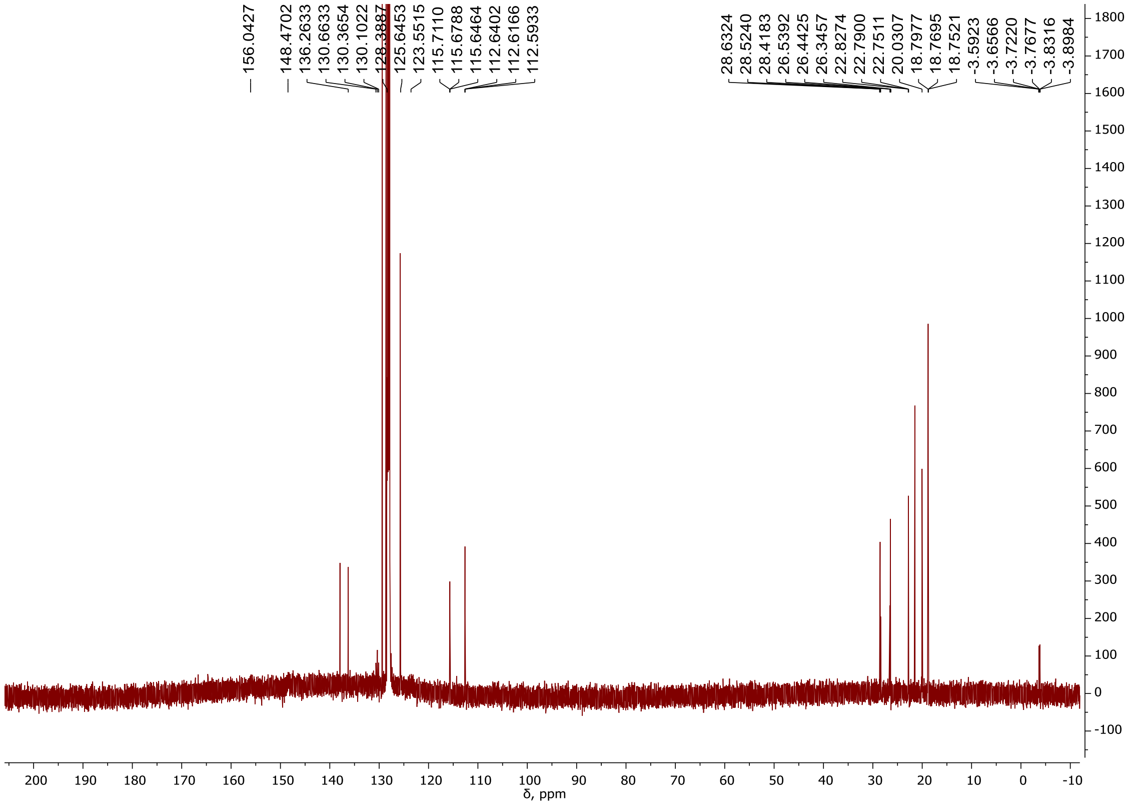

Figure S8. $^{13}C\{^{1}H\}$ NMR (126 MHz, C_6D_6) spectrum of **3-Rh**. Residual toluene present.

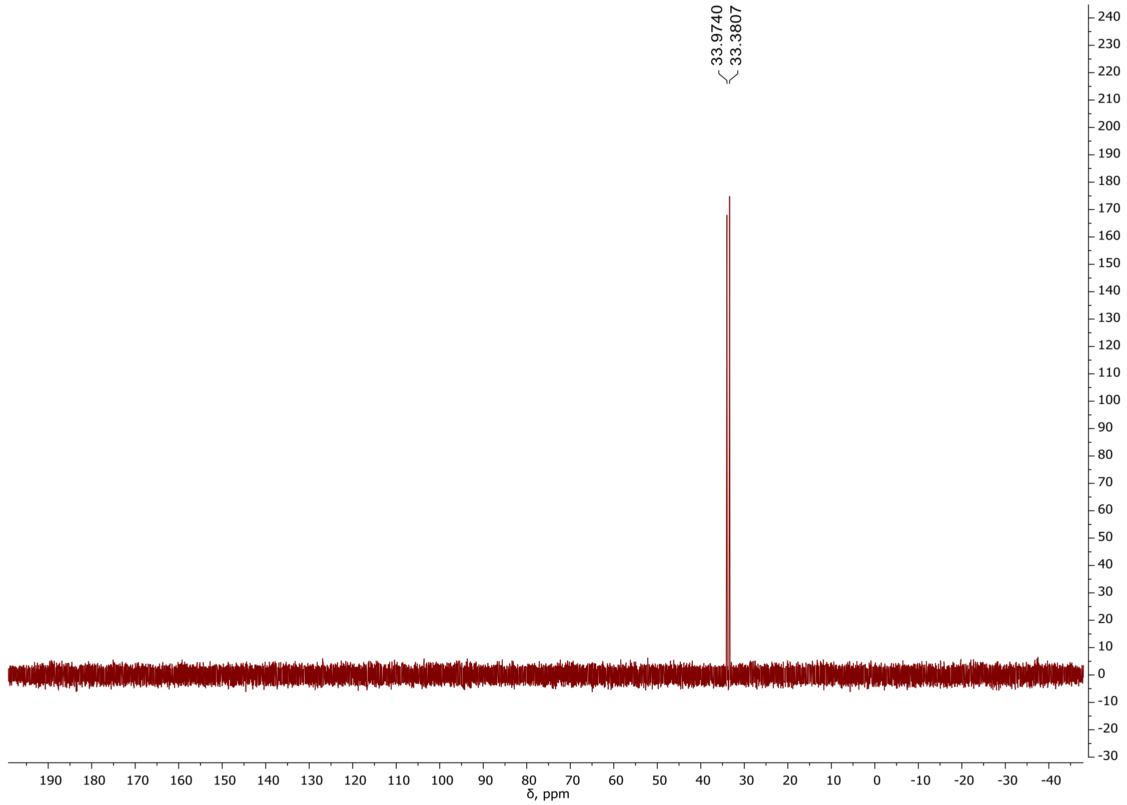

Figure S9. $^{31}P\{^{1}H\}$ NMR (202 MHz, C_6D_6) spectrum of **3-Rh**.

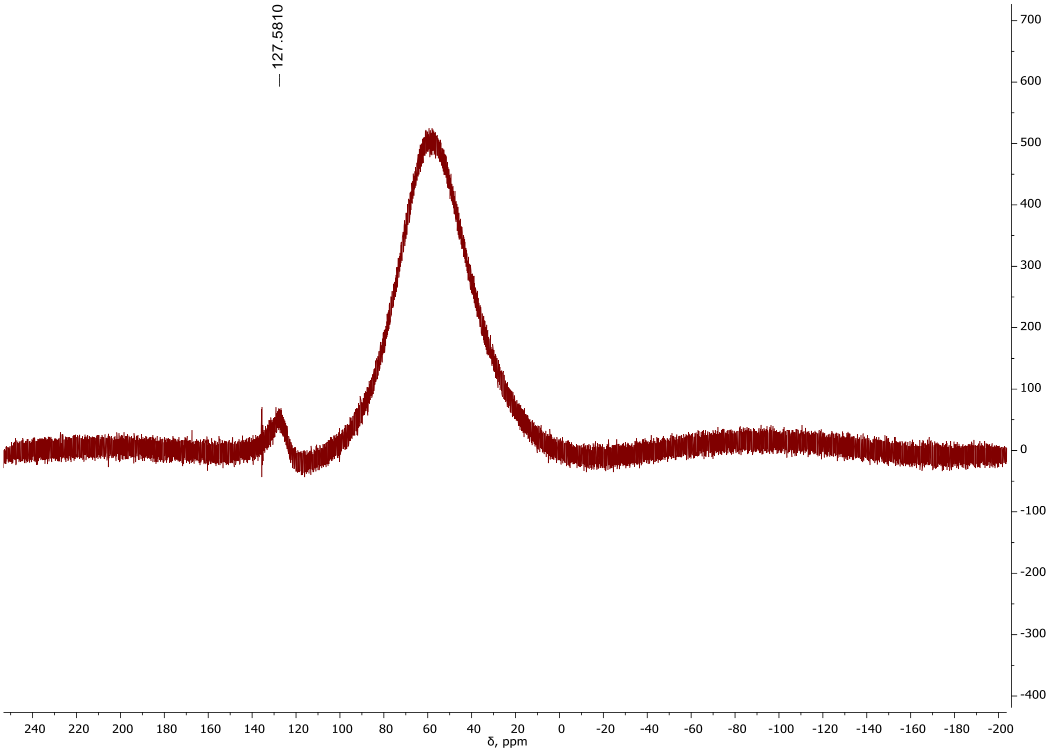

Figure S10. $^{27}Al\{^1H\}$ NMR (130 MHz, C_6D_6) spectrum of **3-Rh**.

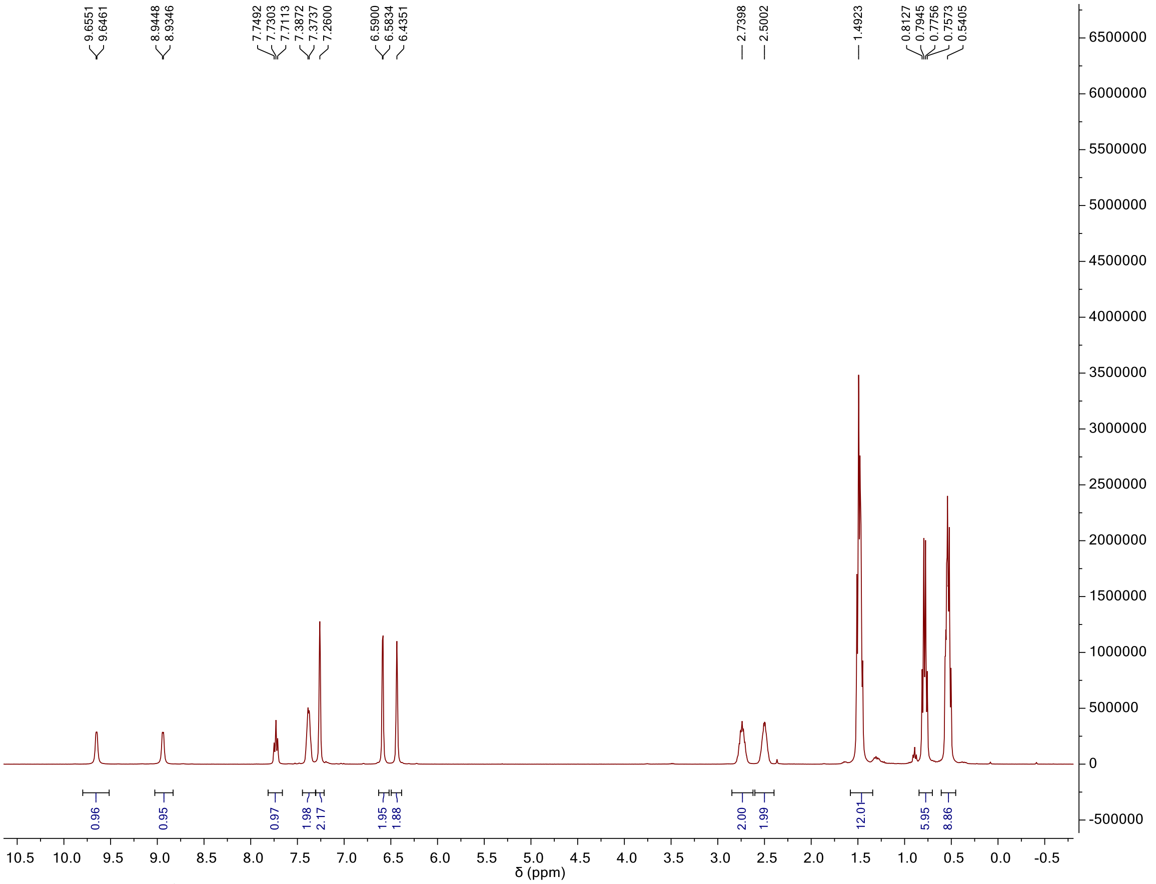

Figure S11. ^{1}H NMR (400 MHz, $CDCl_3$) spectrum of **3-Ir**. Residual pentane present.

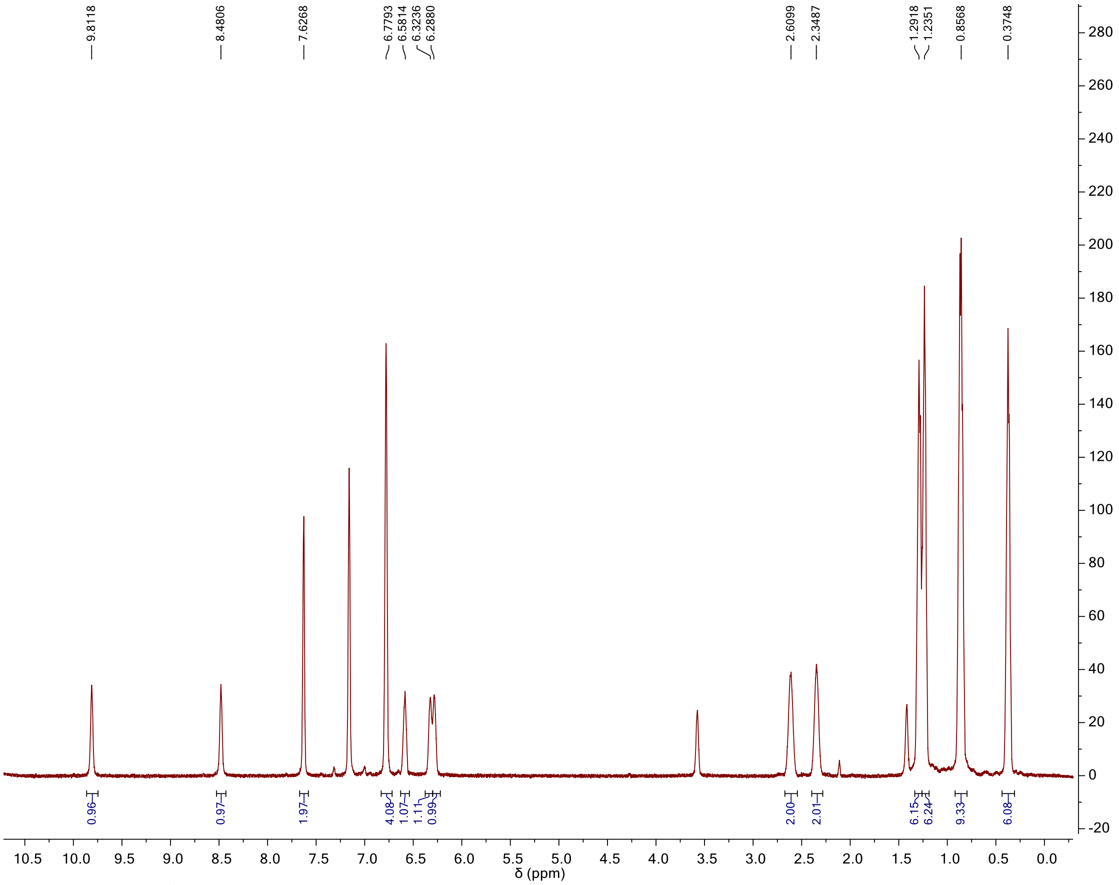

Figure S12. ^{1}H NMR (500 MHz, C_6D_6) spectrum of **3-Ir**. Residual THF present.

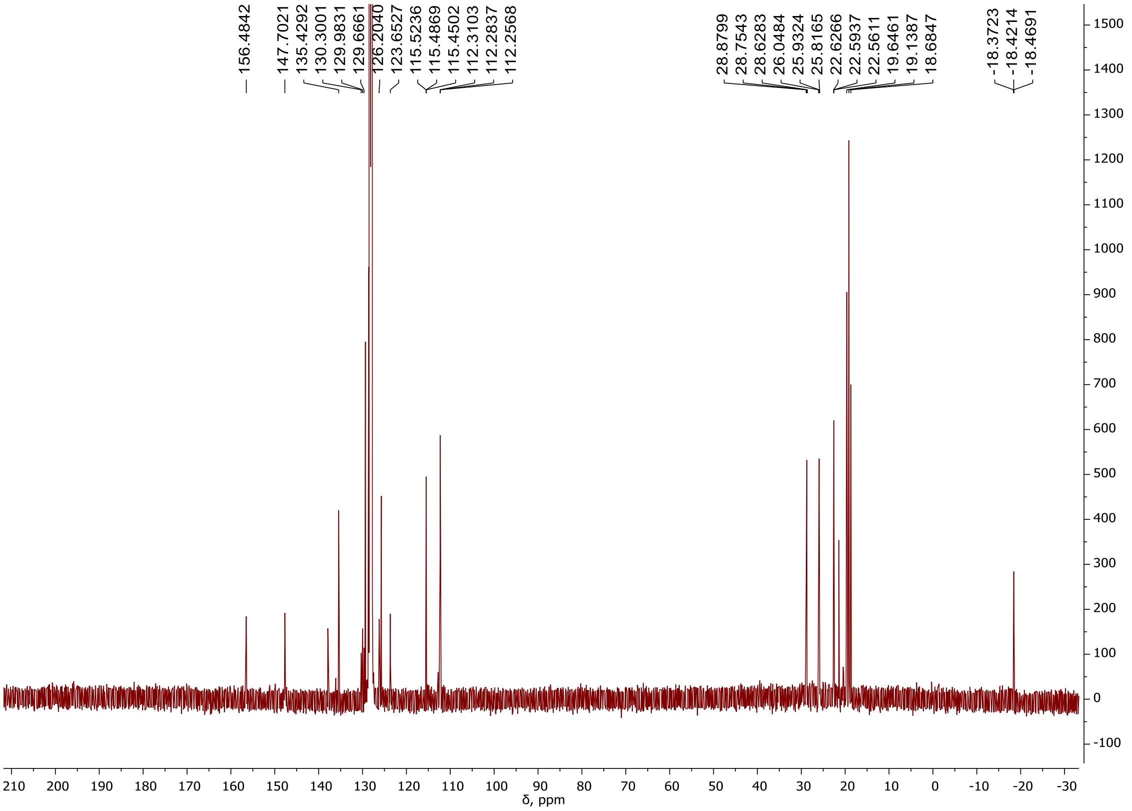

Figure S 13. $^{13}C\{^{1}H\}$ NMR (126 MHz, C_6D_6) spectrum of **3-Ir**. Residual toluene peaks present.

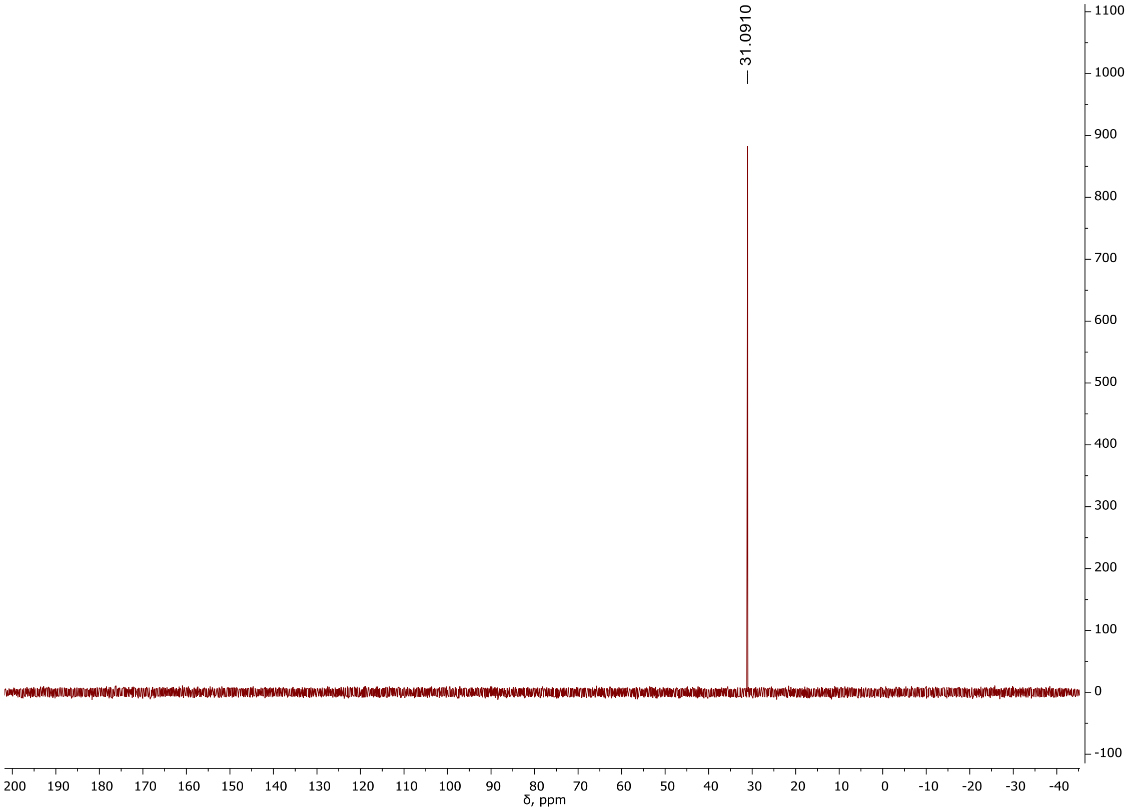

Figure S 14. $^{31}P\{^1H\}$ NMR (202 MHz, C_6D_6) spectrum of **3-Ir**.

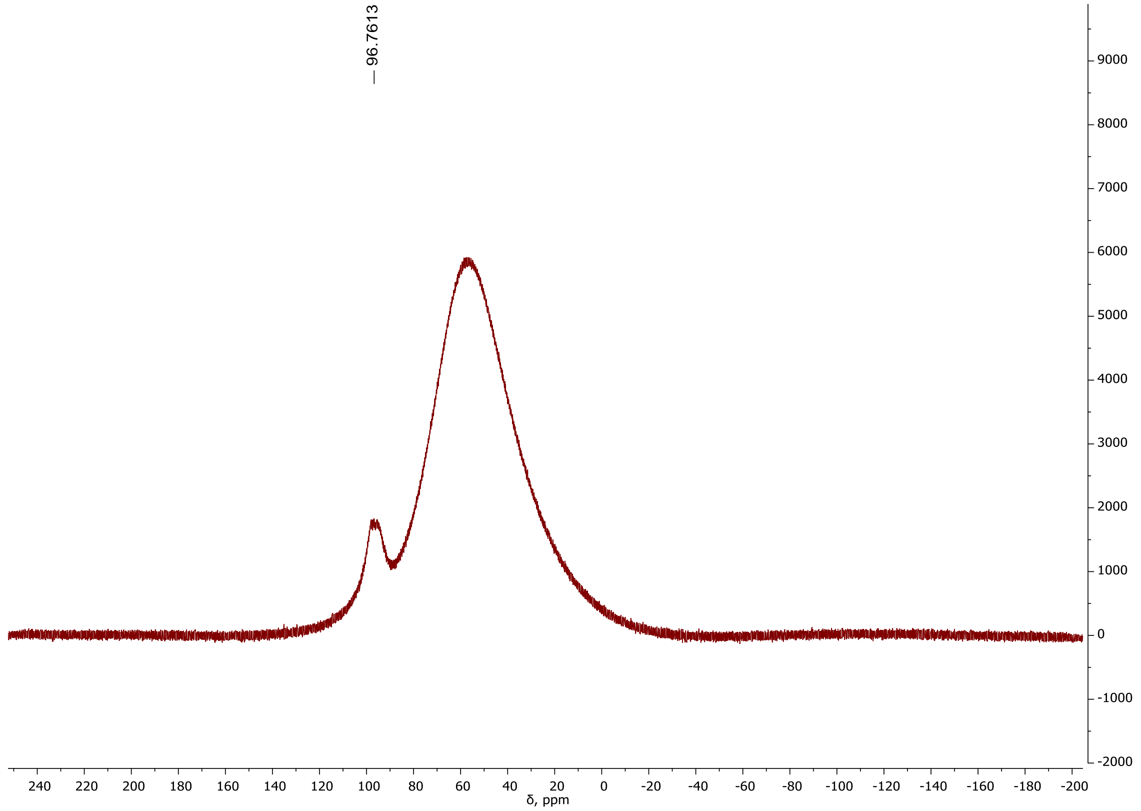

Figure S15. $^{27}Al\{^1H\}$ NMR (130 MHz, C_6D_6) spectrum of **3-Ir**.

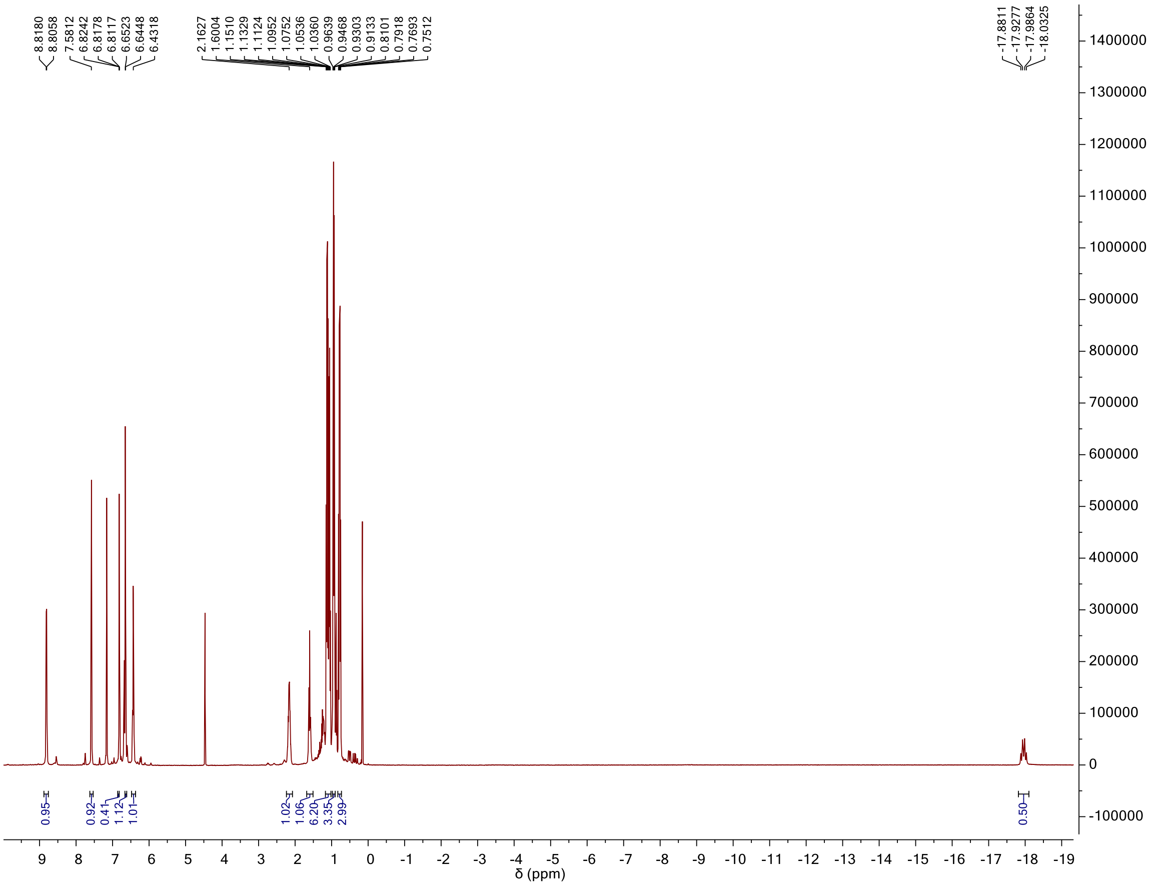

Figure S16. ^{1}H NMR (400 MHz, C_6D_6) spectrum of the *in situ* reaction mixture of **3-Rh** under an atmosphere of H_2 to form **4-Rh**. H_2 and methane gas detected.

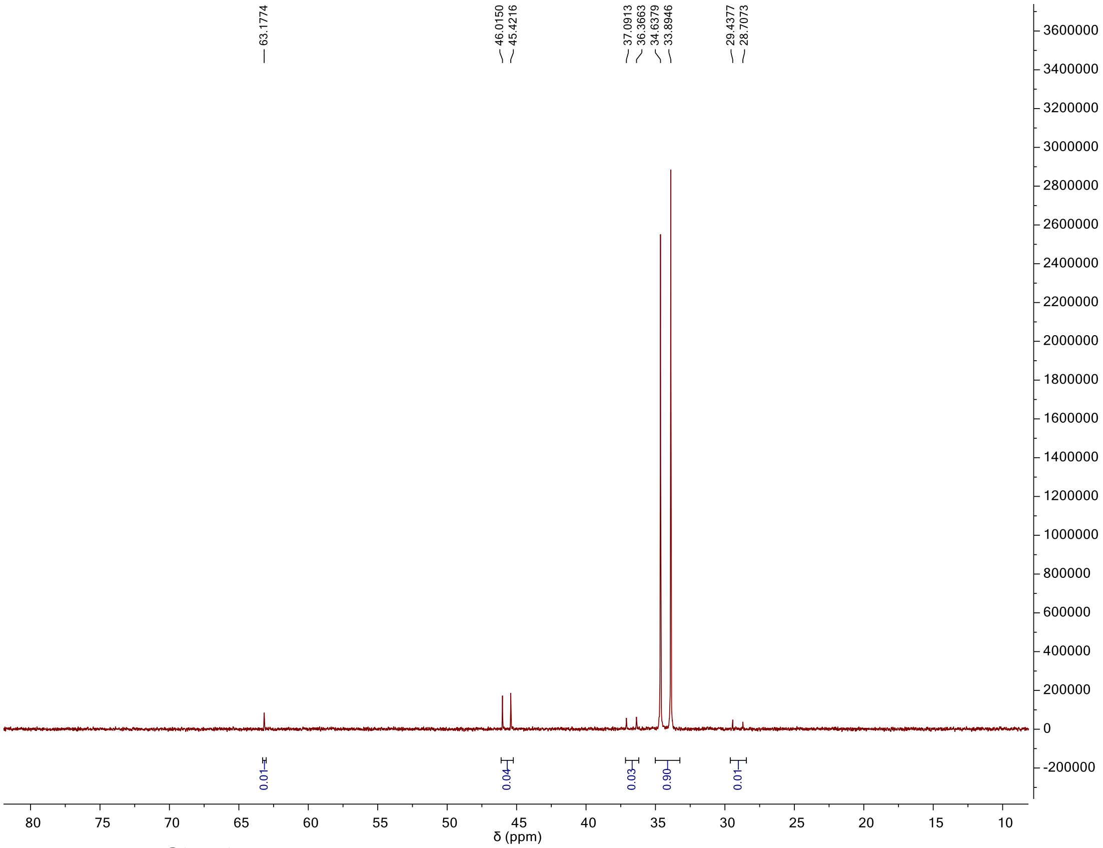

Figure S17. $^{31}P\{^{1}H\}$ NMR (162 MHz, C_6D_6) spectrum of the *in situ* reaction mixture of 3-Rh under an atmosphere of H_2 to form **4-Rh**.

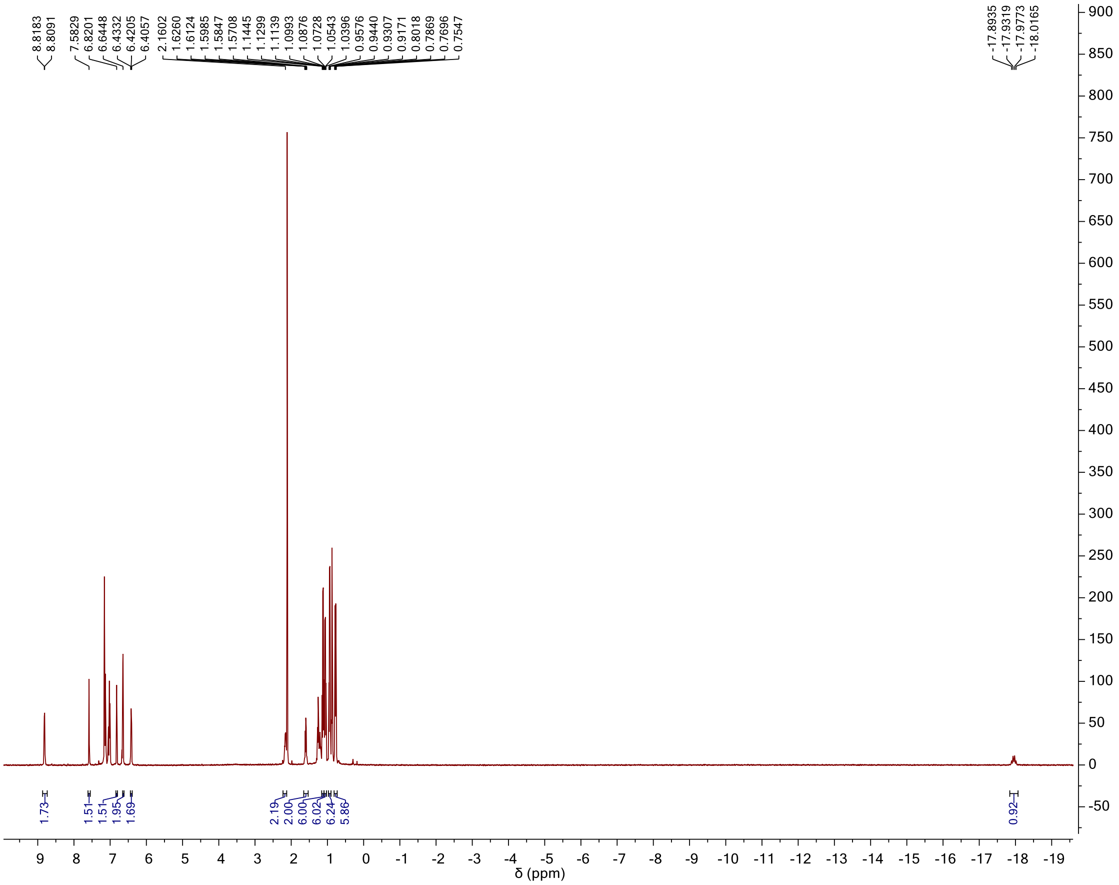

Figure S18. ^{1}H NMR (500 MHz, C_6D_6) spectrum of **4-Rh**. Residual toluene and pentane present.

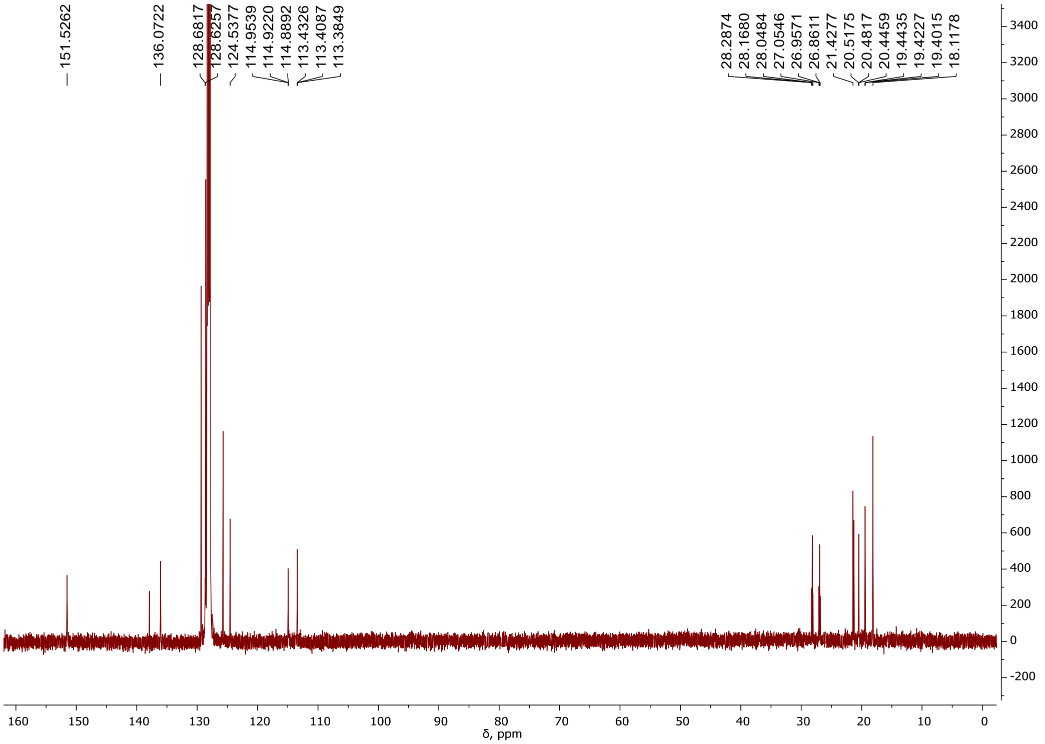

Figure S19. $^{13}C\{^{1}H\}$ NMR (126 MHz, C_6D_6) spectrum of **4-Rh**. Residual toluene present.

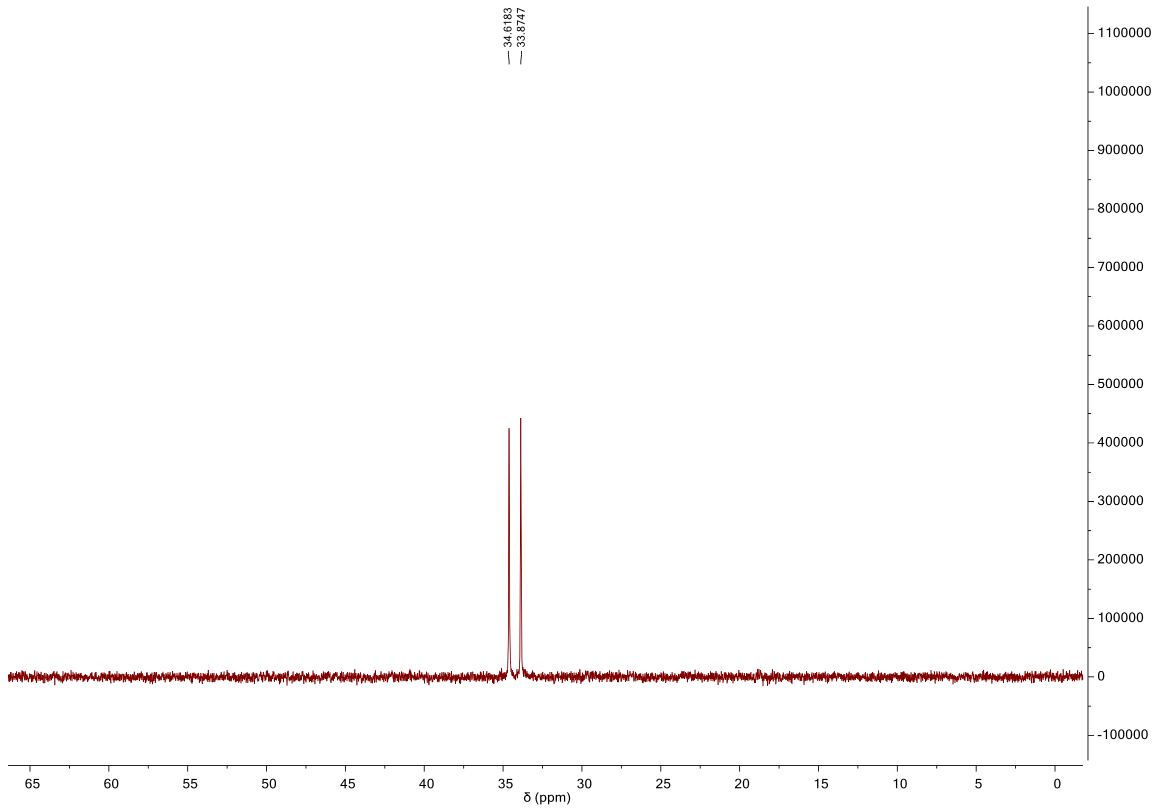

Figure S20. $^{31}P\{^{1}H\}$ NMR (162 MHz, C_6D_6) spectrum of **4-Rh**.

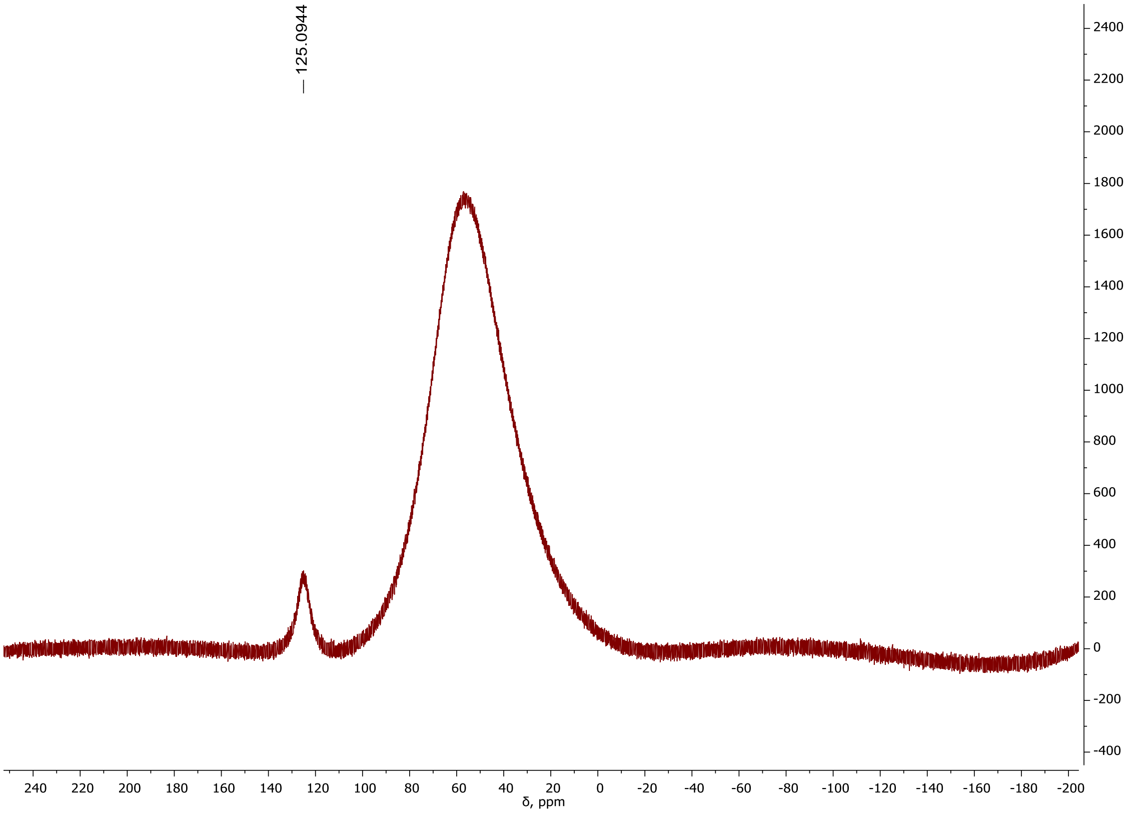

Figure S21. $^{27}Al\{^{1}H\}$ NMR (130 MHz, C_6D_6) spectrum of **4-Rh**.

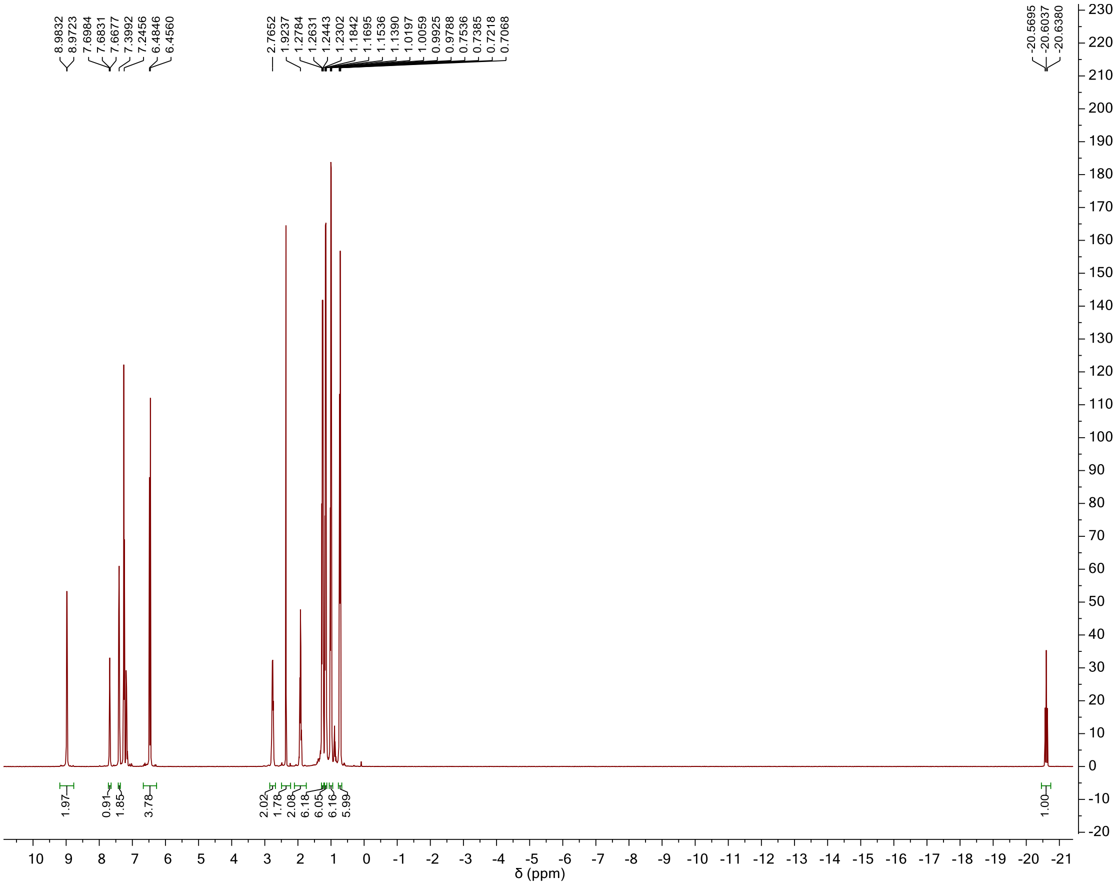

Figure S22. ^{1}H NMR (500 MHz, $CDCl_3$) spectrum of **4-Ir**.

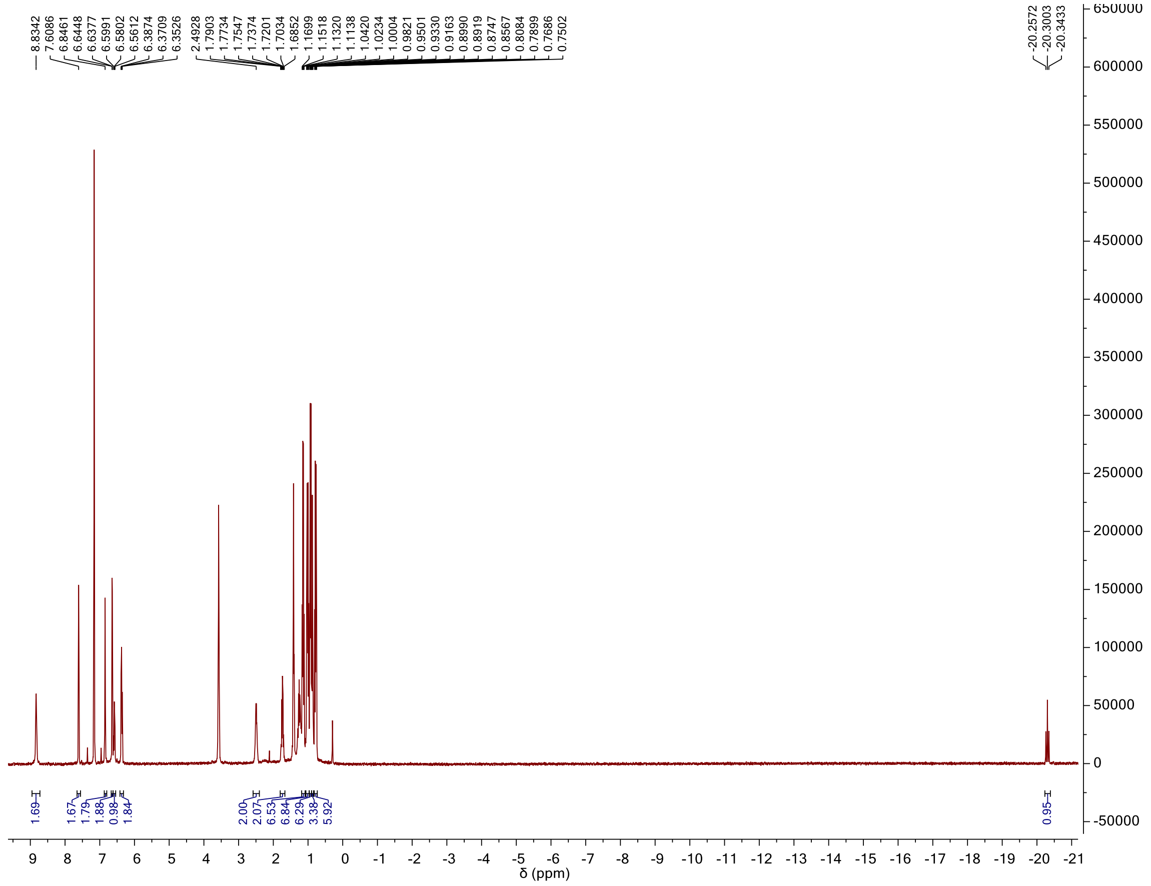

Figure S23. ^{1}H NMR (400 MHz, C_6D_6) spectrum of **4-Ir**.

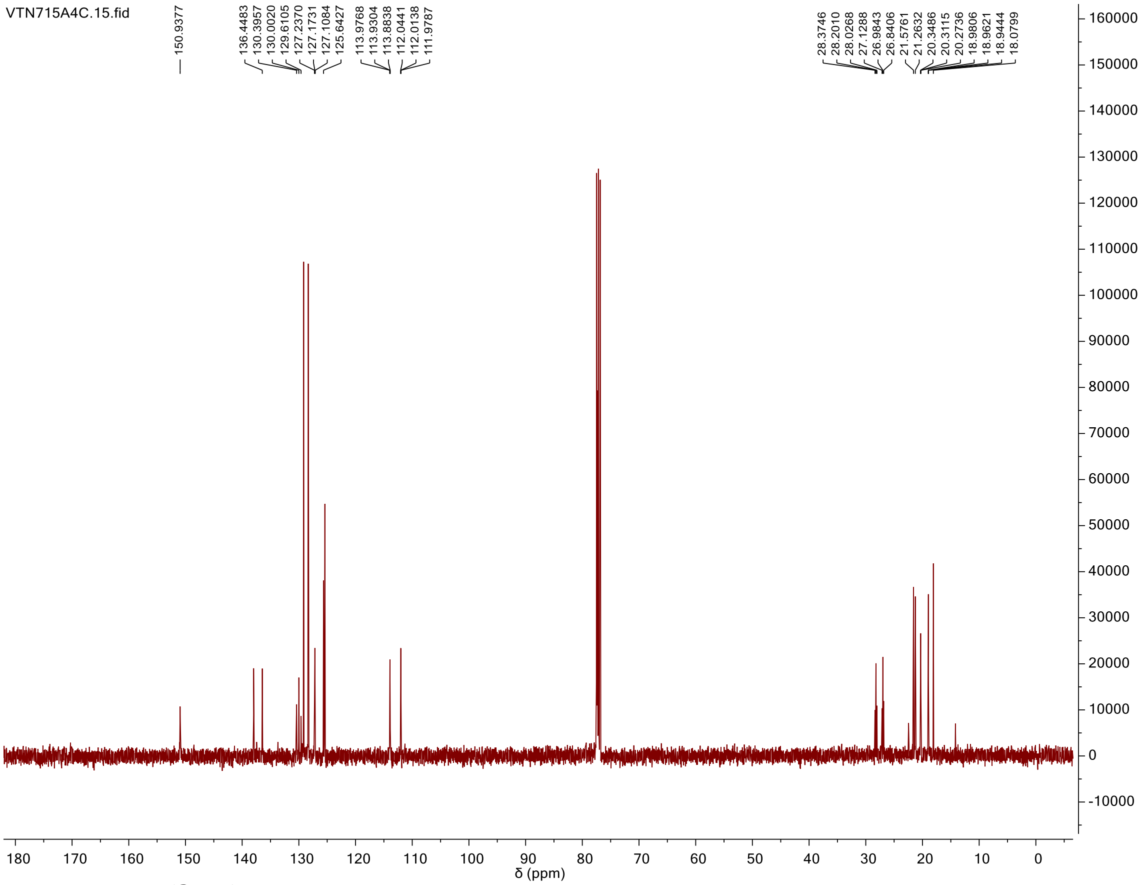

Figure S24. $^{13}C\{^1H\}$ NMR (101 MHz, $CDCl_3$) spectrum of **4-Ir**.

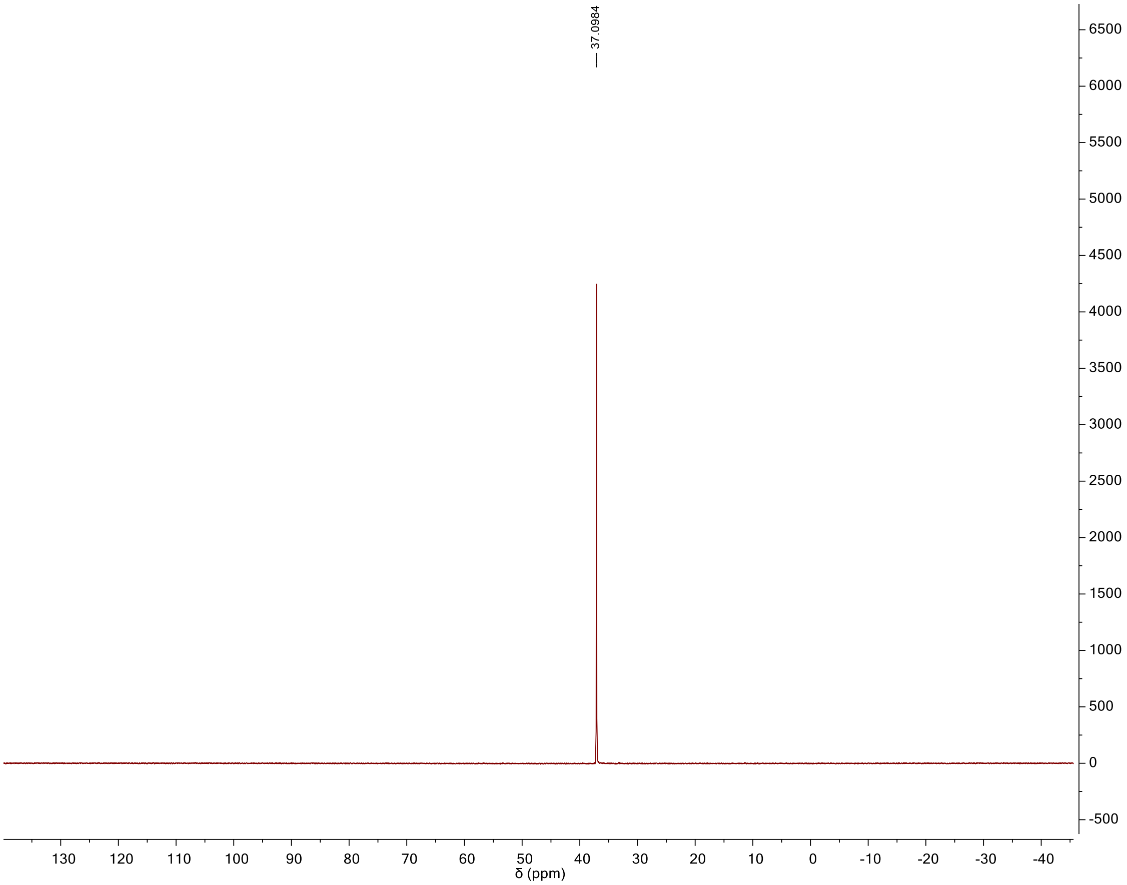

Figure S25. $^{31}P\{^{1}H\}$ NMR (202 MHz, $CDCl_3$) spectrum of **4-Ir**.

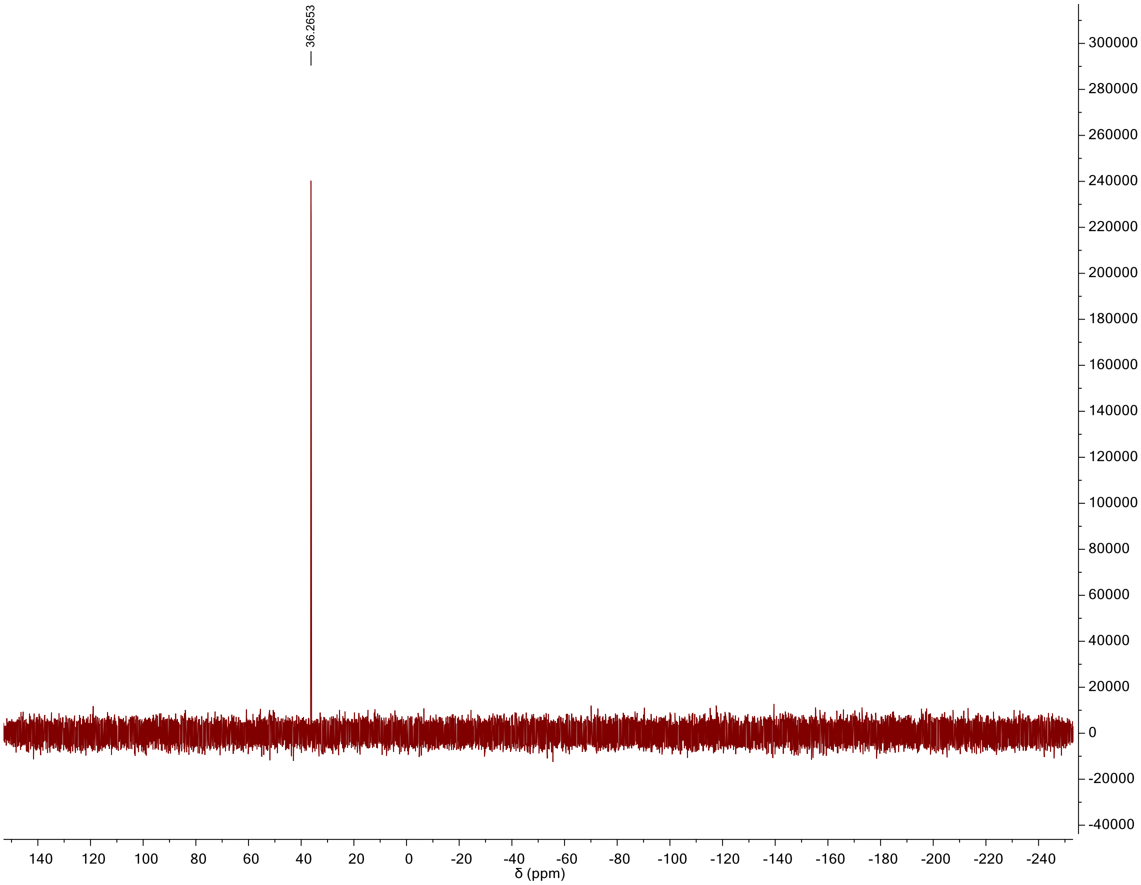

Figure S26. $^{31}P\{^1H\}$ NMR (162 MHz, C_6D_6) spectrum of **4-Ir**.

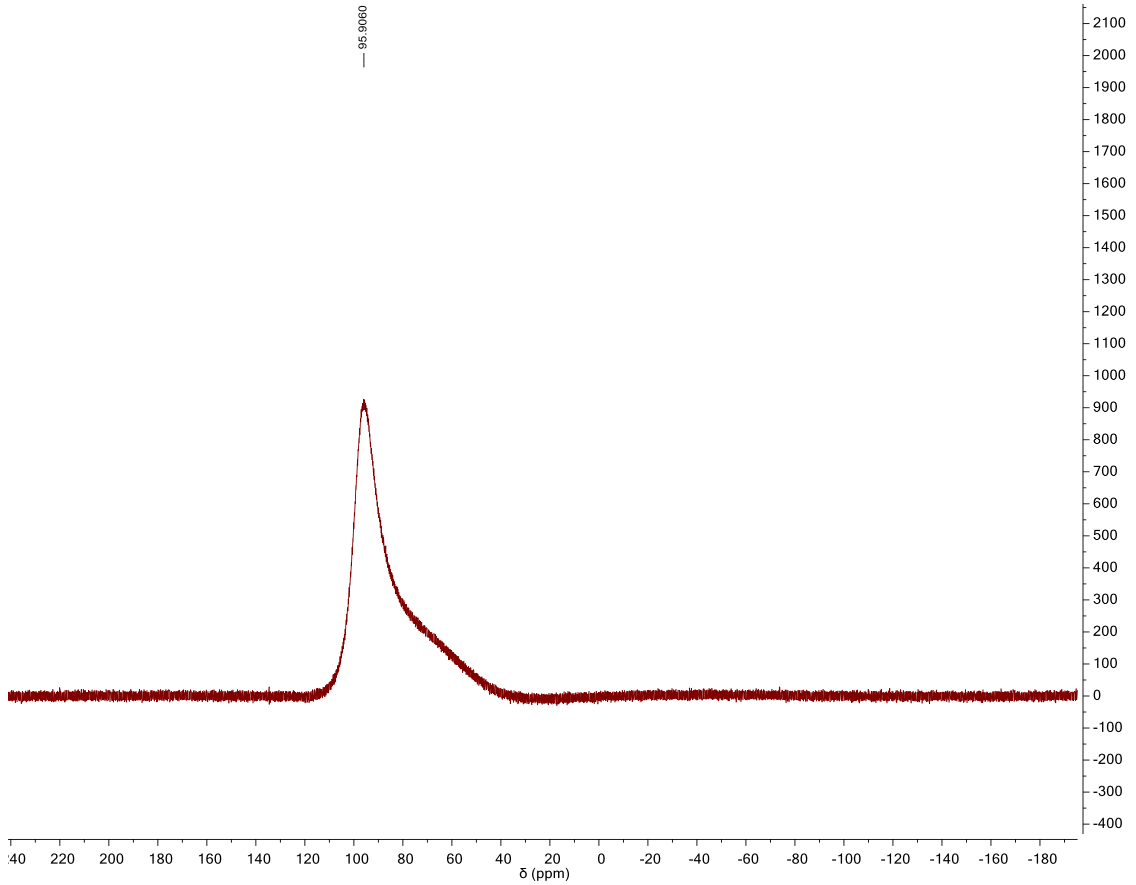

Figure S27. $^{27}Al\{^1H\}$ NMR (130 MHz, $CDCl_3$) spectrum of **4-Ir**.

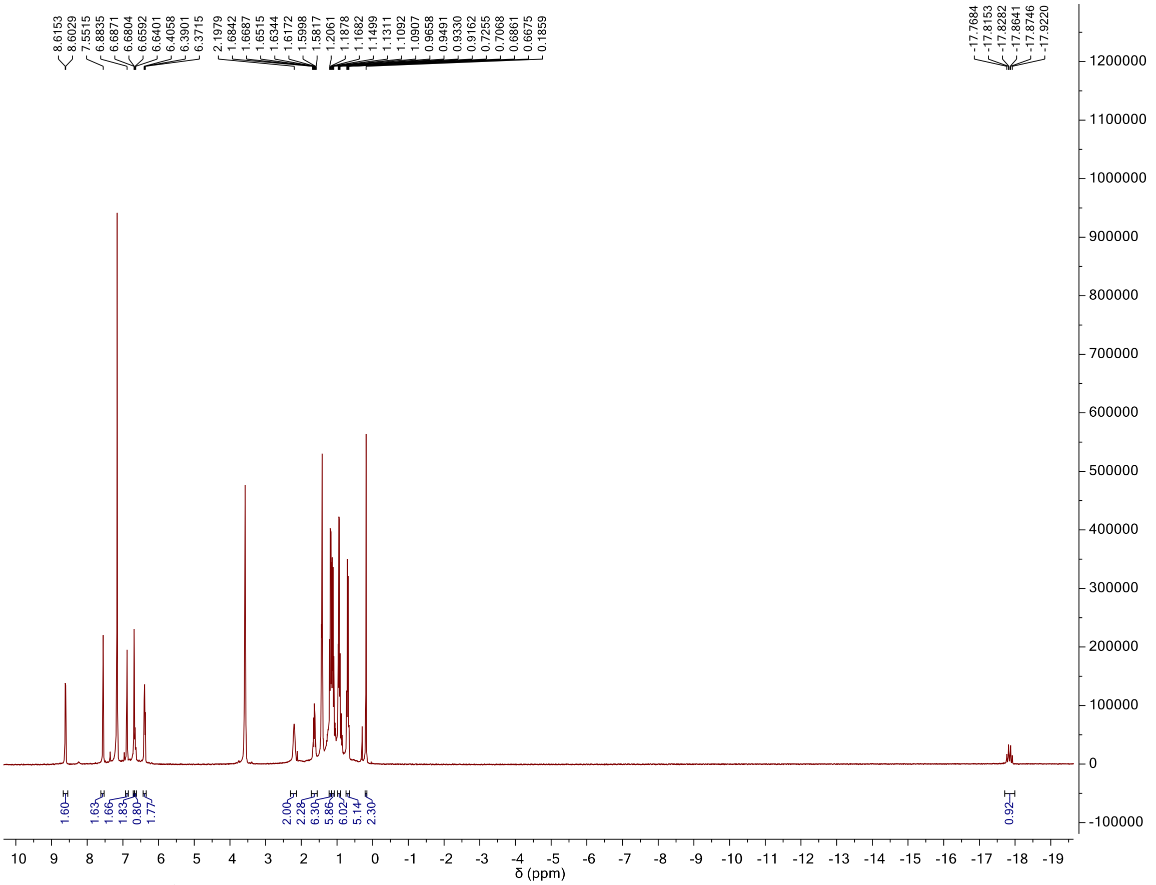

Figure S28. ^{1}H NMR (400 MHz, C_6D_6) spectrum of **5-Rh**. Residual THF present.

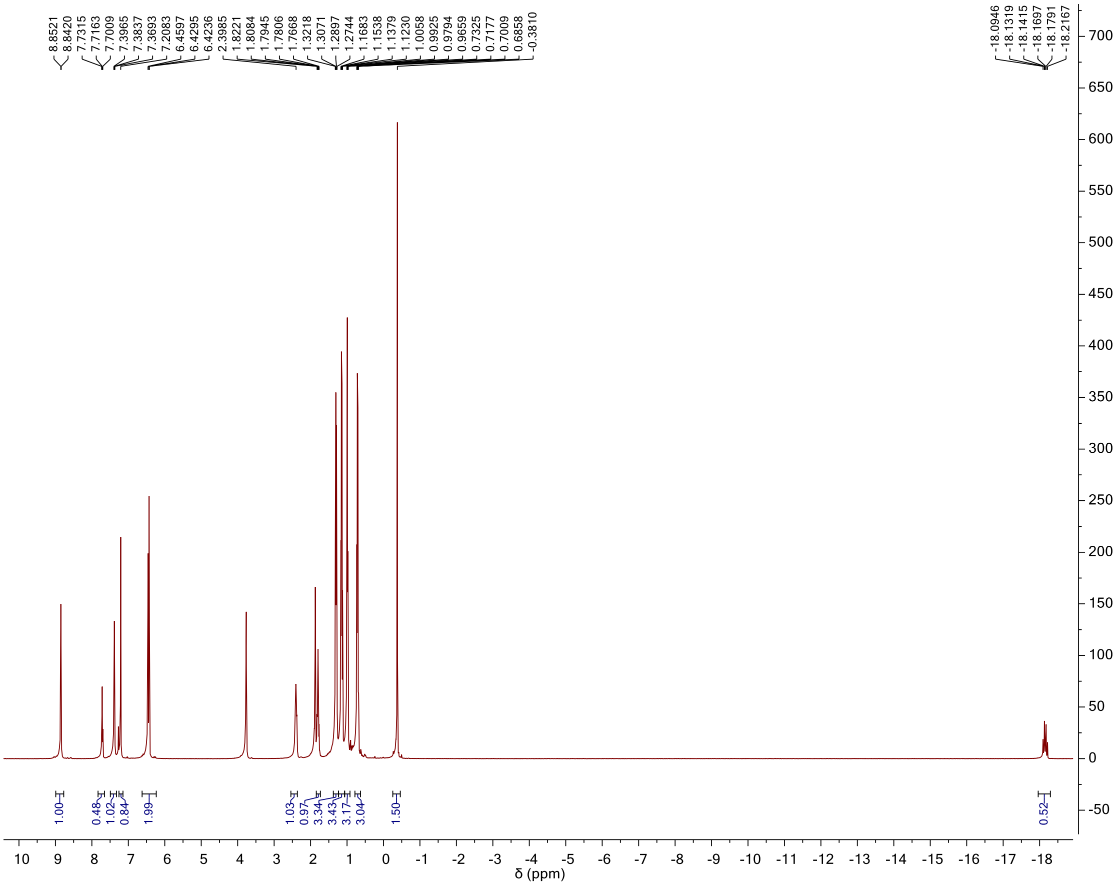

Figure S29. ^{1}H NMR (500 MHz, $CDCl_3$) spectrum of **5-Rh**. Residual THF present.

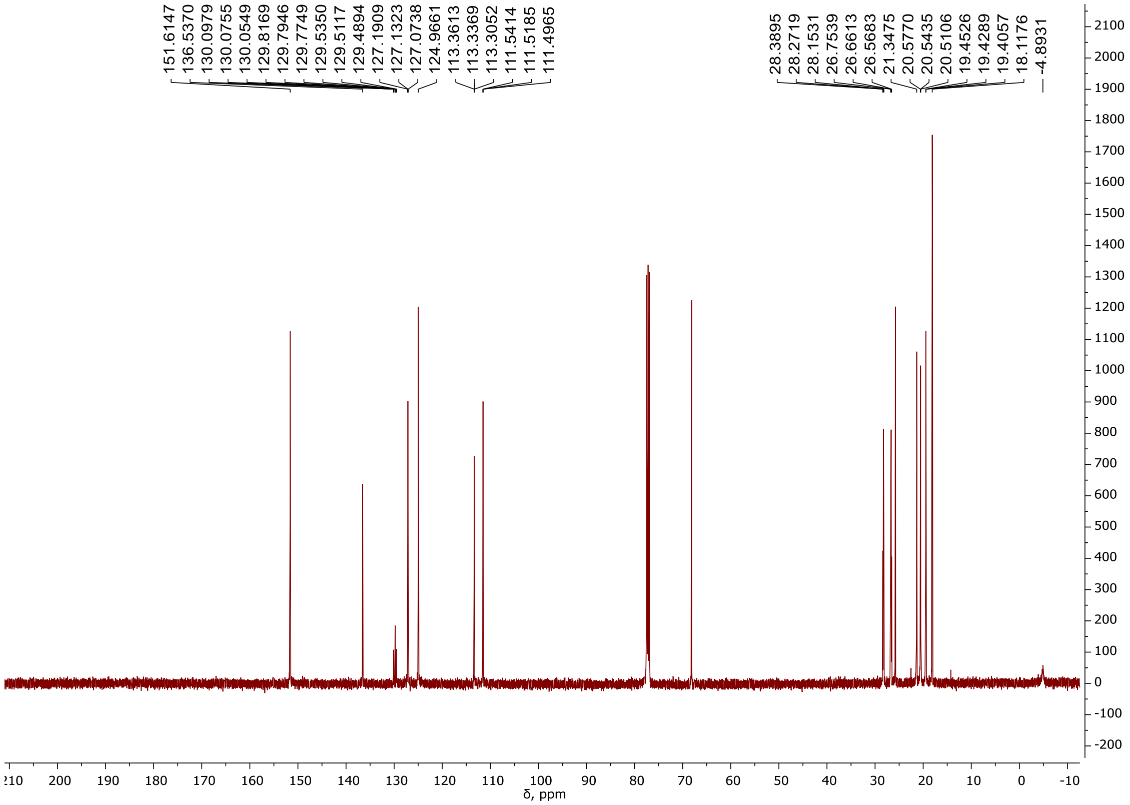

Figure S30. $^{13}C\{^{1}H\}$ NMR (126 MHz, $CDCl_3$) spectrum of **5-Rh**. Residual THF present.

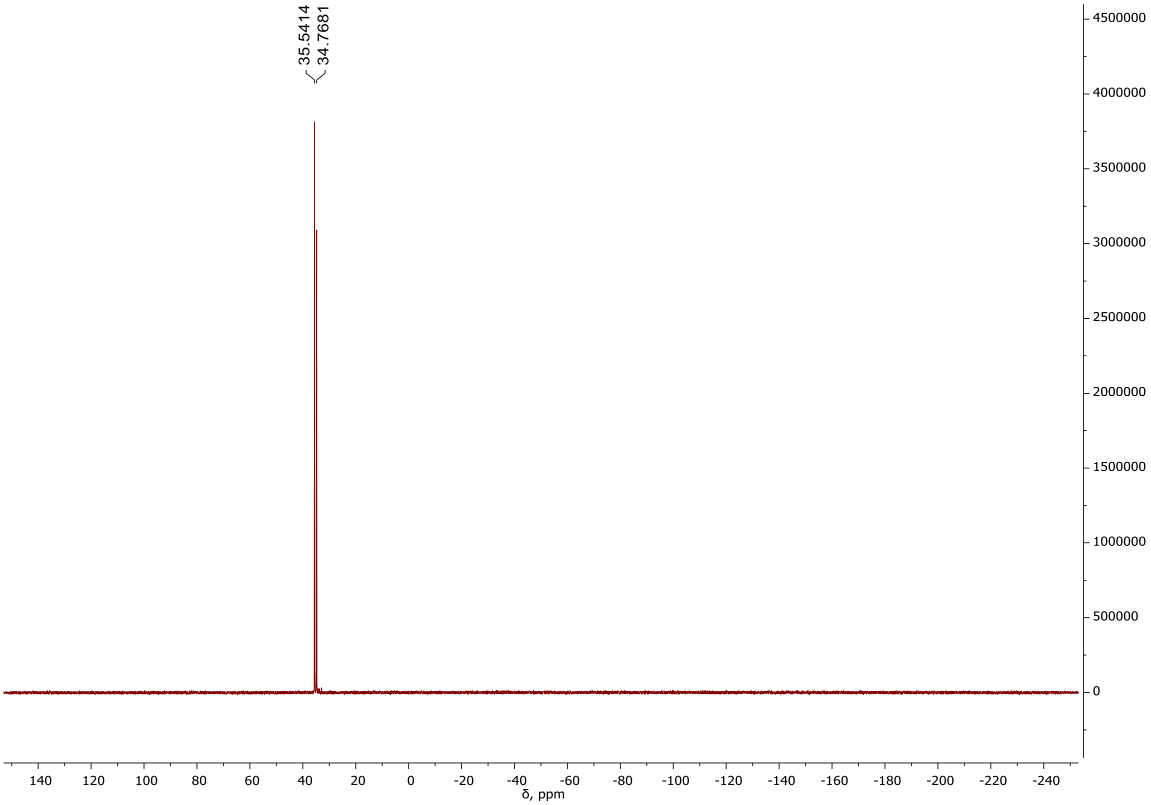

Figure S31. $^{31}P\{^{1}H\}$ NMR (162 MHz, $CDCl_3$) spectrum of **5-Rh**.

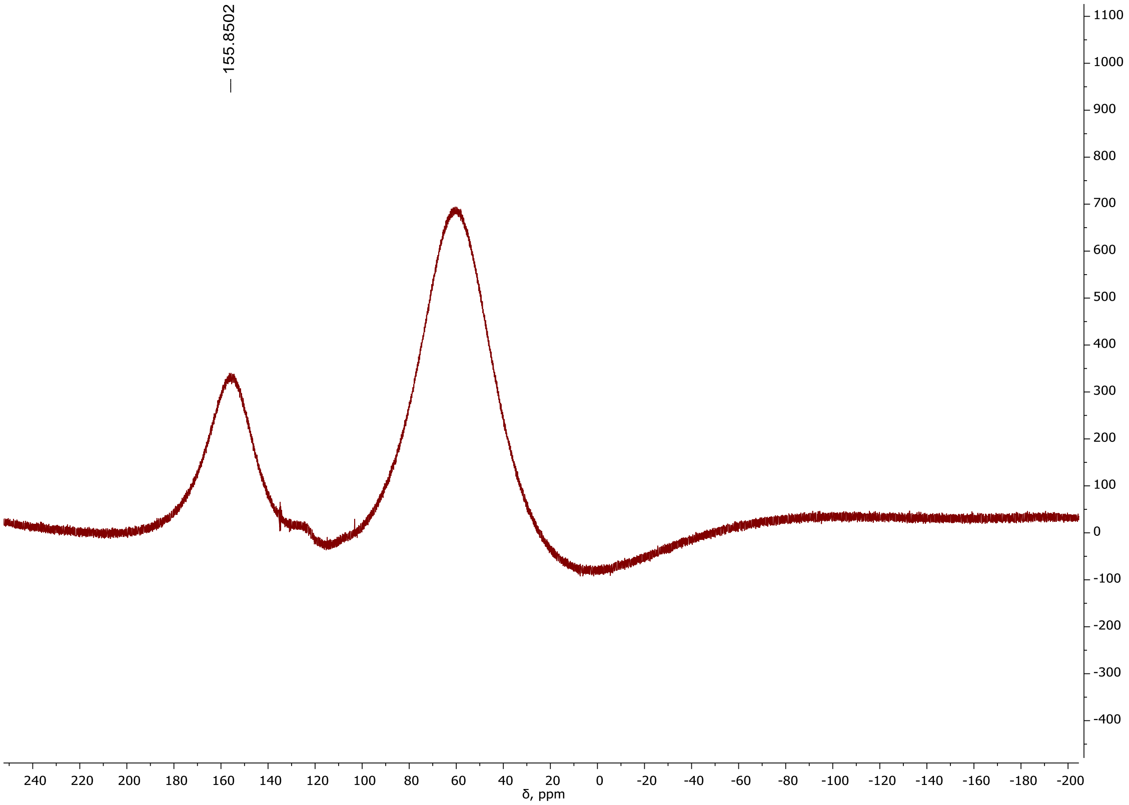

Figure S32. $^{27}Al\{^{1}H\}$ NMR (130 MHz, $CDCl_3$) spectrum of **5-Rh**.

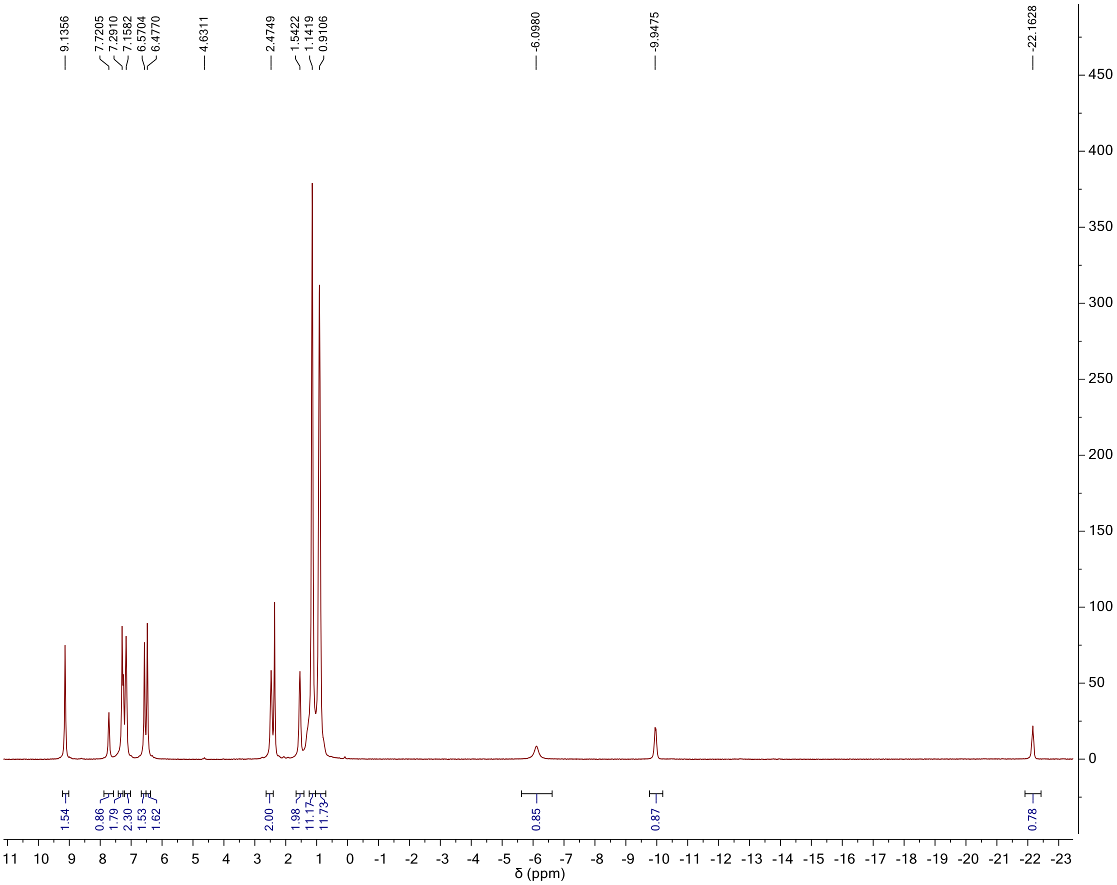

Figure S33. ^{1}H NMR (500 MHz, $CDCl_3$) spectrum of the *in situ* reaction mixture of **4-Ir** under an atmosphere of H_2 for 3 h to form **6-Ir**. Excess H_2 detected.

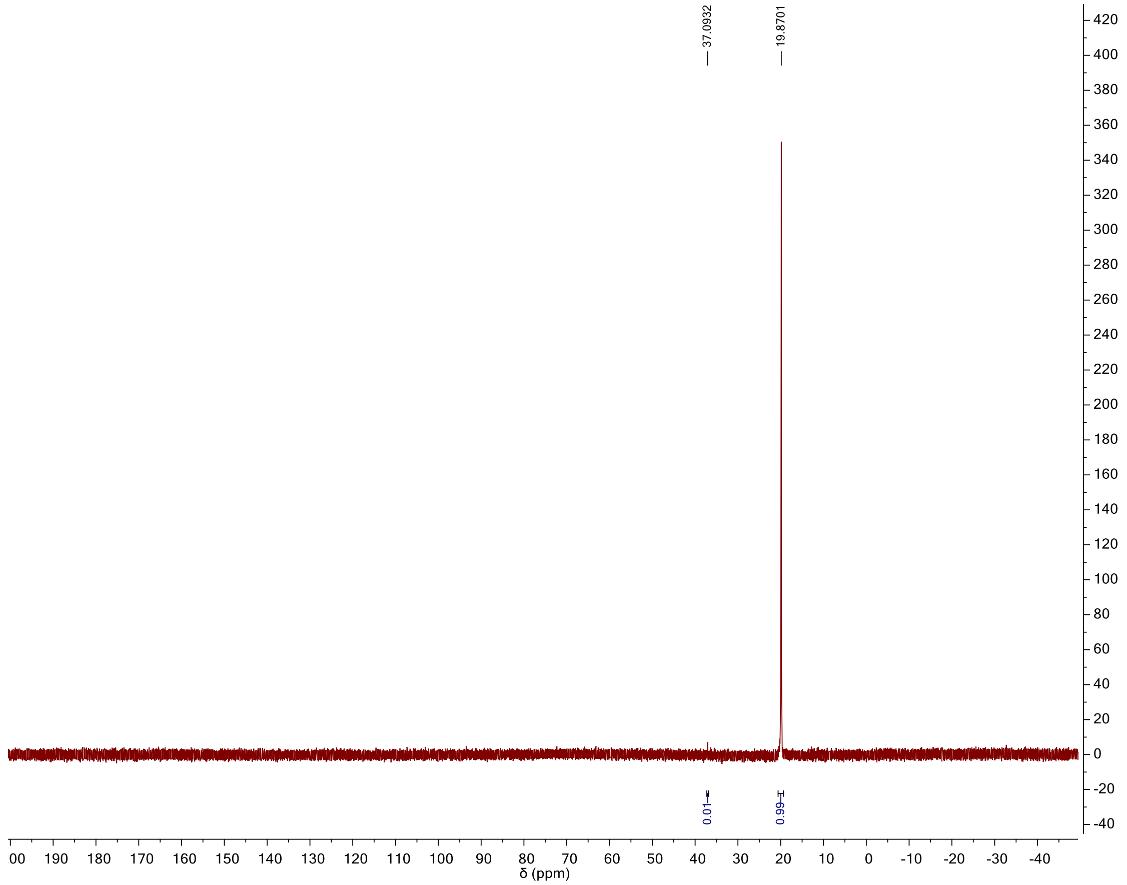

Figure S34. $^{31}P\{^{1}H\}$ NMR (202 MHz, $CDCl_3$) spectrum of the *in situ* reaction mixture of **4-Ir** under an atmosphere of H_2 for 3 h to form **6-Ir**. Unreacted **4-Ir** detected.

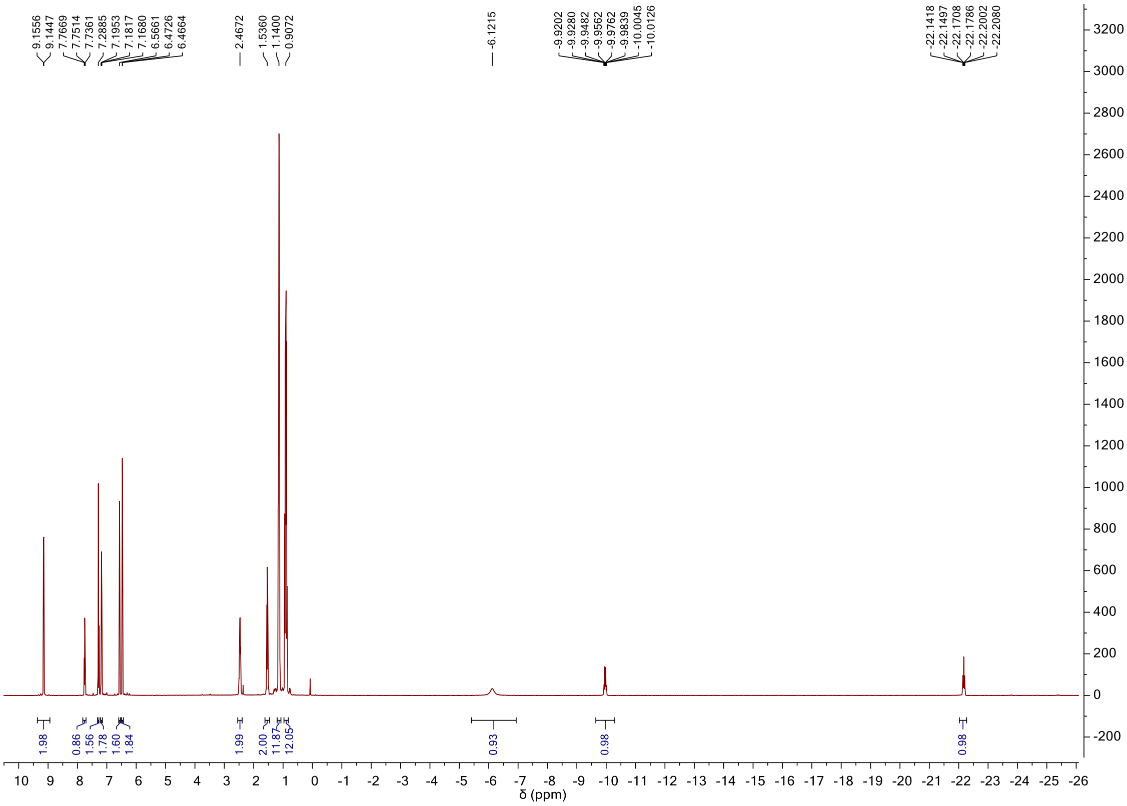

Figure S35. ^{1}H NMR (500 MHz, $CDCl_3$) spectrum of **6-Ir**.

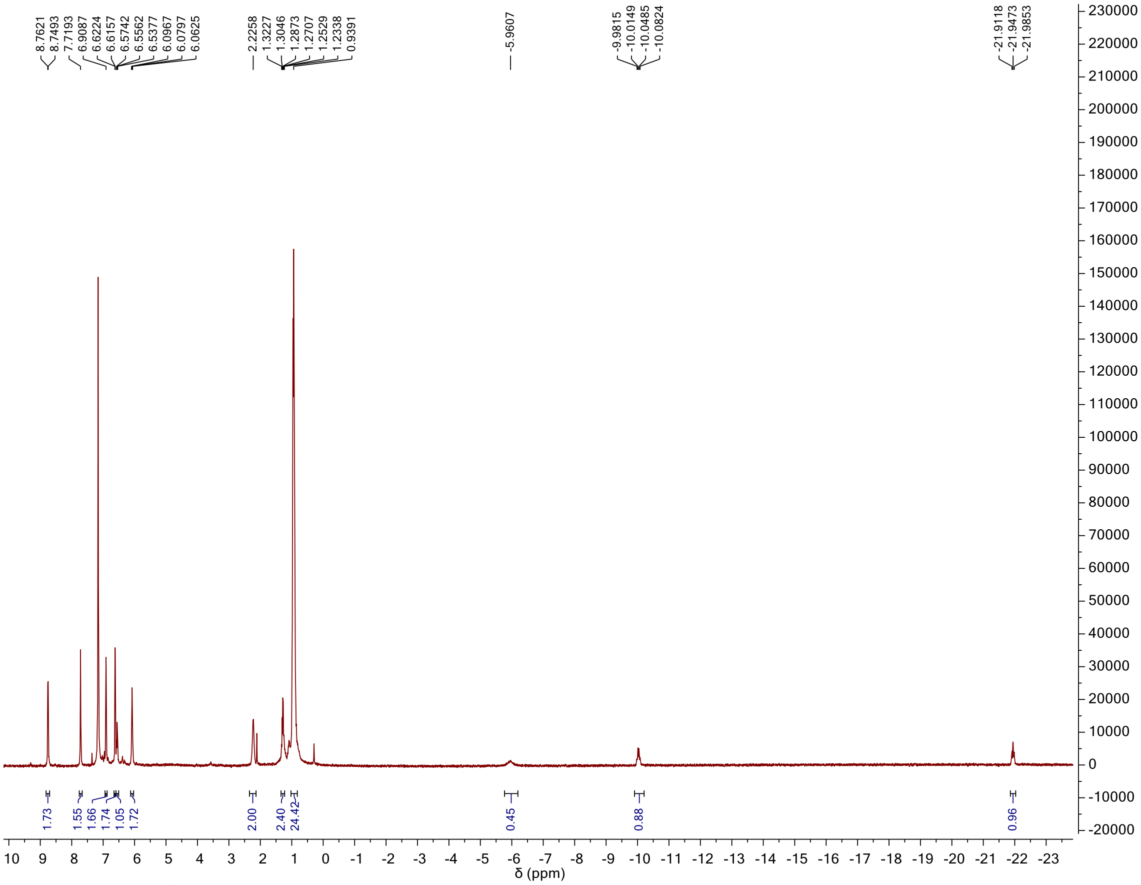

Figure S36. ^{1}H NMR (400 MHz, C_6D_6) spectrum of **6-Ir**.

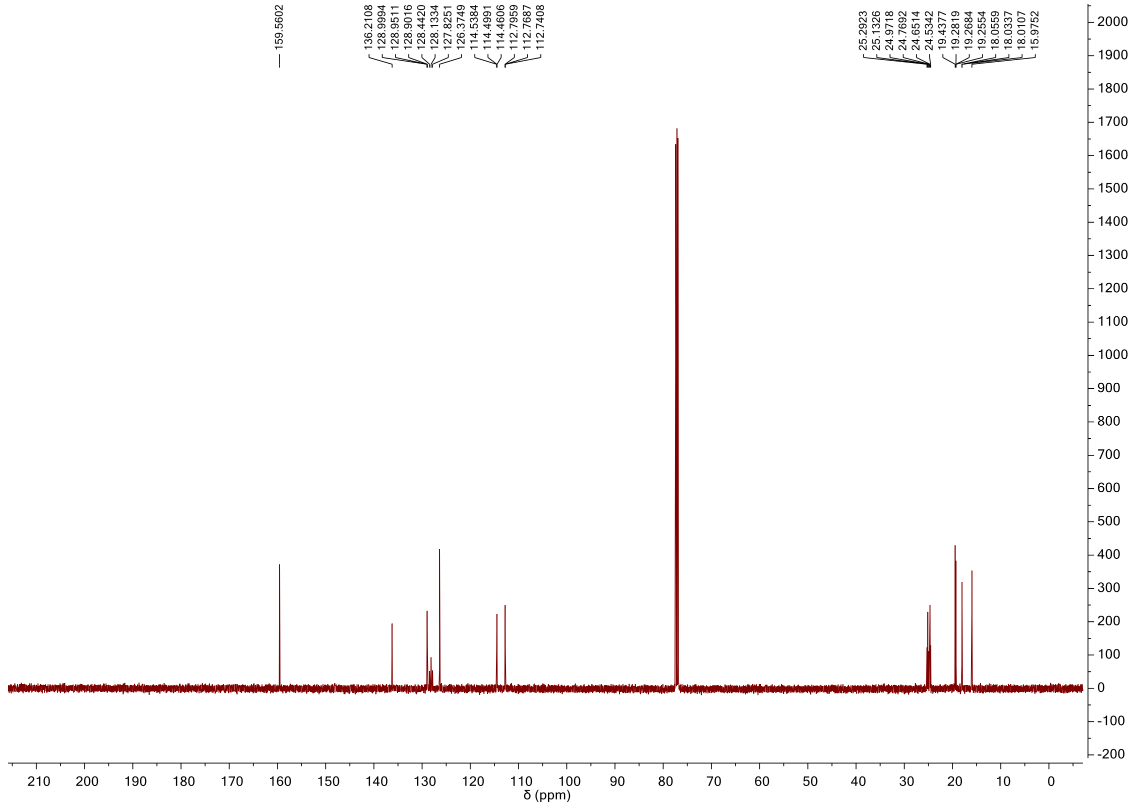

Figure S37. $^{13}C\{^{1}H\}$ NMR (126 MHz, $CDCl_3$) spectrum of **6-Ir**.

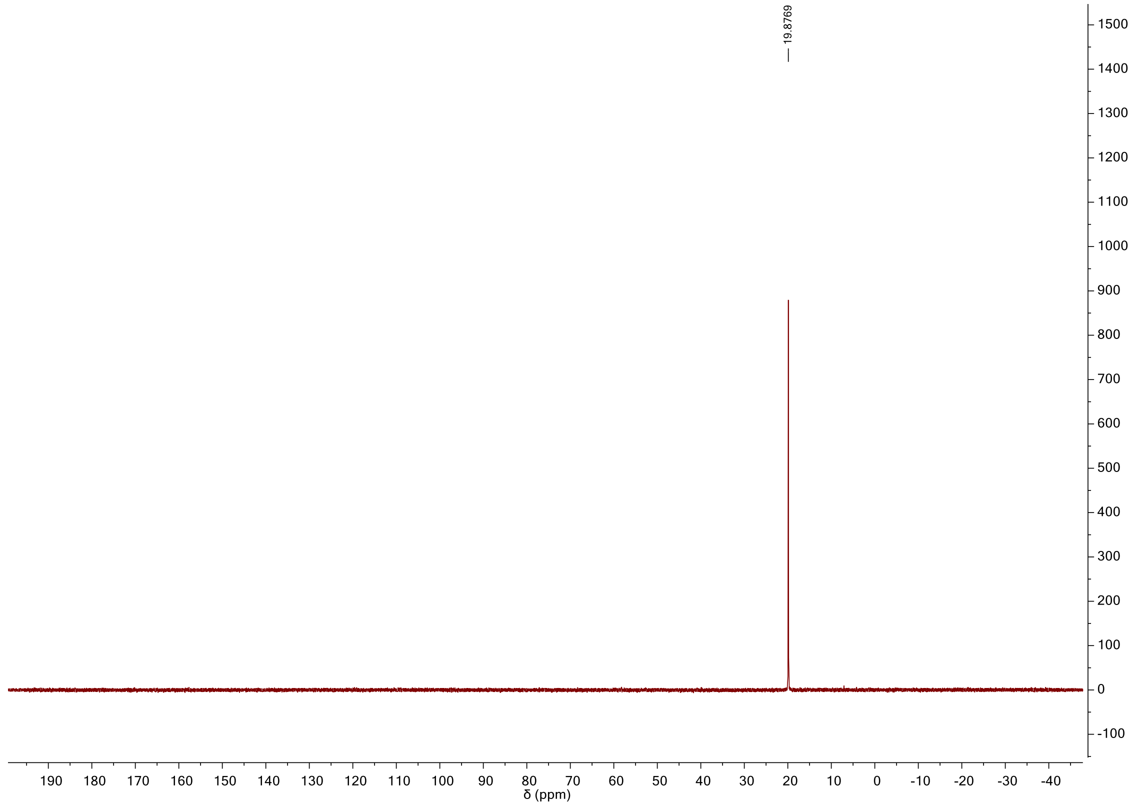

Figure S38. $^{31}P\{^{1}H\}$ NMR (202 MHz, $CDCl_3$) spectrum of **6-Ir**.

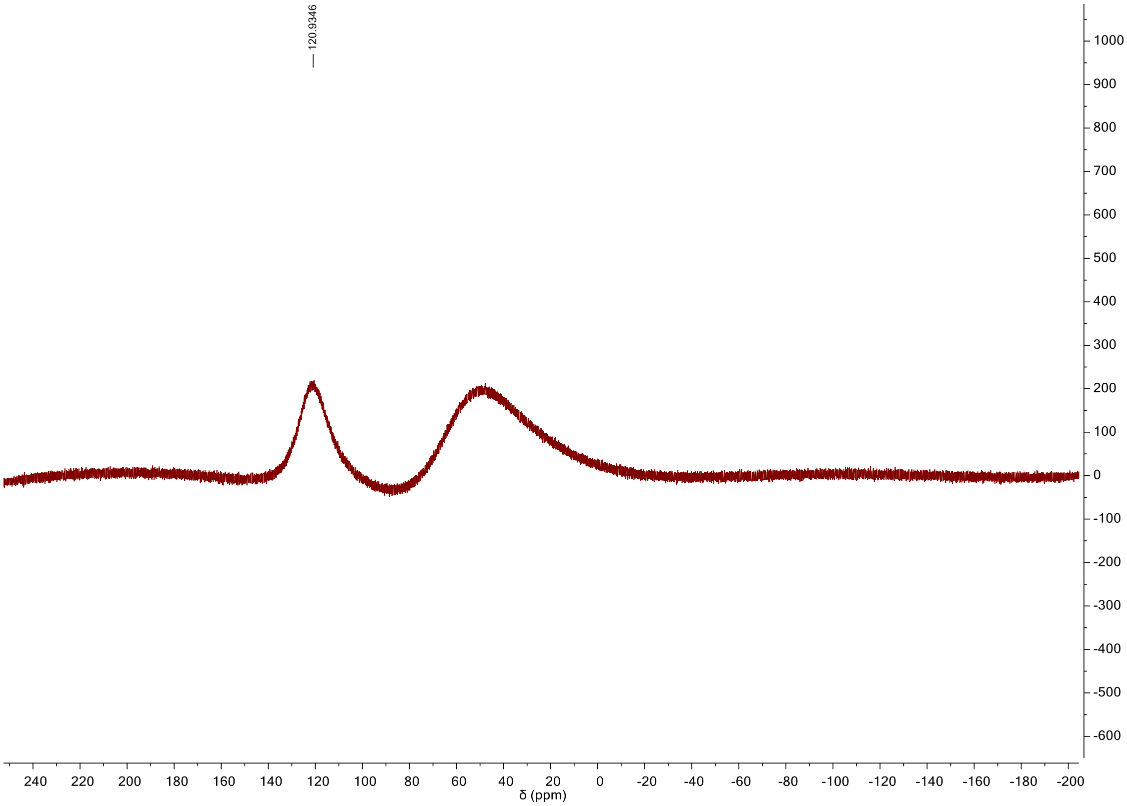

Figure S39. $^{27}Al\{^{1}H\}$ NMR (130 MHz, $CDCl_3$) spectrum of **6-Ir**.

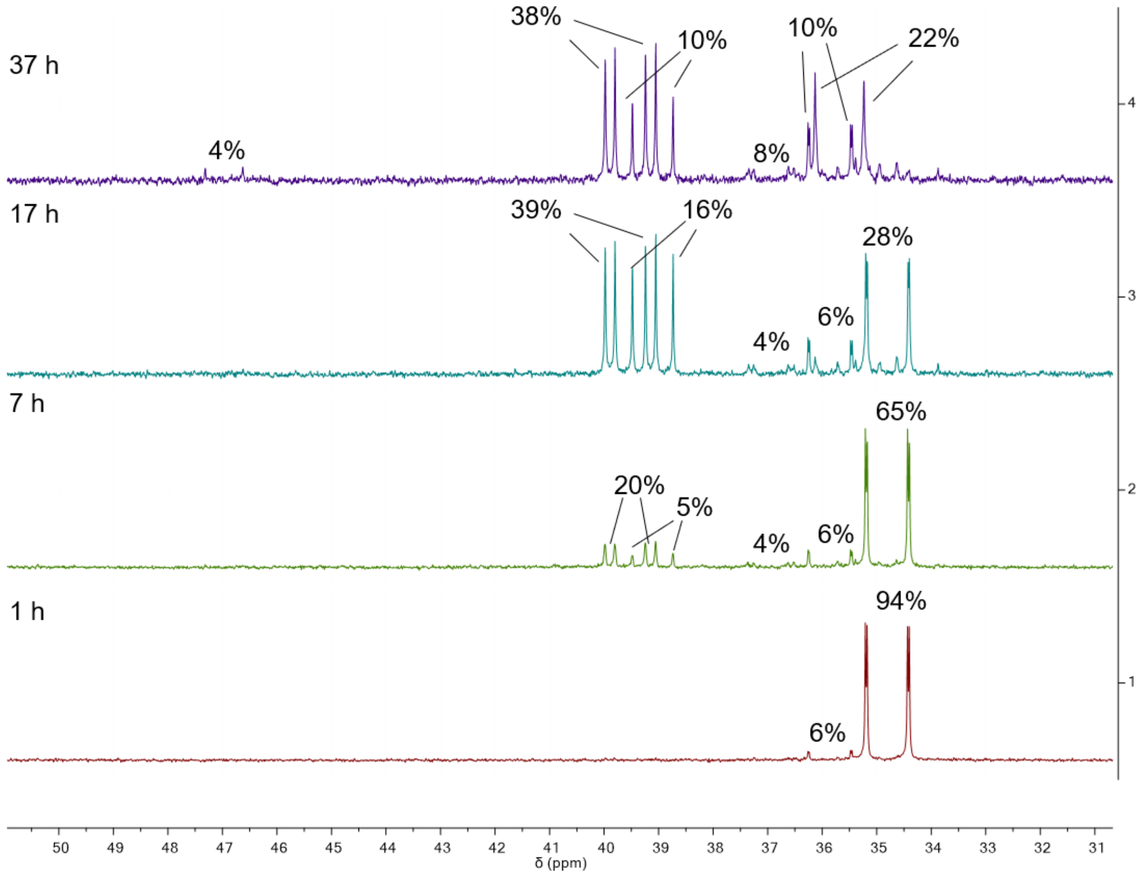

Figure S40. $^{31}P\{^{1}H\}$ NMR (162 MHz, C_6D_6) spectra of the *in situ* reaction mixture of the thermolysis of **5-Rh** at 100 ºC after 1 h, 7 h, 17 h, and 37 h. Smaller peaks with 2%> relative integration were not listed.

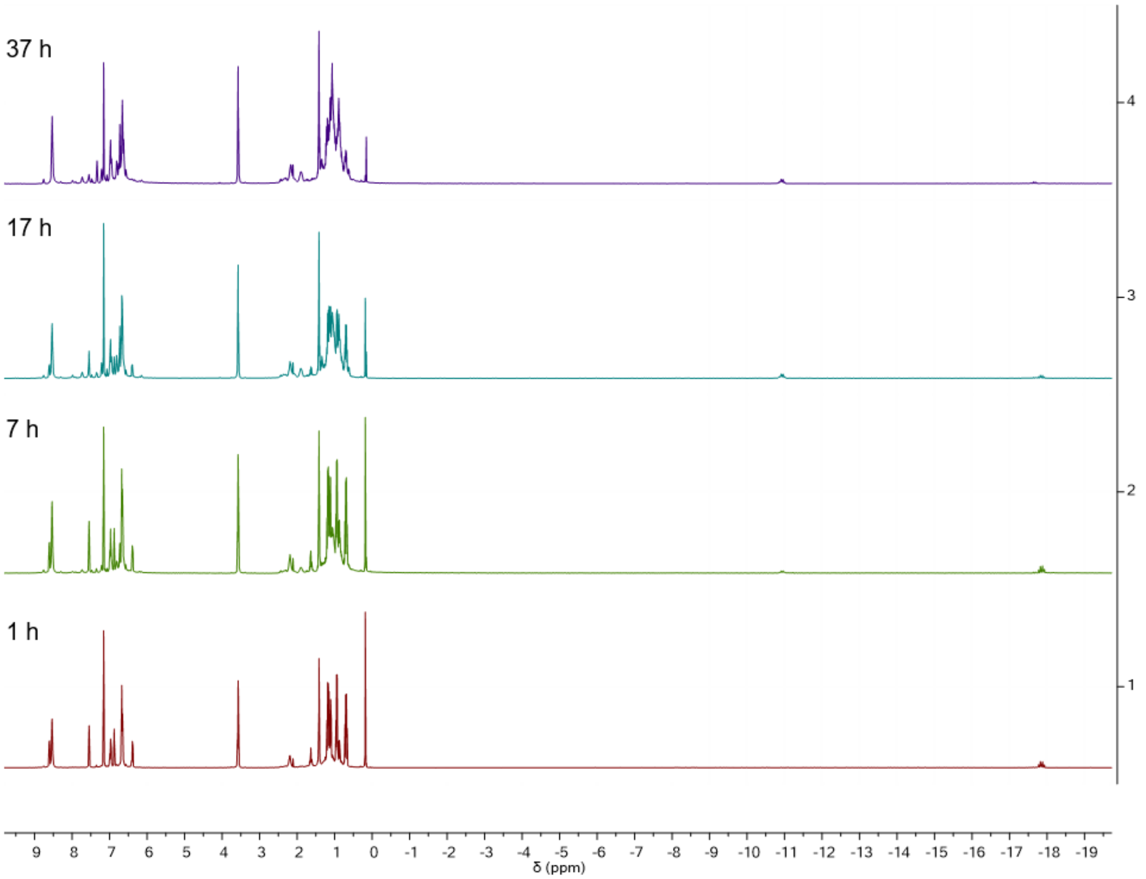

Figure S41. ^{1}H NMR (400 MHz, C_6D_6) spectra of the *in situ* reaction mixture of the thermolysis of **5-Rh** at 100 ºC after 1 h, 7 h, 17 h, and 37 h.

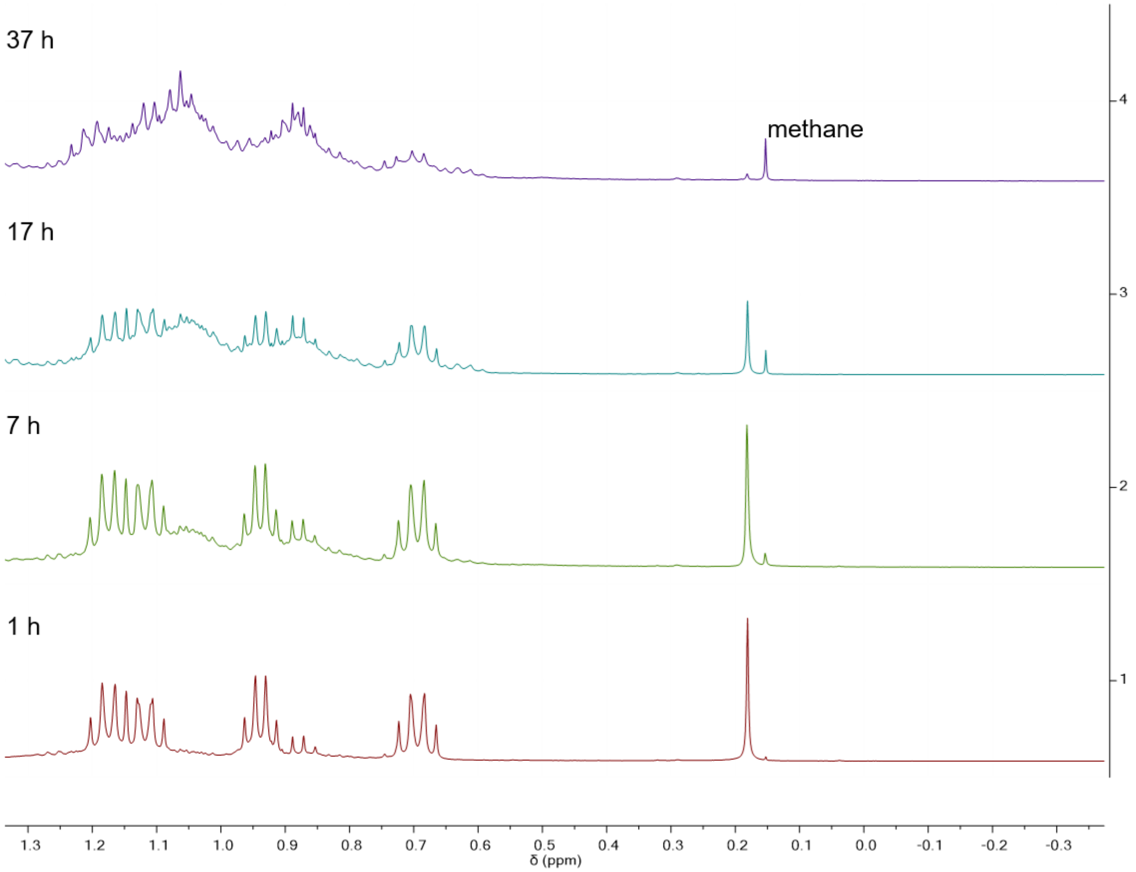

Figure S42. Expanded ^{1}H NMR (162 MHz, C_6D_6) spectra of the *in situ* reaction mixture of the thermolysis of **5-Rh** at 100 ºC after 1 h, 7 h, 17 h, and 37 h.

IV. X-Ray Structural Determination Details

X-Ray data collection, solution, and refinement for 3-Rh (CCDC 2257581). A Leica MZ 75 microscope was used to identify a suitable colorless block with very well defined faces with dimensions (max, intermediate, and min) 0.439 x 0.427 x 0.172 mm^3 from a representative sample of crystals of the same habit. The crystal mounted on a nylon loop was then placed in a cold nitrogen stream (Oxford) maintained at 110 K. A BRUKER Quest X-ray (fixed-Chi geometry) diffractometer with a PHOTON III detector was employed for crystal screening, unit cell determination, and data collection. The goniometer was controlled using the APEX3 software suite.[5] The X-ray radiation employed was generated from a Mo-Iμs X-ray tube (K_α = 0.71073Å). Integrated intensity information for each reflection was obtained by reduction of the data frames with the program APEX3.[5] The absorption correction program SADABS[6] was employed to correct the data for absorption effects. Systematic reflection conditions and statistical tests of the data suggested the space group $P2_1/c$. A solution was obtained readily using XT/XS in APEX3.[5,8] Hydrogen atoms were placed in idealized positions and were set riding on the respective parent atoms. All non-hydrogen atoms were refined with anisotropic thermal parameters. Elongated ellipsoids on the [P2,C15-C20] group suggested disorder and was modeled between two positions with an occupancy ratio of 0.76:0.24. Appropriate restraints and constraints were added to keep the bond distances, angles, and thermal ellipsoids meaningful. Absence of additional symmetry and voids were confirmed using PLATON (ADDSYM).[6] The structure was refined (weighted least squares refinement on F^2) to convergence.[7,8]

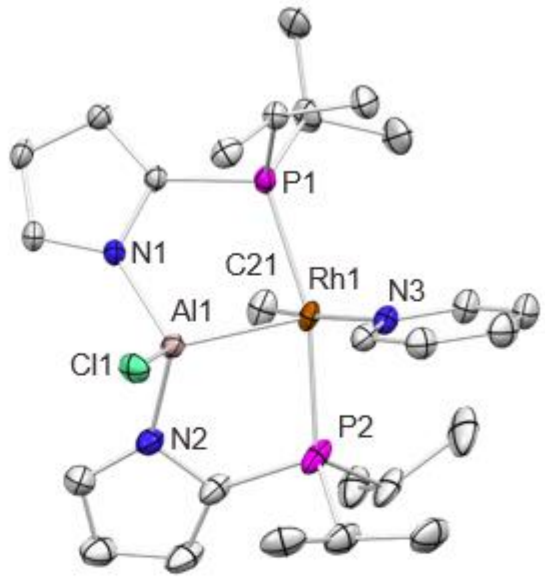

Figure S43. Mercury-rendered ORTEP drawing (50% thermal ellipsoids) of **3-Rh** showing selected atom labeling. Hydrogen atoms were omitted for clarity. Selected bond distances (Å) and angles (°): Rh1-P1, 2.3339(4); Rh1-P2, 2.343(2); Rh1-Al1, 2.3296(5); Rh1-C21, 2.0882(17); Rh1-N3, 2.1561(14); Al1-Cl1, 2.1802(6); Al1-N1, 1.8640(14); Al1-N2, 1.8695(15); P1-Rh1-Al1, 83.894(16); P2-Rh1-Al1, 83.08(4); P1-Rh1-P2, 162.93(4); N3-Rh1-P1, 99.41(4); N3-Rh1-P2, 95.84(7); N3-Rh1-Al1, 113.18(4); N1-Al1-Rh1, 105.39(5); N2-Al1-Rh1, 105.47(5); N1-Al1-Cl1, 103.97(5); N1-Al1-N2, 116.26(7); C21-Rh1-N3, 166.07(6).

X-Ray data collection, solution, and refinement for 3-Ir (CCDC 2257579). A Leica M80 microscope was used to identify a suitable single yellow block-shaped crystal showing well defined faces with dimensions 0.17 × 0.14 × 0.13 mm^3 from a representative sample of crystals of the same habit. The crystal mounted on a nylon loop was then placed in a cold nitrogen stream (Oxford) maintained at T = 110.00 K. Crystal screening, unit cell determination, and data collection were carried out using a Bruker Quest (PHOTON III) diffractometer. The diffraction pattern was indexed and the total number of runs and images was based on the strategy calculation from the program APEX 3 (Bruker, 2018). Data were measured using ϕ and ω scans with MoK_{α} radiation. Data was collected to a maximum resolution of Θ = 27.500° (0.77 Å). The unit cell was refined using SAINT V8.38A (Bruker, 2018) on 9964 reflections, 8 % of the observed reflections. Integrated Intensity information for each reflection was obtained by reduction of data frames using SAINT V8.38A (Bruker, 2018). The final completeness is 99.80 % out to 27.500° in Θ. SADABS-2016/2 (Bruker, 2016/2) was used for absorption correction. wR_2(int) was 0.1157 before and 0.0533 after correction. The Ratio of minimum to maximum transmission is 0.6180. The λ/2 correction factor is Not present. The absorption coefficient μ of this material is 4.354 mm^{-1} at this wavelength (λ = 0.71073Å) and the minimum and maximum transmissions are 0.268 and 0.433. Systematic reflection conditions and statistical tests of the data suggested the space group $P2_1/c$ (#14) and was confirmed by ShelXT 2018/2 (Sheldrick, 2018) structure solution program using dual methods.[7] The structure was refined by full matrix least squares minimization on $\boldsymbol{F^2}$ using version 2018/3 of ShelXL 2018/3 (Sheldrick, 2015).[8] All non-hydrogen atoms were refined anisotropically. Hydrogen atom positions were calculated geometrically and refined using the riding model. The compound crystallized in P21/c. Residual electron density peaks indicated partially occupied solvent sitting on an inversion symmetry position. Accordingly, it was modeled with appropriate restraints. Our trials to reduce the occupancy of the toluene below 0.5 resulted in slightly higher reliability factors. For the final refinement, the occupancy of the solvated toluene was fixed to 0.5. The thermal ellipsoids of partially occupied and disordered solvated toluene still remained elongated possibly suggesting further disorder. No efforts were made to model the latter. SADABS-2016/2 (Bruker, 2016/2) was used for absorption correction.[8] wR_2(int) was 0.1157 before and 0.0533 after correction. The Ratio

of minimum to maximum transmission is 0.6180. The $\lambda/2$ correction factor is Not present. There is a single molecule in the asymmetric unit, which is represented by the reported sum formula. In other words: Z is 4 and Z' is 1.[8]

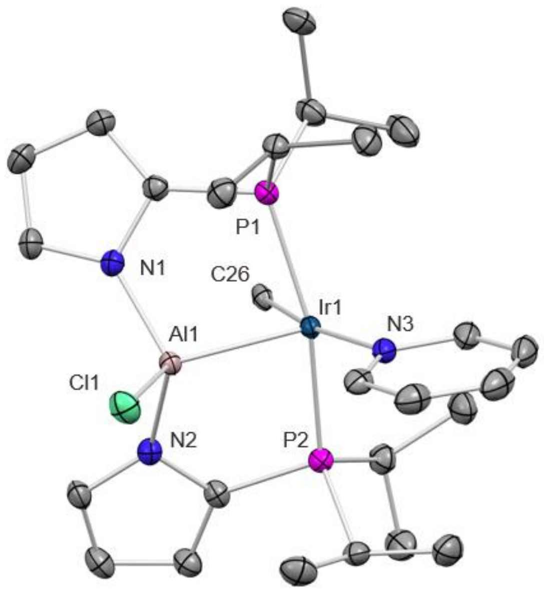

Figure S44. Mercury-rendered ORTEP drawing (50% thermal ellipsoids) of **3-Ir** showing selected atom labeling. Hydrogen atoms and toluene were omitted for clarity. Selected bond distances (Å) and angles (°): Ir1-P1, 2.3143(7); Ir1-P2, 2.3143(7); Ir1-Al1, 2.3505(8); Ir1-N3, 2.142(2); Ir1-C26, 2.1343(7); Al1-N1, 1.872(2); Al1-N2, 1.869(2); Cl1-Al1, 2.1750(11); P2-Ir1-P1, 163.84(3); P1-Ir1-Al1, 84.43(3); P2 Ir1-Al1, 84.76(3); N3-Ir1-P1, 98.74(7); N3-Ir1-P2, 96.25(7); N3-Ir1-Al1, 110.17(7); C26-Ir1-P1, 83.41(8); C26-Ir1-P2, 83.28(7); C26-Ir1-Al1, 82.41(7); C26-Ir1-N3, 167.36(9); Cl1-Al1-Ir1, 123.40(4); N1-Al1-Ir1, 103.51(8); N1-Al1-Cl1, 103.51(8); N1-Al1-N2, 117.4(1).

X-Ray data collection, solution, and refinement for (PAlClP)RhPyH (4-Rh) (CCDC 2257580). A Leica M80 microscope was used to identify a suitable single yellow block-shaped crystal showing well defined faces with dimensions 0.21 × 0.20 × 0.17 mm3 from a representative sample of crystals of the same habit. The crystal mounted on a nylon loop was then placed in a cold nitrogen stream (Oxford) maintained at T = 110.00 K. Crystal screening, unit cell determination, and data collection were carried out using a Bruker Quest (PHOTON III) diffractometer. The diffraction pattern was indexed and the total number of runs and images was based on the strategy calculation from the program APEX 3 (Bruker, 2018). Data were measured using ϕ and ω scans with MoK_{α} radiation. Data was collected to a maximum resolution of Θ = 27.538° (0.77 Å). The unit cell was refined using SAINT V8.38A (Bruker, 2018) on 9969 reflections, 5% of the observed reflections. Integrated Intensity information for each reflection was obtained by reduction of data frames using SAINT V8.38A (Bruker, 2018). The final completeness is 99.70 % out to 27.538° in Θ. SADABS-2016/2 (Bruker, 2016/2) was used for absorption correction. wR_2(int) was 0.1280 before and 0.0750 after correction. The Ratio of minimum to maximum transmission is 0.8615. The λ/2 correction factor is Not present. The absorption coefficient μ of this material is 0.722 mm^{-1} at this wavelength (λ = 0.71073Å) and the minimum and maximum transmissions are 0.285 and 0.331. Systematic reflection conditions and statistical tests of the data suggested the space group $P2_1/n$ (# 14) and was confirmed by ShelXT 2018/2 (Sheldrick, 2018) structure solution program using dual methods. The structure was refined by full matrix least squares minimisation on $\boldsymbol{F}^2$ using version 2018/3 of XL (Sheldrick, 2008).[7] All non-hydrogen atoms were refined anisotropically. Hydrogen atom positions were calculated geometrically and refined using the riding model. The compound crystallized in the monoclinic P21/n space group. Elongated thermal ellipsoids and nearby residual electron density peaks near the isopropyl groups (C20, C21, C22) and (C23, C24, C25) suggested disorder which were modeled between two positions each with an occupancy ratio of 0.76. Slightly elongated thermal ellipsoids on the other two isopropyl groups suggested minor disorder. No efforts were made to model the latter. Residual electron density peaks suggested presence of disordered solvent(s). We could model disordered C_6D_6 at two locations. Our trials indicated the occupancy of both the C_6D_6 sites were very close to 0.5. For the final least squares

refinement cycles, the occupancies were fixed to 0.5. Appropriate restraints were added to keep the bond distances, angles, and thermal ellipsoids of disordered groups meaningful. SADABS-2016/2 (Bruker,2016/2) was used for absorption correction. wR_2(int) was 0.1280 before and 0.0750 after correction. The ratio of minimum to maximum transmission is 0.8615. The $\lambda/2$ correction factor is not present. There is a single molecule in the asymmetric unit, which is represented by the reported sum formula. In other words: Z is 4 and Z' is 1.[8]

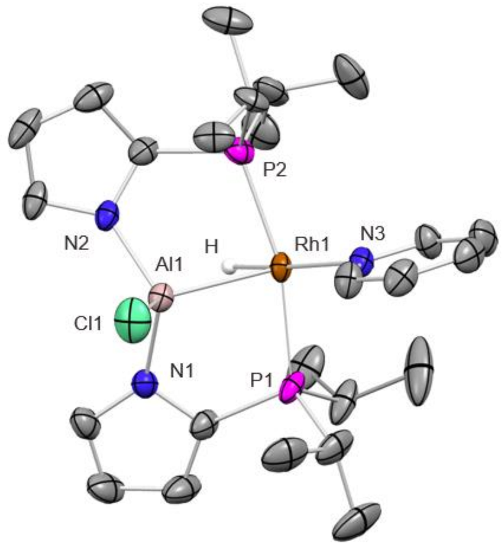

Figure S45. Mercury-rendered ORTEP drawing (50% thermal ellipsoids) of **4-Rh** showing selected atom labeling. Non-hydride hydrogen atoms and C_6D_6 were omitted for clarity. Selected bond distances (Å) and angles (°): Rh1-P2, 2.2933(6); Rh1-P1, 2.2983(6); Rh1-Al1, 2.3407(6); Rh1-N3, 2.1473(16); Al1-Cl1, 2.1564(8); Al1-N1, 1.8760(18); Al1-N2, 1.8746(18); P2-Rh1-Al1, 84.34(2); P1-Rh1-P2, 161.193(19); P1-Rh1-Al1, 83.79(2); N3-Rh1-P2, 100.66(5); N3-Rh1-P1, 98.03(5); N3-Rh1-Al1, 122.81(5); N1-Al1-Rh1, 105.44(6); N1-Al1-Cl1, 105.22(6); N1-Al1-N2, 119.25(8); Cl1-Al1-Rh1, 117.00(3).

X-Ray data collection, solution, and refinement for 5-Rh (CCDC 2277405). A Leica M80 microscope was used to identify a suitable single yellow block-shaped crystal of **5-Rh** showing well defined faces with dimensions 0.24 x 0.13 x 0.13 mm^3 from a representative sample of crystals of the same habit. The crystal mounted on a nylon loop was then placed in a cold nitrogen stream (Oxford) maintained at T = 110.00 K. Crystal screening, unit cell determination, and data collection were carried out using a Bruker Quest (PHOTON III) diffractometer. The diffraction pattern was indexed and the total number of runs and images was based on the strategy calculation from the program APEX 4 (Bruker, 2022). Data were measured using *f* and *w* scans with MoK_a radiation. Data was collected to a maximum resolution of $Q = 27.521^{\circ}$ (0.77 Å). The unit cell was refined using SAINT V8.38A (Bruker, 2018) on 9793 reflections, 12 % of the observed reflections. Integrated intensity information for each reflection was obtained by reduction of data frames using SAINT V8.38A (Bruker, 2018). The final completeness is 99.80 % out to 27.521° in Q. SADABS-2016/2 (Bruker, 2016/2) was used for absorption correction. wR_2(int) was 0.0746 before and 0.0595 after correction. The Ratio of minimum to maximum transmission is 0.9013. The $l/2$ correction factor is not present. The absorption coefficient m of this material is 0.640 mm^{-1} at this wavelength (l = 0.71073Å) and the minimum and maximum transmissions are 0.388 and 0.430. Systematic reflection conditions and statistical tests of the data suggested the space group $P2_1/n$ (# 14) and was confirmed by ShelXT 2018/2 (Sheldrick, 2018) structure solution program using dual methods. The structure was refined by full matrix least squares minimisation on $\boldsymbol{F^2}$ using version 2018/3 of ShelXL 2018/3 (Sheldrick, 2015).[8] All non-hydrogen atoms were refined anisotropically. Hydrogen atom positions were calculated geometrically and refined using the riding model. THF and toluene were used for synthesis and crystallization. Partially occupied and disordered solvents were present which could not be successfully modeled. Although we could see the presence of THF, its disorder with another solvent could not be clearly modeled. For the final least squares refinement cycles, the solvent molecules were MASKed using Olex2, accounting for 308 electrons in the unit cell. No efforts were taken to find the exact solvent composition. Elongated ellipsoids and residual electron density peaks near C20 - C25 suggested disorder, which was modeled between two positions with an occupancy ratio of 0.70. Appropriate restraints and constraints were added to keep the

bond distances, angles, and thermal ellipsoids meaningful. Elongated ellipsoids on other atoms indicated further minor disorder. No efforts were taken to model any further disorder. There is a single molecule in the asymmetric unit, which is represented by the reported sum formula. In other words: Z is 4 and Z' is 1.

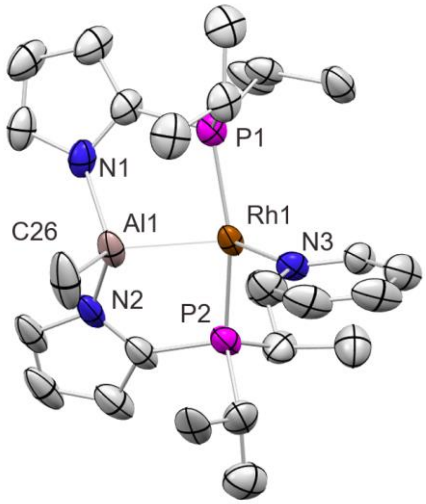

Figure S46. Mercury-rendered ORTEP drawing (50% thermal ellipsoids) of **5-Rh** showing selected atom labeling. Hydrogen atoms and C_6D_6 solvent were omitted for clarity. Selected bond distances (Å) and angles (°): Rh1-P1, 2.2904(12); Rh1-P2, 2.2872(12); Rh1-Al1, 2.3754(12); Rh1-N3, 2.156(3); Al1-N1, 1.907(4); Al1-N2, 1.901(4); Al1-C26, 1.976(5); P2-Rh1-P1, 160.09(4); N3-Rh1-Al1, 121.43(10); P1-Rh1-Al1, 83.95(5); P2-Rh1-P1, 160.09(4); P2-Rh1-Al1, 85.10(5); N1-Al1-Rh1, 103.27(11); N1-Al1-N2, 115.55(16); N1-Al1-C26, 107.0(3); C26-Al1-Rh1, 120.74(16).

X-Ray data collection, solution, and refinement for 6-Ir (CCDC 2269356). A Leica MZ 75 microscope was used to identify a light-yellow block of suitable size with very well defined faces with dimensions (max, intermediate, and min) 0.26 x 0.28 x 0.32 mm^3 from a representative sample of crystals of the same habit. The crystal mounted on a nylon loop was then placed in a cold nitrogen stream (Oxford) maintained at 110 K. X-ray data were obtained on a Bruker APEXII CCD based diffractometer (Mo sealed X-ray tube, K_α = 0.71073 Å). All diffractometer manipulations, including data collection, integration and scaling were carried out using the Bruker APEX3 software.[6] The absorption correction program SADABS[7] was employed to correct the data for absorption effects. The space group was determined on the basis of systematic absences and intensity statistics and the structure was solved by direct methods and refined by full-matrix least squares on F^2. The structure was solved in the orthorhombic $P2_1/n$ space group using XS[8] (incorporated in SHELXLE/OLEX2). All non-hydrogen atoms were refined with anisotropic thermal parameters. All hydrogen atoms were placed in idealized positions and refined using riding model. The structure was refined (weighted least squares refinement on F^2) and the final least-squares refinement converged.[7] No additional symmetry was found using ADDSYM incorporated in PLATON program.[6]

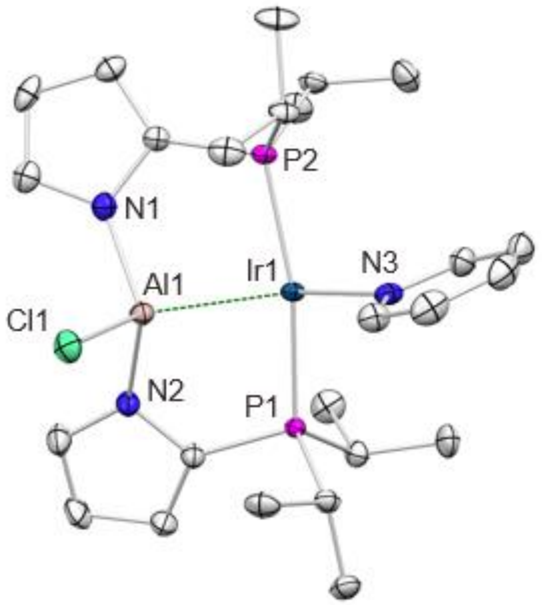

Figure S47. Mercury-rendered ORTEP drawing (50% thermal ellipsoids) of **6-Ir** showing selected atom labeling. Hydrogen atoms and C_6D_6 solvent were omitted for clarity.

Selected bond distances (Å) and angles (°): Ir1-P1, 2.3112(7); Ir1-P2, 2.3073(8); Ir1-Al1, 2.5104(9); Ir1-N3, 2.176(3); Cl1-Al1, 2.1669(12); Al1-N1, 1.881(3); Al1-N2, 1.890(3); P1-Ir1-Al1, 85.23(3); P2-Ir1-P1, 164.84(3); P2-Ir1-Al1, 85.66(3); N3-Ir1-P1, 99.63(7); N3-Ir1-P2, 95.53(7); N3-Ir1-Al1, 128.29(7); N1-Al1-Ir1, 98.81(9); N1-Al1-Cl1, 102.49(9); N1-Al1-N2, 124.39(13); Cl1-Al1-Ir1, 132.99(5); Al1-Ir1-N3, 128.29(7).

VI. DFT Computational Studies

Computational Details

Gaussian16 [9] was used for all electronic structure calculations. Geometry optimization in gas-phase at T = 298.15 K were calculated using M06[10] functional with SDD basis set with effective core potential[11] for Ir and Rh and 6-311G(d,p)[12,13,14] basis set for other atoms. Frequency calculation of the gas-phase optimized structures was carried out to obtain thermodynamic properties, and to verify the optimized structure as intermediate or transition state. Solvent-corrected free energies were calculated using the M06 functional with SDD basis set with effective core potential for Ir and Rh, and 6-311+G(d,p)[12-15,16]basis set for other atoms on the gas-phase optimized structure. The SMD[17] continuum solvation model was used with the solvent parameters for toluene (ε = 2.3741). Intrinsic reaction coordinate calculations (IRC)[18] were performed on all transition states to verify that all transition states connect to their relevant intermediates along the reaction coordinate. Jimp2[19,20] software was used to visualize and draw all optimized molecular geometries. Second-order perturbation energy analysis and natural localized molecular orbital (NLMO) populations were performed using NBO 6.0.[21]

Table S5. Selected bond distances in the X-ray crystal structures and the M06 optimized structures of **3-Rh** and **4-Rh**.

Bond distance (Å)	3-Rh		4-Rh	
	X-ray	M06	X-ray	M06
**Rh-Al**	2.3296(5)	2.383	2.3407(6)	2.381
**Rh-Me(trans-to-py)**	2.0882(17)	2.108	-	-
**Rh-H(trans-to-py)**	-	-	1.5259	1.585
**Rh-N(py)**	2.1561(14)	2.203	2.1473(16)	2.193
**Rh-P1**	2.3339(4)	2.367	2.2983(6)	2.343
**Rh-P2**	2.343(2)	2.371	2.2933(6)	2.343
**MUE**		0.036		0.046

Table S6. Selected bond distances in the X-ray crystal structures and the M06 optimized structures of **6-Ir**.

Bond distance (Å)	6-Ir	
	X-ray	M06
**Ir-Al**	2.5104(9)	2.615
**Ir-N(py)**	2.176(3)	2.251
**Ir-P1**	2.3112(7)	2.354
**Ir-P2**	2.3073(8)	2.355
**Ir-H(bridge)**	-	1.737
**Al-H(bridge)**	-	1.756
**Ir-H(trans-to-py)**	-	1.589
**Ir-H(cis-to-py)**	-	1.631
**MUE**		0.068

Table S7. Second-order perturbation energy ($\Delta E^{(2)}$) (kcal mol^{-1}) from the donor orbital to the acceptor orbital of **4-Rh**, **4-Ir**, **6-Rh**, and **6-Ir**.

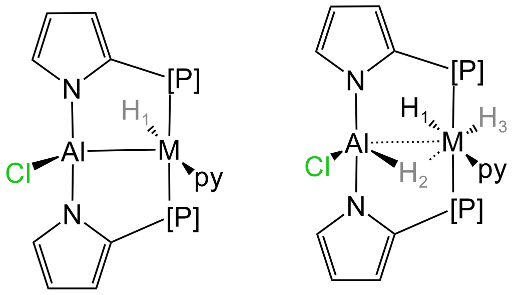

4 **6**

	donor	acceptor	**4-Rh**	**4-Ir**	**6-Rh**	**6-Ir**
$\Delta E_1^{(2)}$	LP(M)	LV(Al)	77.6	271.5	-	-
$\Delta E_2^{(2)}$	σ(M-H1)	LV(Al)	31.6	15.4	12.3	22.6
$\Delta E_3^{(2)}$	σ(Al-H2)	LV(M)	-	-	94.8	272.4

*M = Rh, Ir

Table S8. The natural localized molecular orbital (NLMO) populations of **4-Rh**, **4-Ir**, **6-Rh**, and **6-Ir**.

	**4-Rh**	**4-Ir**	**6-Rh**	**6-Ir**
LP(M)				
M	88.2%	84.3%	-	-
H1	0.1%	0.1%	-	-
Al	10.3%	13.9%	-	-
σ(M-H1)				
M	52.3%	54.7%	63.1%	58.8%
H1	39.3%	39.2%	32.2%	35.5%
Al	6.1%	3.2%	2.1%	3.0%
σ(Al-H2)				
M	-	-	5.9%	7.9%
H2	-	-	61.2%	58.7%
Al	-	-	20.5%	19.3%

*M = Rh, Ir

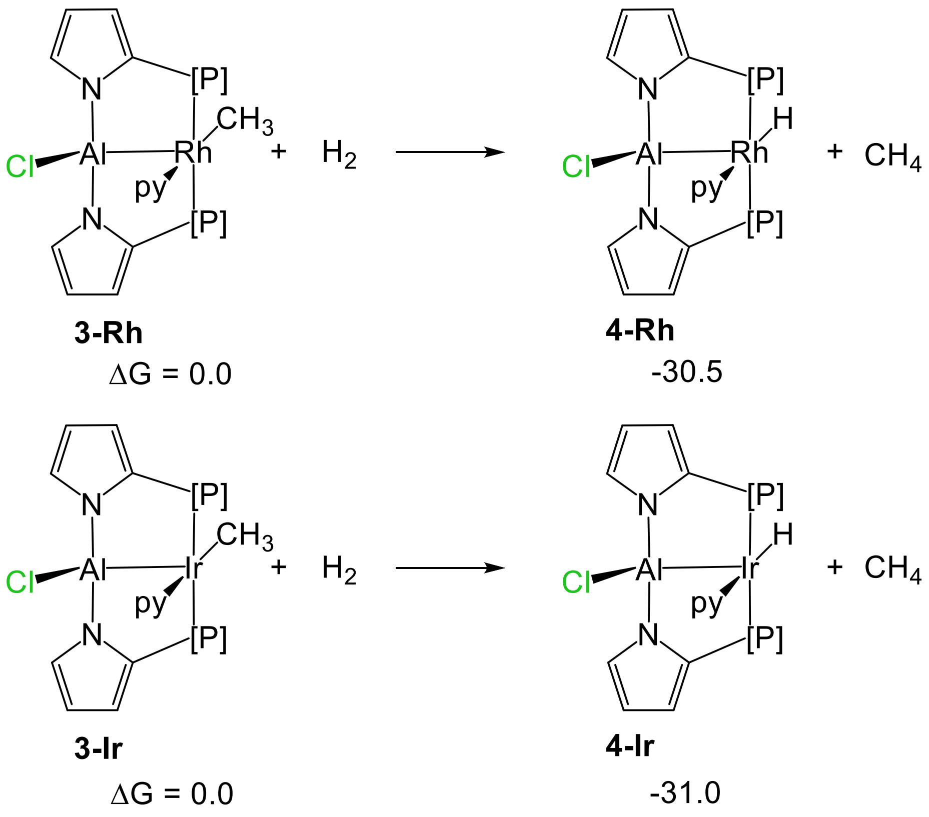

Figure S48. Solvent-corrected relative free energies (in kcal/mol) for the hydrogenolysis of **3-Rh** and **3-Ir**.

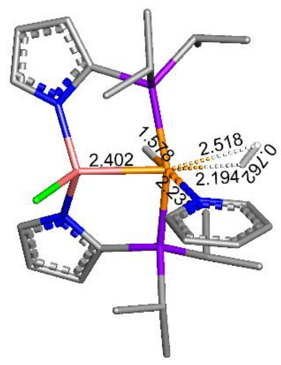

TS1-Rh

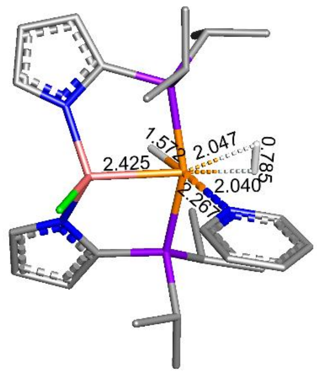

INT1-Rh

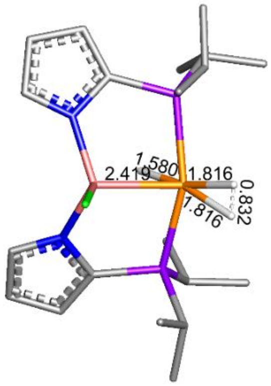

INT2-Rh

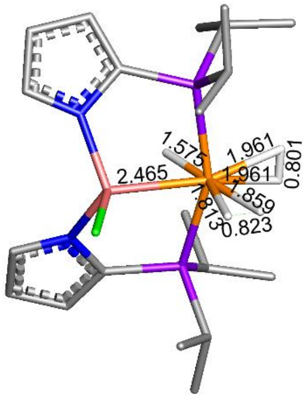

INT3-Rh

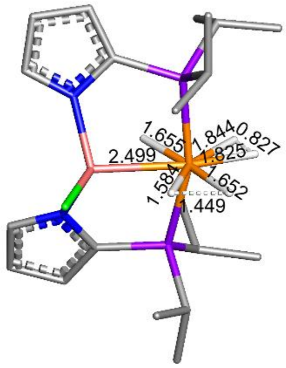

TS2-Rh

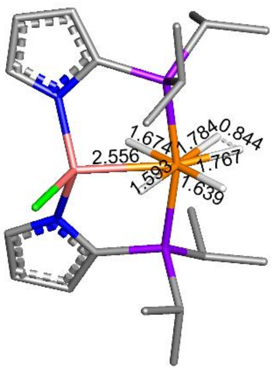

INT4-Rh

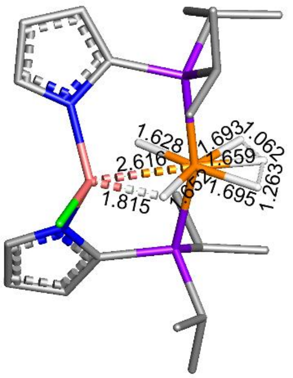

TS3-Rh

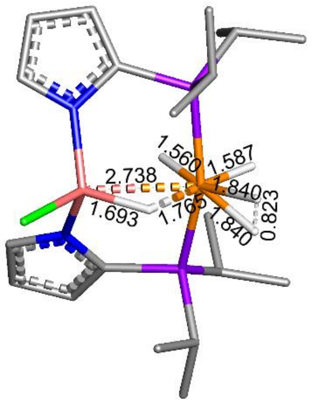

INT5-Rh

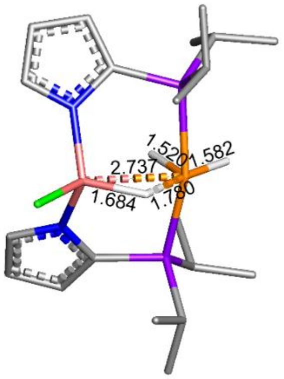

INT6-Rh

Figure S49. Optimized geometries of intermediates and transition states in the H_2 activation by **4-Rh.** Rh is shown in orange, P in purple, Al in brick red, N in blue, Cl in lime green, C in gray, and H in white. Hydrogen atoms were omitted for clarity except for those on the Rh.

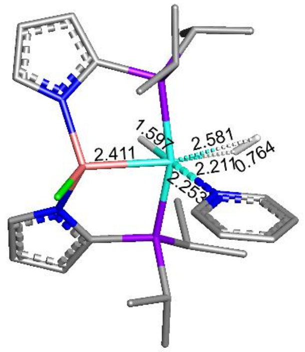

TS1-Ir

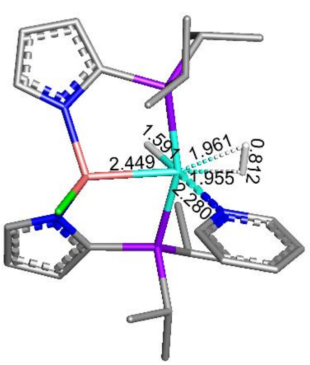

INT1-Ir

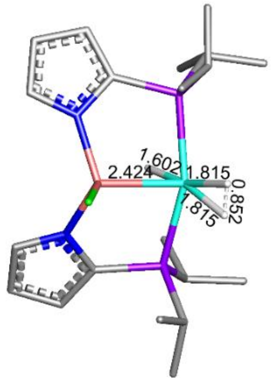

INT2-Ir

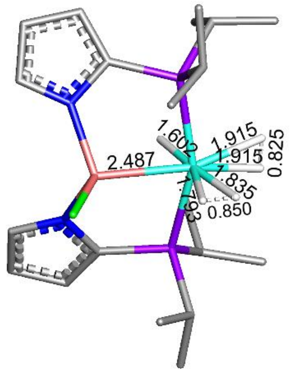

INT3-Ir

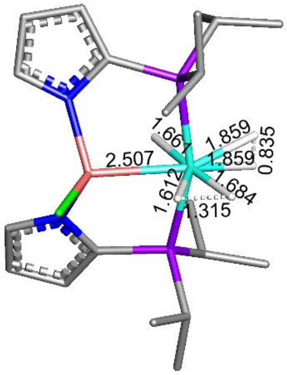

TS2-Ir

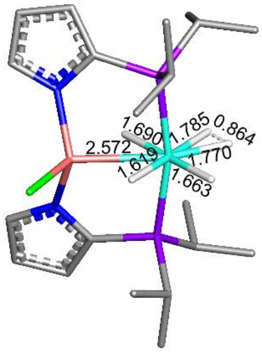

INT4-Ir

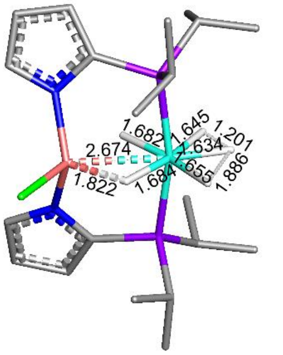

TS3-Ir

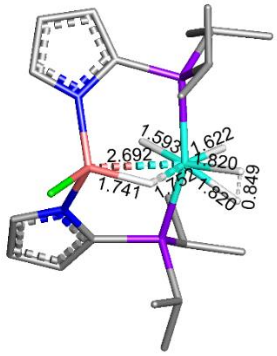

INT5-Ir

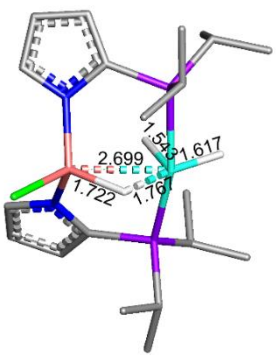

INT6-Ir

Figure S50. Optimized geometries of intermediates and transition states in the H_2 activation by **4-Ir.** Ir is shown in cyan, P in purple, Al in brick red, N in blue, Cl in lime green, C in gray, and H in white. Hydrogen atoms were omitted for clarity except for those on the Ir.

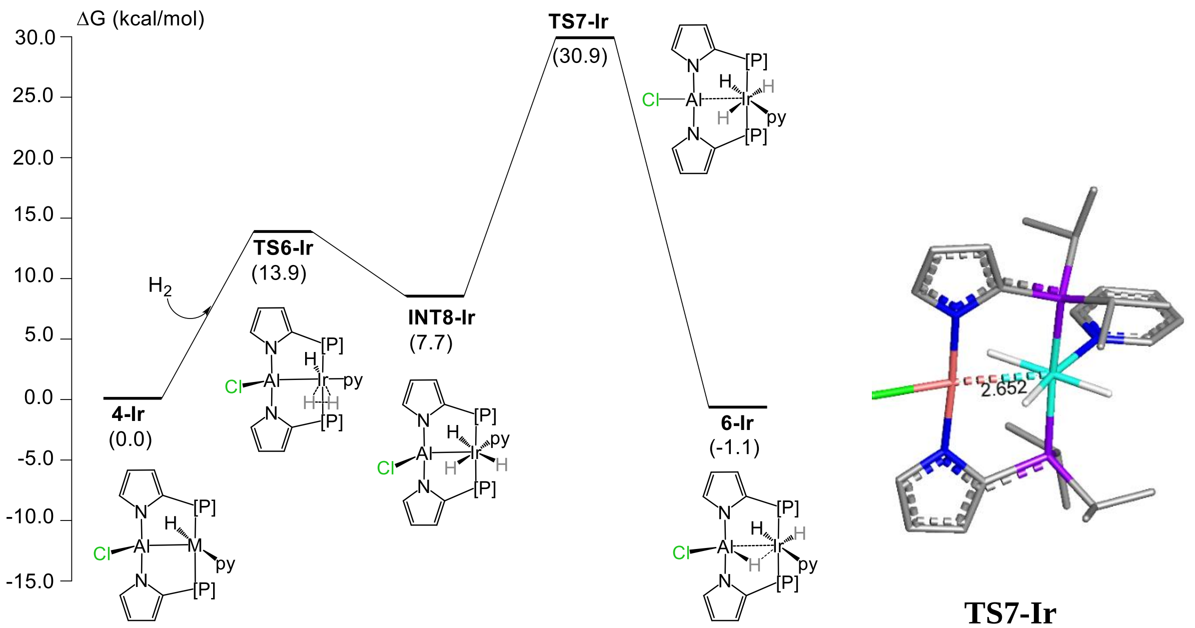

TS7-Ir

Figure S51. Solvent-corrected relative free energy profile for the H_2 activation and intramolecular isomerization from **INT8-Ir** to **6-Ir**. Hydrogen atoms were omitted for clarity except for those on the Ir.

VII. References

[1] Lai, Q.; Cosio, M. N.; Ozerov, O. V. Ni Complexes of an Alane/Tris(phosphine) Ligand Built Around a Strongly Lewis Acidic Tris(N-pyrrolyl)aluminum *Chem. Comm.* **2020**, *56*, 14845-14848.

[2] Cheng, C. Iridium, di-μ-chlorotetrakis[(1,2-η)-cyclooctene]di- *Encyclopedia of Reagents for Organic Synthesis*; Wiley 2014.

[3] Herde, J. L.; Lambert, J. C.; Senoff, C. V. Cyclooctene and 1,5-cyclooctadiene complexes of iridium (I). *Inorg. Synth.* **1974**, *15*, 18-20.

[4] Giordano, G.; Crabtree, R. H. Di-μ-chloro-bis(η^4-1,5-cyclooctadiene) dirhodium(I). *Inorg. Synth.*, **1990**, *28*, 88-90.

[5] APEX3 "Program for Data Collection on Area Detectors" BRUKER AXS Inc., 5465 East Cheryl Parkway, Madison, WI 53711-5373 USA.

[6] Spek, A. L., "PLATON- A Multipurpose Crystallographic Tool" *J. Appl. Cryst.* **2003**, *36*, 7-13.; Spek, A. L., Utrecht University, Utrecht, The Netherlands, **2008**.

[7] Sheldrick, G. M. "SADABS: Program for Absorption Correction of Area Detector Frames", University of Göttingen, **2008**.

[8] (a) Sheldrick, G. M. A short history of SHELX. *Acta Cryst.* **2008**, A64, 112-122. (b) Sheldrick, G. M. Crystal structure refinement with SHELXL. *Acta Cryst.* **2015**, A71, 3-8. (c) Sheldrick, G. M. Crystal structure refinement with SHELXL. *Acta Cryst.* **2015**, C71, 3-8. (d) XT, XS, BRUKER AXS Inc., 5465 East Cheryl Parkway, Madison, WI 53711-5373 USA. (e) Dolomanov, O. V.; Bourhis, L. J.; Gildea, R. J.; Howard, J. A. K.; Puschmann, H. OLEX2: A Complete Structure Solution, Refinement and Analysis Program. *J. Appl. Cryst.* **2009**, *42*, 339-341.

[9] Frisch, M. J.; Trucks, G. W.; Schlegel, H. B.; Scuseria, G. E.; Robb, M. A.; Cheeseman, J. R.; Scalmani, G.; Barone, V.; Petersson, G. A.; Nakatsuji, H.; Li, X.; Caricato, M.; Marenich, A. V.; Bloino, J.; Janesko, B. G.; Gomperts, R.; Mennucci, B.; Hratchian, H. P.; Ortiz, J. V.; Izmaylov, A. F.; Sonnenberg, J. L.; Williams; Ding, F.; Lipparini, F.; Egidi, F.; Goings, J.; Peng, B.; Petrone, A.; Henderson, T.; Ranasinghe, D.; Zakrzewski, V. G.; Gao, J.; Rega, N.; Zheng, G.; Liang, W.; Hada, M.; Ehara, M.; Toyota, K.; Fukuda, R.; Hasegawa, J.; Ishida, M.; Nakajima, T.; Honda, Y.; Kitao, O.; Nakai, H.; Vreven, T.; Throssell, K.; Montgomery Jr., J. A.; Peralta, J. E.; Ogliaro, F.; Bearpark, M. J.; Heyd, J. J.; Brothers, E. N.; Kudin, K. N.; Staroverov, V. N.; Keith, T. A.; Kobayashi, R.; Normand, J.; Raghavachari, K.; Rendell, A. P.; Burant, J. C.; Iyengar, S. S.; Tomasi, J.; Cossi, M.; Millam, J. M.; Klene, M.; Adamo, C.; Cammi, R.; Ochterski, J. W.; Martin, R. L.; Morokuma, K.; Farkas, O.; Foresman, J. B.; Fox, D. J. *Gaussian 16 Rev. C.01*, Wallingford, CT, 2016.

[10] Zhao, Y.; Truhlar, D. G., The M06 suite of density functionals for main group thermochemistry, thermochemical kinetics, noncovalent interactions, excited states, and transition elements: two new functionals and systematic testing of four M06-class

functionals and 12 other functionals. *Theoretical Chemistry Accounts* **2008**, *120*, 215-241.

[11] Andrae, D.; Häußermann, U.; Dolg, M.; Stoll, H.; Preuß, H. Energy-adjustedab initio pseudopotentials for the second and third row transition elements. *Theoretica chimica acta* **1990**, *77*, 123-141.

[12] Francl, M. M.; Pietro, W. J.; Hehre, W. J.; Binkley, J. S.; Gordon, M. S.; DeFrees, D. J.; Pople, J. A. Self-consistent molecular orbital methods. XXIII. A polarization-type basis set for second-row elements. *J. Chem. Phys.* **1982**, *77*, 3654-3665.

[13] Krishnan, R.; Binkley, J. S.; Seeger, R.; Pople, J. A. Self-consistent molecular orbital methods. XX. A basis set for correlated wave functions. *J. Chem. Phys.* **1980**, *72*, 650-654.

[14] McLean, A. D.; Chandler, G. S., Contracted Gaussian basis sets for molecular calculations. I. Second row atoms, *Z*=11–18. *J. Chem. Phys.* **1980**, *72*, 5639-5648.

[15] Clark, T.; Chandrasekhar, J.; Spitznagel, G. n. W.; Schleyer, P. V. R. Efficient diffuse function-augmented basis sets for anion calculations. III. The 3-21+G basis set for first-row elements, Li-F. *J. Comput. Chem.* **1983**, *4*, 294-301.

[16] Spitznagel, G. W.; Clark, T.; von Ragué Schleyer, P.; Hehre, W. J. An evaluation of the performance of diffuse function-augmented basis sets for second row elements, Na-Cl. *J. Comput. Chem.* **1987**, *8,* 1109-1116.

[17] Marenich, A. V.; Cramer, C. J.; Truhlar, D. G., Universal solvation model based on solute electron density and on a continuum model of the solvent defined by the bulk dielectric constant and atomic surface tensions. *J. Phys. Chem. B* **2009**, *113*, 6378-6396.

[18] Fukui, K. The path of chemical reactions - the IRC approach. *Acc. Chem. Res.* **1981**, *14*, 363-368.

[19] Hall, M. B.; Fenske, R. F. Electronic structure and bonding in methyl- and perfluoromethyl(pentacarbonyl)manganese. *Inorg. Chem.* **1972**, *11*, 768-775.

[20] Manson, J.; Webster, C. E.; Pérez, L. M.; Hall, M. B. http://www.chem.tamu.edu/jimp2/index.html.

[21] Glendening, E.; Landis, C.; Weinhold, F. *NBO 6.0*, NBO 6.0, Theoretical Chemistry Institute: University of Wisconsin, Madison, 2013.
